# Combining role-play with interactive simulation to motivate informed climate action: Evidence from the *World Climate* simulation

**DOI:** 10.1371/journal.pone.0202877

**Published:** 2018-08-30

**Authors:** J. N. Rooney-Varga, J. D. Sterman, E. Fracassi, T. Franck, F. Kapmeier, V. Kurker, E. Johnston, A. P. Jones, K. Rath

**Affiliations:** 1 UMass Lowell Climate Change Initiative and Department of Environmental, Earth and Atmospheric Sciences, University of Massachusetts-Lowell, Lowell, Massachusetts, United States of America; 2 System Dynamics Group, MIT Sloan School of Management, Cambridge, Massachusetts, United States of America; 3 Escuela de Ingeniería y Gestión, Instituto Tecnológico de Buenos Aires, Ciudad Autónoma de Buenos Aires, Argentina; 4 Climate Interactive, Washington, DC, United States of America; 5 ESB Business School, Reutlingen, Germany; 6 SageFox Consulting Group, Amherst, Massachusetts, United States of America; Mercator Research Institute on Global Commons and Climate Change gGmbH, GERMANY

## Abstract

Climate change communication efforts grounded in the information deficit model have largely failed to close the gap between scientific and public understanding of the risks posed by climate change. In response, simulations have been proposed to enable people to learn for themselves about this complex and politically charged topic. Here we assess the impact of a widely-used simulation, *World Climate*, which combines a socially and emotionally engaging role-play with interactive exploration of climate change science through the C-ROADS climate simulation model. Participants take on the roles of delegates to the UN climate negotiations and are challenged to create an agreement that meets international climate goals. Their decisions are entered into C-ROADS, which provides immediate feedback about expected global climate impacts, enabling them to learn about climate change while experiencing the social dynamics of negotiations. We assess the impact of *World Climate* by analyzing pre- and post-survey results from >2,000 participants in 39 sessions in eight nations. We find statistically significant gains in three areas: (i) knowledge of climate change causes, dynamics and impacts; (ii) affective engagement including greater feelings of urgency and hope; and (iii) a desire to learn and do more about climate change. Contrary to the deficit model, gains in urgency were associated with gains in participants’ desire to learn more and intent to act, while gains in climate knowledge were not. Gains were just as strong among American participants who oppose government regulation of free markets–a political ideology that has been linked to climate change denial in the US–suggesting the simulation’s potential to reach across political divides. The results indicate that *World Climate* offers a climate change communication tool that enables people to learn and feel for themselves, which together have the potential to motivate action informed by science.

## Introduction

Scientific evidence supporting an urgent need to mitigate anthropogenic climate change is clear [1]. Yet, social science data are also clear: around the world, public understanding and concern over climate change are not commensurate with the risks we face [2]. Although public opinion favoring action to mitigate climate change is increasing, it is not strong enough to generate the individual and governmental actions necessary to meet international climate goals [3, 4]. The decision by the US to withdraw from the Paris accord [5] and shifts in US federal policy towards production of fossil fuels [6] further threaten global efforts to mitigate climate change [7]. Communication tools that are both scientifically rigorous and that motivate informed action on climate change are urgently needed [8].

Many efforts to communicate the risks of climate change are grounded, explicitly or implicitly, in the information deficit theory of risk communication, which posits that providing people with more and better information about the reality, causes, and risks of climate change should motivate them to take appropriate action [9]. However, communication strategies based on the deficit model have failed to close the gap between scientific and public understanding for climate change and many other settings, (e.g., [10–15]). Three factors play a role: First, humans can only process the dynamic interactions of two to three variables at a time [16]–a limitation clearly exceeded by the complexity of the climate system. While climate dynamics are strongly conditioned by feedbacks, accumulations, nonlinearities, and time delays, even highly educated adults are unable to infer the behavior of even the simplest dynamic systems [17]. Second, affective responses—the type and intensity of emotions people experience—play an important role in risk perception and decision making. Typical presentations on the causes and risks of climate change often fail to elicit affective responses or motivate action to combat it [18]. Third, social forces both enable and constrain individual action, especially in collective action settings such as climate change [19, 20]. Individuals who share ties with members of social groups that dismiss climate change are also likely to dismiss it [20], while the belief that other similar people take action increases behaviors to combat climate change [19].

Effective risk communication enables people to learn for themselves through experience and experimentation rather than being told by experts [9, 21]. Yet, for climate change and many other important issues, controlled experiments are impossible, unethical or prohibitively expensive. Long delays in the response of the climate to greenhouse gas (GHG) emissions means experience will come too late. In such settings, simulation becomes the main—perhaps the only—way we can discover for ourselves how complex systems work and what the impact of different policies might be, thus integrating science into decision making. Effective simulation experiences should not only be rigorously grounded in the best available science but also engage the often messy, imperfectly rational, socially conditioned emotions and behavior of participants. For these reasons, simulations that integrate rigorous models of physical systems with role-plays that represent the social dynamics of decision-makers are now common in aviation, power plant operations, medicine, the military, and other high-risk settings [22–25].

Here, we assess the impact of one such simulation, *World Climate*, in which participants take on the roles of UNFCCC negotiators and use the C-ROADS interactive computer model to get immediate feedback on the expected climate impacts of their decisions based on current scientific understanding [26, 27]. *World Climate* is widely used around the world—more than 42,000 people in 77 countries participated in it between August 2015 and May 2018 and it is designated as an official resource for schools in France [28], Germany (Beule, personal communication) and South Korea [29]—indicating the importance of an assessment of its impact. We explore whether participating in *World Climate* is associated with gains in knowledge of climate science, affective responses to climate change, desire to learn more, and intent to mitigate climate change in the real world. We also examine whether increased desire to learn and act on climate change are associated with gains in knowledge or with gains in affect.

The paper is organized as follows: we first describe the *World Climate* role-play, the C-ROADS climate policy simulation model, and the learning model we seek to test. We then describe the sample of *World Climate* sessions we analyze and the pre- and post-simulation survey design. We use exploratory factor analysis (EFA) to develop constructs representing latent variables measured by the surveys, finding constructs relating to climate change knowledge, affect, desire to learn more about climate change, and intent to take action to combat it. We analyze the gains in these constructs by examining the differences in their values from the pre- to post-simulation survey and then analyze associations among gains in constructs using regression analyses. These analyses revealed that gains in affect, but not knowledge, are linked to a desire to learn and do more to address climate change. We describe the effect of political ideology on *World Climate* outcomes, finding that participants who oppose government regulation of free markets gained at least as much as those who support regulation. We turn next to sensitivity analysis to examine threats to external validity potentially arising from (i) potential selection bias because individuals in some of the sample sessions chose to participate while others participated as part of a required curriculum unrelated to climate change, and (ii) potential bias associated with voluntary response sampling. We close with general discussion, including limitations and extensions.

## The *World Climate* simulation

Participants in *World Climate* take on the roles of parties to the UN climate negotiations and are challenged to create an international agreement that limits warming by 2100 to well below 2 °C above preindustrial levels. As in the UNFCCC process, participants specify Nationally Determined Contributions (NDCs) for the parties they represent while seeking to influence the other parties through face-to-face negotiations. Participants’ proposals are then entered into the C-ROADS climate policy model [26, 27], which provides immediate feedback about the expected climate outcomes of those decisions.

**Fig 1:**
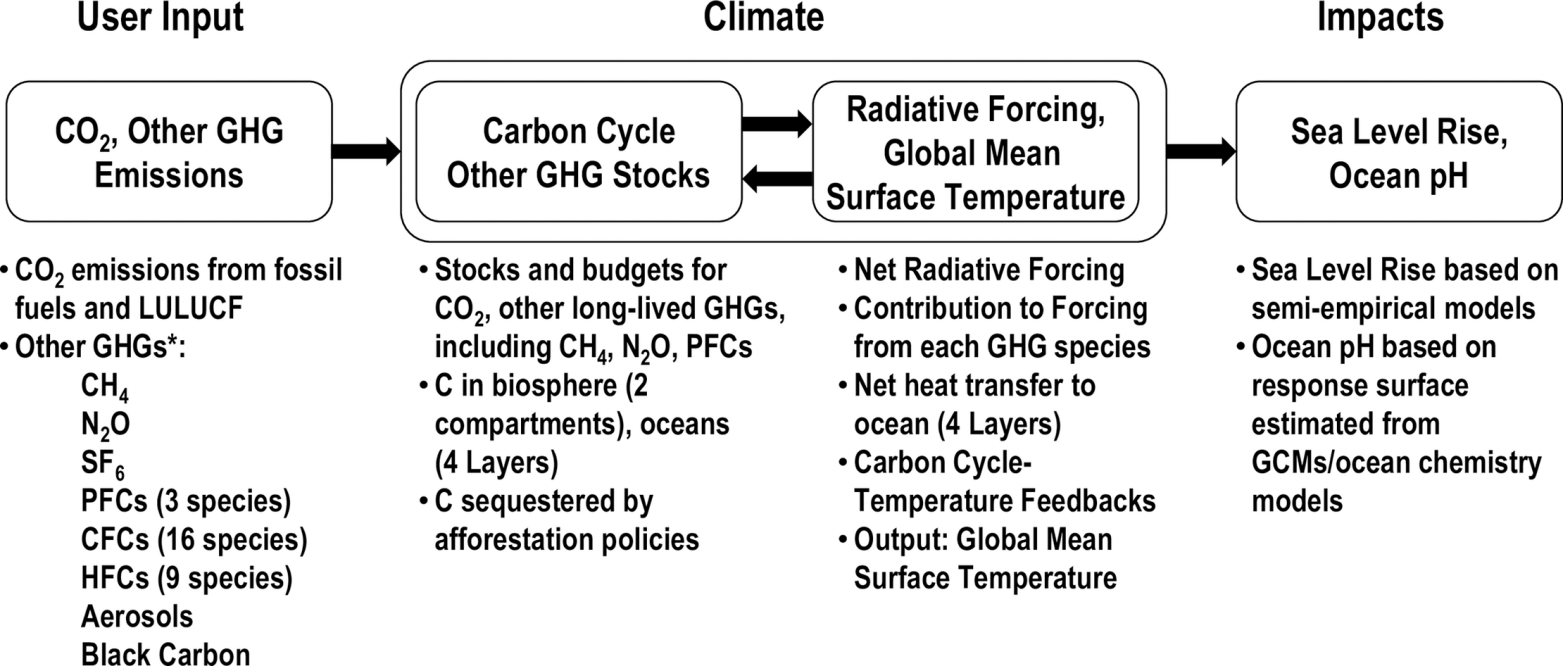
Overview of C-ROADS model, adapted from Sterman et al. [27]. Participants in *World Climate* specify CO_2_ emissions from fossil fuels or land use, land use change, and forestry (LULUCF).

**Fig 2:**
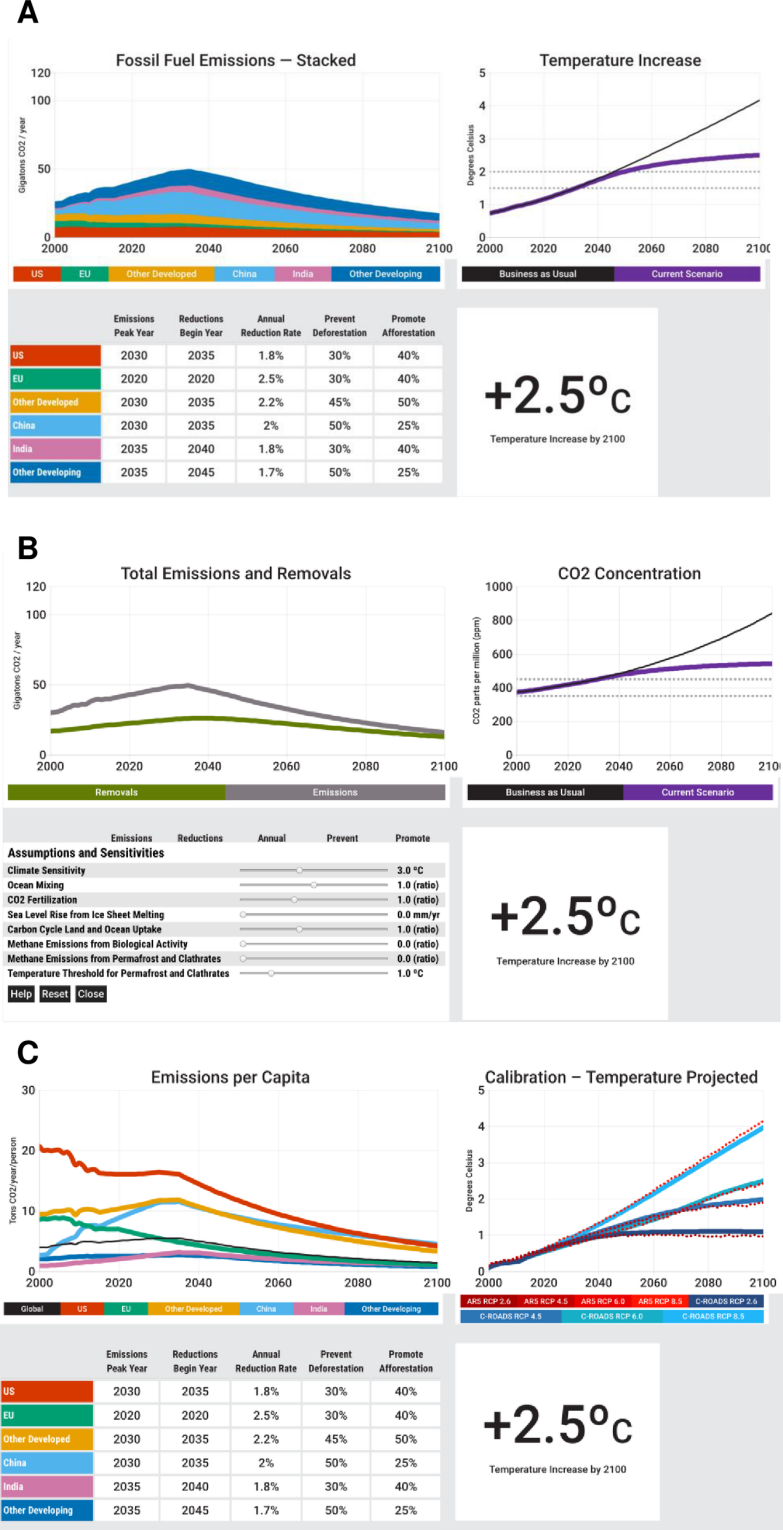
Screenshots from the C-ROADS *World Climate* computer model. Panel A is the six-region *World Climate* interface through which participants enter decisions, including the year they choose (if any) to halt the growth of emissions, begin to decline and the annual rate of decline (%), with changes in deforestation and afforestation (on scales of 0–100%, with 0 being business-as-usual and 100% being the maximum possible effort). The model immediately displays the resulting CO_2_ emissions trajectories (panel A left), global mean surface temperature anomaly relative to pre-industrial levels (panel A right) and other impacts. B: Screenshot showing CO_2_ emissions and net removals for the scenario entered in panel A (panel B left), illustrating the “carbon bathtub” [33], i.e., that the stock of CO_2_ in the atmosphere accumulates anthropogenic CO_2_ emissions less the net CO_2_ flux from the atmosphere to biosphere and oceans. Users can carry out a wide range of sensitivity tests by choosing values for parameters affecting, e.g., climate sensitivity and the strength of both positive (e.g., Arctic methane) and negative (e.g., CO_2_ fertilization) feedbacks in the climate system (panel B, bottom left). C: C-ROADS enables users to explore economic and population data linked to emissions (e.g., GHG emissions per capita shown in panel C, left), and to compare the fit between the model and historical data for GHG concentrations and to projected global surface temperature in CMIP5 models through 2100.

C-ROADS is a member of the family of simple climate models (SCMs) [30], consisting of a system of differential equations that represent the carbon cycle; budgets and stocks of GHGs, including CO_2_, CH_4_, N_2_O, SF_6_, PFCs, CFCs, HFCs, aerosols and black carbon; radiative forcing and the heat balance of the Earth; exchange and transport of carbon and heat between the atmosphere and ocean; and climate change impacts (Fig 1) [26, 27]. The carbon cycle includes compartments for stocks of carbon in the atmosphere, biosphere, soils, and the ocean (which is divided into four layers). Users specify a fossil fuel emissions pathway for the nation or bloc they represent, and policies to reduce deforestation or promote afforestation. C-ROADS enables participants to examine the expected effects of these decisions including atmospheric GHG concentrations, global temperature change, global mean sea level rise, and ocean acidification. C-ROADS can be configured to enable emissions inputs for one, three, six, or fifteen different nations and blocs of nations, in all cases collectively adding up to global emissions. Fig 2 shows the C-ROADS user interface for six regions.

C-ROADS is designed to be transparent, to be accessible to non-specialists, and to enable users to build an understanding of the climate system rather than using the model as a black box. C-ROADS closely replicates historical data from 1850 and CMIP5 model projections through 2100 across a wide range of Representative Concentration Pathways (RCPs) (Fig 2C) [26, 31]. Although model parameters are based on accepted peer-reviewed science, users are not compelled to accept the default values and can adjust assumptions including climate sensitivity, CO_2_ fertilization feedbacks, and Arctic methane emissions so that they can explore the sensitivity of results to uncertainty (Fig 2B). Users can input any future emissions scenarios they wish and get immediate feedback on the expected global climate outcomes of those scenarios. C-ROADS has been used by policymakers [32], is freely available for online or offline use (https://www.climateinteractive.org), and runs in about one second on laptops and other devices–characteristics well suited for interactive exercises such as *World Climate*.

**Fig 3:**
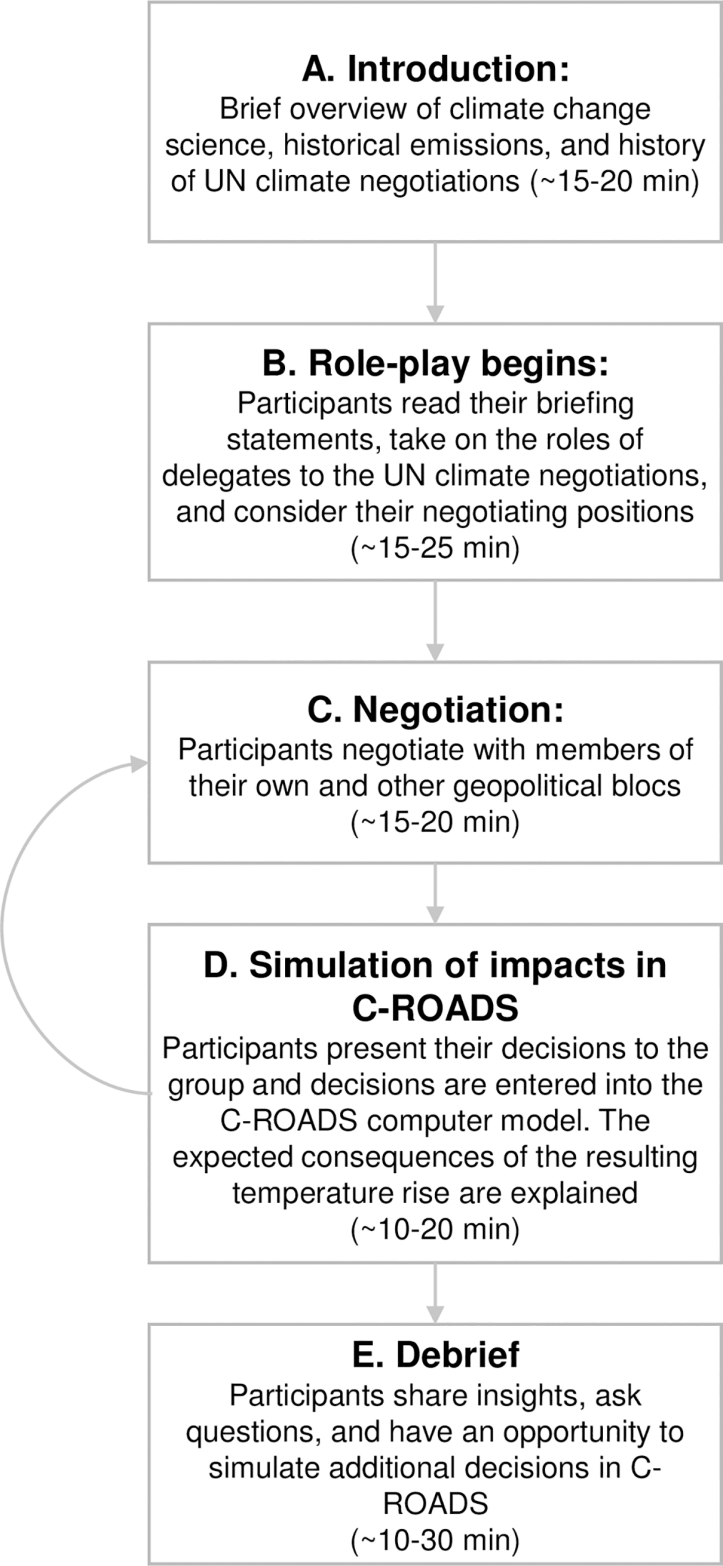
Sequence of a *World Climate* simulation.

All sessions in our sample followed the same protocol (Fig 3), described in detail in Sterman et al. [34]. Participants are first assigned to one of six delegations to the negotiation (the USA, European Union, China, India, Other Developed Nations, and Other Developing Nations) and receive the briefing memo for their nation or bloc (S1 Appendix). The facilitator, playing the role of the UN Secretary General or UNFCCC Executive Secretary, gives a brief overview on climate change, historical GHG emissions, the context of current UN climate negotiations, and expected consequences of business-as-usual emissions trajectories including sea level rise, ocean acidification, and increasing risks of extreme weather, crop yields and other impacts (https://www.climateinteractive.org/programs/world-climate/instructor-resources/slide-sets/). The facilitator then presents the key policy decisions participants are charged with: specifying their bloc or nation’s fossil fuel emissions pathway through 2100; their effort to protect against deforestation and/or promote afforestation; and how much money, if any, they will contribute to or seek from the UN Green Climate Fund [35]. None of the materials (briefing memos or presentation slides) are prescriptive. Rather, participants are free to make any decisions they wish as they engage in face-to-face negotiations with the other parties. A short video showing excerpts from a *World Climate* session is available at https://www.climateinteractive.org/programs/world-climate/.

The first round of negotiations ends with a plenary session in which a representative from each delegation delivers a short speech describing their pledge and negotiating position, including concessions they seek from the other parties. The pledges are then entered into C-ROADS, which immediately displays the climate impacts expected to result from the collective emissions pathways chosen by the participants (Fig 2A). C-ROADS also enables the participants to see the impact of the emissions pathways of each bloc on emissions per capita, the emissions intensity of the economy, cumulative emissions, and other indicators that bear on the debate over the principle of “common but differentiated responsibilities”.

In our experience, the first round of pledges always falls short of the emissions reductions required to limit expected warming to 2 °C and are often qualitatively similar to the actual pledges that emerged from the Paris Agreement, leading to warming of approximately 3.3 °C by 2100. Participants often express surprise that the impact of their pledges is not greater and ask many questions about the structure and dynamics of the climate system as they seek to understand why the simulation results differ from their expectations. C-ROADS is then used to show the “bathtub dynamics” of CO_2_ accumulation in the atmosphere [21, 33], with atmospheric CO_2_ concentrations continuing to rise as long as emissions exceed the net flux of CO_2_ from atmosphere to the ocean and biosphere (Fig 2B). The facilitator explains additional impacts expected at the level of warming obtained, for example, showing maps depicting sea level rise for coastal cities in different geographic areas and expected impacts on global food production, freshwater supplies, wildfires, biodiversity and other impacts (all described in the slide deck available with *World Climate*). Participants then enter a second (and, if time allows, third) round of negotiation, each followed by simulation of the new proposals. The role-play concludes with a debriefing conversation in which participants are actively engaged with one another and with the C-ROADS model (Fig 3).

## Hypothesized learning model

**Fig 4:**
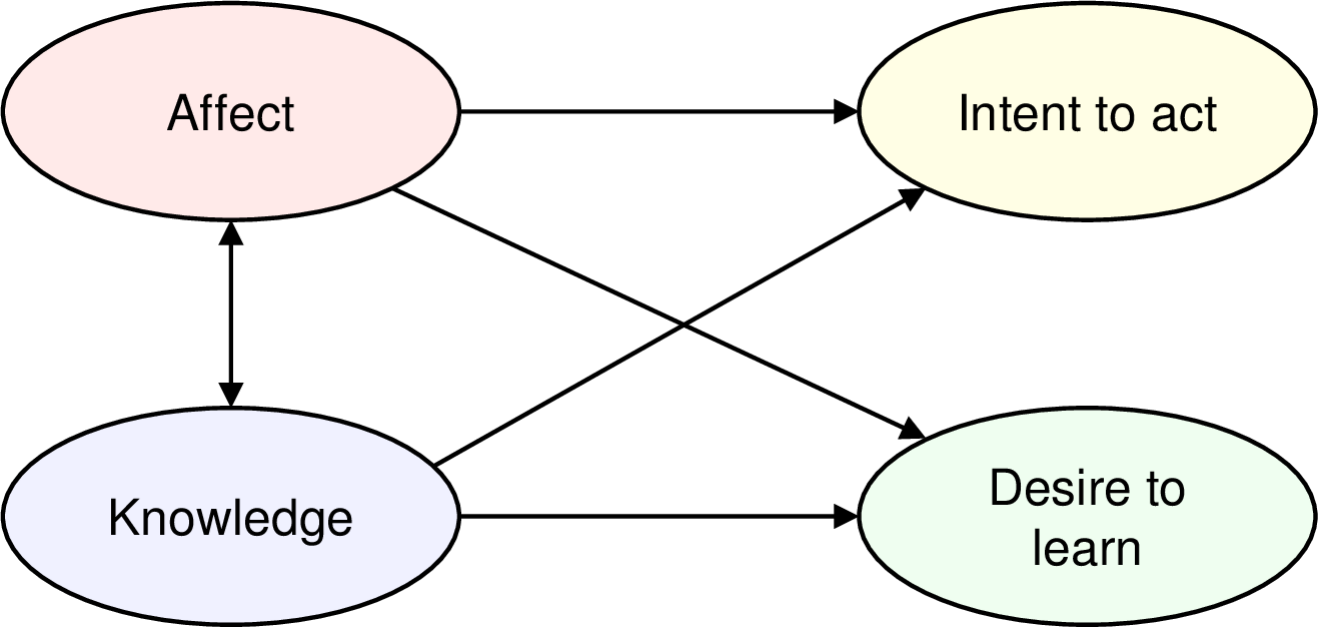
Theoretical model of learning for action through the *World Climate* simulation.

We seek to test whether *World Climate* helps people learn about climate change science while motivating them to learn more and increasing their intent to take action. Fig 4 summarizes prior theory showing how gains in knowledge, affect, desire to learn more, and intent to take action relate to one another. If the information deficit model of learning [9] were correct, then outcomes such as a desire to learn more and intent to take action (“Desire to Learn” and “Intent to Act,” respectively) would arise from gains in knowledge resulting from exposure to information about climate change. However, knowledge may neither function alone nor be sufficient to drive action. For example, knowledge about the causes and impacts of climate change is positively correlated with concern, an affective response [36]. Further, climate change knowledge and affect are thought to have a bidirectional, reinforcing relationship [37] (shown as links between “Knowledge” and “Affect,” Fig 4). Affect is also important in risk perception and support for climate action [38, 39], suggesting that changes in “Intent to Act” and “Desire to Learn” may be affected more strongly by affect than knowledge.

To gain insight into these issues we collected open-ended responses from participants in *World Climate* sessions (see below for description of the sample and data collection protocol). While a comprehensive analysis of these responses is beyond the scope of the current study, these examples illustrate common themes expressed by participants in the study sample. We report examples from sessions that were a required component of courses or programs unrelated to climate change to avoid potential selection bias that might arise in sessions that participants joined voluntarily, as such participants could be predisposed to favor action to address climate change. These sessions include a program for low-income high school students (Upward Bound, Boston University); a required activity for high school students in Miami, Florida; an Honors Seminar for undergraduate students at UMass Lowell; undergraduate and graduate business students at Reutlingen University, Germany; and the Executive MBA program at MIT. Typical responses to the question, “How has participating in the *World Climate* simulation affected your understanding of climate change, if at all?” include:

“Now I know the facts, causes, and effects.”–High school student, Boston University Upward Bound.

“I have an increased understanding of the urgency and level of effort required to make a positive impact.”–Undergraduate student, UMass Lowell.

“It was very eye-opening. Current issues regarding climate change are very clear and the time-pressure understood.”–Business student, Reutlingen University.

“It changed my mental model dramatically.”—Executive MBA participant, MIT.

Other comments illustrate gains in affective engagement (and, in some cases, their link to gains in knowledge resulting from the role-play), e.g.:

“I was surprised how angry I became.”—Undergraduate student, UMass Lowell.

“Alarmed but hopeful.”–High school student, Miami, Florida.

“Empowered because I'm a part [of] something bigger than me.”–High school student, Boston University Upward Bound.

“I am much more concerned now about the climate change, and much more aware about the specific actions each of us has to take to make a contribution into and have an impact on saving this world for our children.”—Executive MBA participant, MIT.

Responses also suggest that gains in affect and knowledge were associated with both gains in intent to act and desire to learn more. Responses to the question, “Has participating in *World Climate* affected how motivated you are to address climate change? If so, what do you plan to do?” included:

“I want to do more research into possible solutions for climate change.”—Undergraduate student, UMass Lowell.

“Yes, I am more motivated to learn more and contribute to change.”—Undergraduate student, UMass Lowell.

“It has motivated me to inform others who may not be aware of what climate change is.”–High school student, Boston University Upward Bound.

“I plan to take more action to decrease my carbon footprint. I could walk more, use LED lights, turn off lights + also most importantly inform other people about climate change so they are also aware.”–High school student, Boston University Upward Bound.

“Stronger desire to learn more and have the tools to change my peers’ minds.”—Executive MBA participant, MIT.

Responses to open-ended questions suggest participants experienced increases in their affective engagement, desire to learn more about climate change and their intention to take action. Several responses also point to interactions among knowledge, affect, and intent (e.g., “*I have an increased understanding of the urgency*,*”* and “*I am much more concerned now about the climate change*, *and much more aware about the specific actions each of us has to take…”*). Together with prior research on climate change communication [9, 13–15], these responses suggest gains in participants’ climate change knowledge and affect, not knowledge alone, contribute to gains in their desire to learn more and their intent act.

## Sample and data collection

Our sample consisted of 39 *World Climate* sessions conducted between September 2015 and October 2017 in locations in North and South America, Europe and Africa, with a total of 2,042 participants (Table 1). These sessions are broadly representative of the wide spectrum of educational and cultural settings in which *World Climate* is used, ranging from early secondary school to graduate school to sessions open to the public; participant ages from 11 to more than 75; and heterogeneous participant backgrounds from no prior education or interest in climate change to professionals whose career focus is climate change and sustainability.

**Table 1:** Overview of *World Climate* sessions and participants in this study.

*ID*	*Location*	*Institution*	*Educational setting*	*Age range*^*1*^	*Mode of age*^*1*^	*% Males*^*1*^	*Facilitator training*^*2*^	*Self-selection*^*3*^
1	Middletown DE USA	St. Andrews School	Secondary school	14–50	14–17	56%	Web	No
2	Cali, Colombia	Universidad del Valle	Higher education	18–75	18–24	44%	TM	No
3	Buenos Aires, Argentina	Instituto Tecnologico Buenos Aires	Higher education	18–76+	18–24	51%	TM	Yes
4	Marrakech, Morroco	Climate Interactive	Faculty	18–75	51–75	65%	TM	Yes
5	Miami FL USA	Upward Bound	Secondary school	14–76+	14–17	49%	Web	No
6	Lowell MA USA	UMass Lowell	Higher education (STEM)	18–50	18–24	56%	TM	Yes
7	Seattle WA USA (and online)	Pinchot University	MBA online class	18–75	25–35	26%	TM	Yes
8	Shaker Heights OH USA	Hathaway Brown School	Secondary school	14–76+	14–17	0%	Web	Yes
9	Dublin, Ireland	Dublin City University	University faculty, staff, students	18–75	51–75	48%	Web	No
10	Lowell MA USA	UMass Lowell	Higher education (STEM)	18–35	18–24	75%	TM	No
11	Chapel Hill NC USA	University of North Carolina	Graduate students	25–50	25–35	69%	TM	Yes
12	Lowell MA USA	UMass Lowell	Informal higher education	18–24	18–24	81%	TM	Yes
13	Reutlingen, Germany	Reutlingen University	Higher education	18–35	18–24	37%	TM	No
14	Cambridge MA	Cambridge Rindge Latin	Secondary school	11–24	14–17	58%	TM	No
15	Buenos Aires, Argentina	Instituto Tecnologico Buenos Aires	Higher education	14–50	18–24	56%	TM	Yes
16	Nairobi, Kenya	Climate Interactive	Higher education	18–50	25–35	44%	TM	Yes
17	Nairobi, Kenya	Climate Interactive	Higher education	18–75	25–35	48%	TM	Yes
18	Cambridge, MA USA	MIT Sloan	Executive MBA students	25–75	36–50	30%	TM	No
19	Cape Town, South Africa	Climate Interactive	University researchers	18–75	36–50	55%	TM	Yes
20	Stellenbosch University, Stellenbosch South Africa	Climate Interactive	Graduates and professionals	18–75	25–35	44%	TM	Yes
21	Limuru, Kenya	Climate Interactive	Higher education	18–24	18–24	79%	TM	No
22	Lagos, Lagos, Nigeria	Climate Interactive	Faculty members and professionals	25–75	36–50	36%	TM	Yes
23	Lagos, Lagos, Nigeria	Climate Interactive	Higher education	18–50	25–35	44%	TM	Yes
24	Abuja, Nigeria	Climate Interactive	Higher education	18–75	25–35	26%	TM	Yes
25	Victoria Island, Lagos, Nigeria	Nat’l Centre Tech Management	Higher education	18–75	36–50	0%	Web	Yes
26	Lowell MA USA	Garden Club Fed MA	Adults	36–76+	51–75	100%	TM	Yes
27	Albany, NY USA	U. Albany	Higher education	18–75	25–35	38%	F2F	No
28	Mesa, AZ, USA	Mesa Comm. College	Higher education	18–75	18–24	72%	Web	Yes
29	Cambridge, MA USA	MIT Sloan	EMBA students	14–75	36–50	64%	TM	No
30	Boston, MA USA	BU Upward Bound	Secondary school	11–24	14–17	31%	TM	No
31	Miami FL USA	Cushman High School	Secondary school	14–75	14–17	44%	F2F	No
32	Portland, ME USA	Council for Opportunity in Education	Educators	18–75	51–75	20%	TM	Yes
33	Charleston, WV USA	Council for Opportunity in Education	Educators	18–50	25–35	56%	TM	Yes
34	Cambridge, MA USA	Harvard Business School	Higher education	25–75	25–35	43%	Web	No
35	Cambridge, MA USA	MIT Sloan	Higher education	18–75	25–35	46%	TM	Yes
36	Cambridge, MA USA	MIT Sloan	Asian School of Business MBA students	18–50	25–35	65%	TM	No
37	Cambridge, MA USA	MIT Sloan	Higher education	18–50	18–24	40%	TM	Yes
38	West Orange, NJ USA	Liberty Middle School	Secondary school	11–76+	11–13	51%	Web	No
39	Boston, MA USA	Assoc. Grantmakers MA	Adults	18–35	18–24	30%	TM	Yes

^*1*^Age and gender data refer to usable cases. Note: the survey asked participants to select an age range (e.g., 25–35) rather than entering their age (see S2 Appendix for full survey).

^*2*^Indicates training and expertise of the session facilitator: ‘TM’ = project team members; ‘Web’ = facilitators who only received training via online materials or webinars; ‘F2F’ = facilitators who attended a face to face training

^*3*^Indicates whether or not participants chose to participate in a climate change-related activity or course (yes) or were required to participate as part of a program or course unrelated to climate change (no).

### Methods

Sessions lasted between 1.5 and 3 hours. All sessions in our sample were face-to-face events with the exception of one synchronous online session (Table 1). Facilitators included the authors and other individuals who learned how to facilitate sessions after participating in *World Climate* or learning about it online. Facilitators in all sessions used the same materials, including: two-page briefing memos for participants (provided in S1 Appendix), with information about past and projected greenhouse gas emissions and a brief overview of each nation or bloc’s negotiating goals, public opinion about climate change, risks of climate impacts, and opportunities for climate action; slides to introduce and debrief sessions; and the C-ROADS computer model. All of these materials, including C-ROADS, a detailed facilitator’s guide, and a video explaining how to facilitate *World Climate* are freely available online (https://www.climateinteractive.org/programs/world-climate/).

### Survey instruments and data processing

We used a pre-/post-survey design to assess the impact of *World Climate*. To reduce the likelihood that external events influenced participants’ responses, the pre- and post-surveys were administered within a short time period (a few minutes to several days) before and after each session. The surveys are provided in the Supporting Information (S2 Appendix) and were approved by the institutional review boards of UMass Lowell and MIT (Protocols 16-049-ROO-XPD and 1702833248, respectively). We obtained informed consent through both verbal and written statements. Participants were informed that survey completion was voluntary, the individual results confidential, and, if *World Climate* was part of an academic course or program, that their responses had no influence on their academic standing (S2 Appendix).

The pre- and post-surveys include items designed to assess participants’ knowledge about climate change, their affective responses to it, their intent to learn and do more to address it, as well as questions on participants’ sociodemographic characteristics. The surveys include items used in prior work to assess knowledge of climate science and beliefs about the reality and causes of climate change [40]. Other knowledge-related items elicited participants’ beliefs about the impacts of climate change, and their understanding of the dynamics of CO_2_ accumulation in the atmosphere, which prior research [17, 33, 41] shows to be widely misunderstood. We used semantic differential scales [42] to assess participants’ affective response to climate change by asking them to locate how they feel about climate change on scales spanning emotional poles, for example, hopeless to hopeful, discouraged to empowered, and indifferent to engaged. Participants’ perceived socioeconomic status was assessed using language adapted from Goodman et al. [43, 44]. The post-survey included additional questions eliciting participants’ reactions to *World Climate*, including whether it influenced their motivation to address climate change or their desire to learn more about climate science, technological solutions, economics, and policy options. The post-survey also included optional open-ended questions where participants could comment on how the simulation affected their understanding of climate change, their affective responses and motivation to address it. All survey questions were tested by soliciting feedback from five educators using *World Climate*, two educational psychologists, and ten undergraduate students who had not participated in the simulation.

Respondents were included in the analysis if they reported no previous experience with *World Climate*, answered ≥80% of the pre- and post-survey questions analyzed, and provided pre- and post-surveys that could be matched to each other. Across the 2,042 participants in our sample, 75% responded to the pre-survey (with a range of 24–100% per session), 62% responded to the post-survey (range: 24–100%) and 42% of all participants met all of the criteria used to define ‘usable cases’ (range: 12–92%; Table 2). The full dataset is available from the Dryad Digital Repository at https://doi.org/10.5061/dryad.343nt5s.

**Table 2:** Participants and usable cases for each *World Climate* session. The number of pre-, post-, and matched surveys obtained, expressed as a percentage of the total number of participants in a given session.

*ID*	*Participants*	*% Pre-Surveys*	*% Post-Surveys*	*% Matched Surveys*^*3*^	*% Usable*^*1*^
1	18	89%	44%	33%	33%
2	45	100%	100%	100%	13%
3	40	98%	98%	98%	38%
4	60	72%	32%	23%	15%
5	200	62%	48%	16%	12%
6	18	100%	67%	61%	50%
7	26	88%	77%	62%	62%
8	71	90%	38%	28%	27%
9	20	100%	95%	75%	70%
10	14	86%	86%	86%	71%
11	36	94%	92%	86%	83%
12	16	100%	88%	88%	69%
13	55	35%	35%	35%	33%
14	46	98%	72%	65%	61%
15	39	95%	92%	62%	54%
16	25	72%	72%	52%	24%
17	27	100%	100%	74%	44%
18	180	64%	44%	44%	43%
19	20	100%	95%	95%	80%
20	25	64%	68%	64%	56%
21	30	80%	80%	80%	37%
22	45	24%	24%	24%	13%
23	35	46%	46%	46%	26%
24	75	73%	73%	73%	44%
25	19	84%	84%	84%	58%
26	30	83%	80%	70%	47%
27	45	53%	56%	51%	47%
28	23	78%	78%	78%	78%
29	120	97%	81%	54%	52%
30	90	97%	93%	90%	78%
31	40	100%	63%	40%	28%
32	12	100%	100%	92%	75%
33	12	58%	67%	50%	42%
34	270	59%	34%	31%	31%
35	60	80%	77%	70%	63%
36	50	96%	94%	82%	78%
37	9	100%	89%	78%	56%
38	72	64%	61%	51%	43%
39	24	96%	96%	92%	92%
	**Min**	**24%**	**24%**	**16%**	**12%**
	**Max**	**100%**	**100%**	**100%**	**92%**
	**Weighted Mean**	**75%**	**62%**	**52%**	**42%**

^*1*^Usable cases, defined as the number of participants with no prior experience with *World Climate*, and who provided matched pre- and post-surveys with >80% of survey items completed.

## Familywise error rate: Bonferroni correction

As described below, we test multiple hypotheses to examine whether participants experienced gains in knowledge, affect, and desire to learn and do more; how those gains were related to each other, if at all; the potential influence of political views; and potential threats to validity. We apply a Bonferroni correction to reduce the likelihood of erroneously finding statistically significant results when multiple hypotheses are tested. For any conventional threshold for statistical significance, α, the Bonferroni correction is α_adjusted_ = α/N, where N is the number of tests carried out. We conduct a total of 104 tests for statistical significance across the full set of t-tests and regression analyses. The Bonferroni-adjusted significant levels for α = 0.05, 0.01, and 0.001 are therefore *p <* 4.8 x 10^−4^, *p <* 9.6 x 10^−5^, and *p <* 9.6 x 10^−6^, respectively. That is, we reject the null hypothesis that *World Climate* had no impact for each individual test we conduct only if the probability of erroneously doing so is *p <* 4.8 x 10^−4^_._ Doing so yields a familywise error rate—the probability of erroneously rejecting any true null hypothesis across the full set of tests conducted—of 0.05. All statistical analyses were conducted using IBM SPSS Statistics, version 24.

## Exploratory factor analysis (EFA)

We used exploratory factor analysis (EFA) of survey results to test the learning model (Fig 4), specifically, to test for the presence of constructs capturing knowledge of climate change, affective responses to the issue, and participant intent to take action to address it. EFA reduces the dimensionality of the dataset by identifying latent variables in the surveys, if any, [45] and enables us to assess whether they correspond to the constructs in the learning model.

### Methods

We extracted factors separately from pre- and post-survey items, comparing results and testing whether the factors identified were consistent across the pre- and post-surveys. Separate pre- and post-survey factor extraction is warranted because pre- and post-responses from a given participant are not independent from each other. This approach also enabled development of constructs from questions that are only included in the post-survey, such as questions addressing participants’ desire to learn more about climate change.

We used principal axis factoring with orthogonal (varimax) rotation for factor extraction and the Kaiser-Meyer-Olkin (KMO) measure of sampling adequacy to assess the potential for extracting distinct, reliable factors. KMO measures > 0.65 were considered to indicate that correlations among individual items were appropriate for factor analysis [46]. We used Bartlett’s test of sphericity to test whether correlations among items included in the EFA were significantly different from zero (*p* < 0.05). The validity of extracted factors was tested using several methods, including the Kaiser criterion (i.e., eigenvalue > 1; [47], a scree test [48], and the interpretability of factors given prior theory [49]). Individual items were retained in a given factor if (1) factor loading was > 0.45 for the focal item and < 0.45 for other items [49], (2) reliability testing yielded Cronbach’s α > 0.7; (3) deletion of an item did not result in an increase of Cronbach’s α; and (4) inclusion of the item was supported by separate factor extraction from both pre- and post-survey items.

We used factor-based scores as a simple, intuitive approach to combine survey responses for all items that fell within a given construct [49]. The surveys include questions with responses that ranged from binary responses to five-point Likert scales. To weight each survey question equally, we recoded all responses to a scale of zero to one, with zero being the lowest possible response value and one being the highest possible response value for each item. Factor-based scales for each construct were then calculated by taking the mean of the recoded response values for all items that fell within that construct. Thus, each construct had a minimum possible value of zero and a maximum possible value of one.

### Results and discussion

Factor analysis revealed four factors common to both pre- and post-surveys (Tables 3 and 4). One, which we denote ‘*Impacts*,’ combines items assessing participant knowledge about the risks to ecosystems and human welfare posed by climate change. Two factors relate to affective responses to climate change. One, which we denote ‘*Urgency*,*’* includes six items assessing participants’ feelings of worry, guilt, fear, alarm, outrage and anger about climate change, and the extent to which climate change is personally important to them. The second affect-related factor, which we denote *‘Hope*,*’* arises from items assessing whether people feel hopeful or hopeless, empowered or discouraged, that is, agency—whether they believe change is possible and individual action can make a difference. Survey items asking about the likelihood participants will take action to reduce their personal carbon footprint, discuss climate change with family, friends, or peers, or take political action on climate change loaded onto a factor we denote *‘Intent*.*’* Lastly, the post-surveys include five items addressing whether the simulation altered participants’ desire to learn more about climate change, all of which loaded onto a fifth factor we denote *‘Desire to Learn More’* (Table 4).

**Table 3:** Factor loadings and communalities based on principal axis factor analysis with orthogonal rotation (varimax with Kaiser normalization) from pre-survey item analysis (N = 1,059; Kaiser-Meyer-Olkin measure of sampling adequacy = 0.89; Bartlett's test of sphericity *p* <1E-9).

Pre-Survey results	Factor					Communalities	
	1	2	3	4	5	Initial	Extraction
*Eigenvalue*	5.90	2.20	1.71	1.15	0.79		
*% of variance*	32.79	12.20	9.49	6.37	4.38		
*Survey item*							
*How worried are you about climate change*?	0.631					0.547	0.577
*Feelings about climate change—Not Guilty to Guilty*	0.453					0.216	0.239
*Feelings about climate change—Calm to Outraged/Angry*	0.665					0.422	0.493
*Feelings about climate change—Unconcerned to Alarmed*	0.734					0.550	0.639
*Feelings about climate change—Not Afraid to Very Afraid*	0.720					0.483	0.567
*How important is the issue of climate change to you personally*?	0.586					0.572	0.598
*Feelings about climate change—Hopeless to Hopeful*				0.706		0.367	0.522
*Feelings about climate change—Discouraged to Empowered*				0.802		0.371	0.654
*Impacts of climate change—Increased temperatures globally*		0.562				0.310	0.357
*Impacts of climate change—Increased incidence and intensity of heat waves*		0.693				0.436	0.521
*Impacts of climate change—Increased rates of extinction of plant and animal species*		0.710				0.438	0.540
*Impacts of climate change—Increased global sea level*		0.577				0.317	0.351
*Impacts of climate change—Increased intensity of storms across many regions*		0.690				0.425	0.515
*Impacts of climate change—An overall decrease in clean*, *potable water globally*		0.464				0.228	0.249
*Likelihood—Take action to reduce your personal carbon footprint*			0.476			0.313	0.331
*Likelihood—Discuss climate change with your family and friends*			0.828			0.675	0.764
*Likelihood—Discuss climate change with your peers*			0.837			0.679	0.779
*Likelihood—Take some form of political action in support of climate change policy*			0.589			0.463	0.498

**Table 4:** Factor loadings and communalities based on principal axis factor analysis with orthogonal rotation (varimax with Kaiser normalization) from post-survey item analysis (N = 914; Kaiser-Meyer-Olkin measure of sampling adequacy = 0.89; Bartlett's test of sphericity *p* < 1E-9).

Post-Survey results	Factor					Communalities	
						*Initial*	*Extraction*
	1	2	3	4	5		
*Eigenvalue*	6.55	2.63	2.07	1.48	1.13		
*% of variance*	28.47	11.44	9.00	6.44	4.90		
*Survey item*							
*How worried are you about climate change*?		0.618				0.494	0.524
*Feelings about climate change—Not Guilty to Guilty*		0.478				0.255	0.261
*Feelings about climate change—Calm to Outraged/Angry*		0.660				0.389	0.463
*Feelings about climate change—Unconcerned to Alarmed*		0.672				0.499	0.547
*Feelings about climate change—Not Afraid to Very Afraid*		0.759				0.506	0.626
*How important is the issue of climate change to you personally*?		0.553				0.506	0.531
*Feelings about climate change—Hopeless to Hopeful*					0.776	0.479	0.650
*Feelings about climate change—Discouraged to Empowered*					0.809	0.476	0.685
*Impacts of climate change—Increased temperatures globally*	0.700					0.472	0.523
*Impacts of climate change—Increased incidence and intensity of heat waves*	0.775					0.557	0.639
*Impacts of climate change—Increased rates of extinction of plant and animal species*	0.670					0.490	0.519
*Impacts of climate change—Increased global sea level*	0.702					0.460	0.519
*Impacts of climate change—Increased intensity of storms across many regions*	0.698					0.451	0.514
*Impacts of climate change—An overall decrease in clean*, *potable water globally*	0.533					0.314	0.322
*Likelihood—Take action to reduce your personal carbon footprint*			0.425			0.386	0.397
*Likelihood—Discuss climate change with your family and friends*			0.813			0.704	0.812
*Likelihood—Discuss climate change with your peers*			0.797			0.676	0.766
*Likelihood—Take some form of political action in support of climate change policy*			0.458			0.426	0.411
*Effect on desire to learn—The science of climate change*				0.506		0.314	0.327
*Effect on desire to learn—Potential solutions for mitigating the effects of climate change*				0.524		0.336	0.351
*Effect on desire to learn—Politics as it relates to climate change*				0.559		0.316	0.353
*Effect on desire to learn—Economics as it relates to climate change*				0.648		0.324	0.443
*Effect on desire to learn—Energy policies*				0.630		0.332	0.432

Survey items assessing knowledge of the anthropogenic role in climate change and the dynamics of CO_2_ accumulation in the atmosphere did not load onto any other factors (Tables 3 and 4). Further, knowledge of the human role in climate change did not load together with knowledge of CO_2_ accumulation (the ability to infer how the stock of CO_2_ in the atmosphere accumulates the flow of CO_2_ emissions less the net removal of CO_2_ as it is taken up by the biosphere and ocean). However, the deficit model of risk communication suggests greater understanding that human activity is the primary cause of climate change should lead to greater desire to act and an understanding of CO_2_ accumulation dynamics may be a predictor of preferences for strong climate action [21]. We therefore include knowledge about the human cause of climate change, denoted *‘Cause’*, and understanding of the dynamics of CO_2_ accumulation, denoted *‘Stock-flow*,*’* in our analyses.

## Gains in constructs

The EFA identifies a number of constructs capturing participants’ knowledge of climate change, their affective engagement with the issue, and their intent to take action to address the problem. Here we assess whether World Climate led to changes in these constructs.

### Methods

Two-tailed paired t-tests were used to test for statistically significant shifts in the values of constructs or selected items from the pre- to post-surveys. The magnitude of differences between the post- and pre-survey means is assessed by Cohen’s *d* [50], using the pooled standard deviation for the pre- and post-survey responses:

$$d=({\bar{C}}_{post}-{\bar{C}}_{pre})/\sqrt{(s_{Post}^{2}+s_{Pre}^{2})/2},$$ (1)

where ${\bar{C}}_{pre}$ and ${\bar{C}}_{post}$ are the mean construct values and *s*_*pre*_ and *s*_*post*_ are the survey standard deviations, respectively. Effect sizes of 0.3, 0.5, and 0.8 are generally considered small, medium, and large, respectively [50].

Preliminary data analysis raised the possibility that ceiling effects might limit the measured impact of *World Climate* because pre-survey responses for some participants lay near the maximum possible value of a given construct. We therefore test for differences in gains in each construct between those participants who began the simulation with high pre-survey values compared to those with low pre-survey values by comparing the upper and lower thirds of the distribution of all usable cases. For each subsample and construct, we test for statistically and substantively significant pre- to post-survey gains using paired t-tests and Cohen’s *d* effect sizes.

### Results and discussion

Survey results from post- to pre- *World Climate* sessions show highly statistically significant gain in the constructs capturing climate change knowledge, affect, and intent to act (Table 5), even after Bonferroni correction. Participation in *World Climate* was associated with a gain in knowledge about climate change, including *Impacts* (Cohen’s *d* effect size [ES] = 0.35; *p* < 1E-9), the human role in climate change (*Cause*, ES = 0.27; *p* < 1E-9), and CO_2_ accumulation dynamics (*Stock-flow*, ES = 0.35; *p* < 1E-9). *World Climate* was associated with highly statistically significant gains in affective responses to climate change, including *Urgency* (ES = 0.38; *p* < 1E-9) and *Hope* (ES = 0.20; *p* < 1E-9), as well as gains in participants’ intent to take action on climate change (ES = 0.28, *p* < 1E-9).

**Table 5:** Comparison of pre- and post-survey means for constructs and survey items reflecting climate change knowledge (‘*Impacts*,*’ ‘Causes’*, *‘Stock-flow’*), affect (‘*Urgency*,’ and ‘*Hope*’), and intent to take action (‘*Intent*’).

* *	*Pre-mean*	*Post-mean*	*Post-Pre*	*Pre SD*	*Post SD*	*N*	*t *	*df*	*p*^*1*^	*ES*^*2*^
*Causes*	0.74	0.85	0.11	0.44	0.36	849	-7.5	848	<1E-9***	0.27
*Impacts*	0.89	0.92	0.04	0.12	0.11	858	-9.34	857	<1E-9***	0.35
*Stock-flow*	0.33	0.5	0.17	0.47	0.5	794	-9.95	793	<1E-9***	0.35
*Urgency*	0.74	0.79	0.05	0.13	0.13	858	-13.85	857	<1E-9***	0.38
*Hope*	0.61	0.65	0.04	0.18	0.21	858	-6.24	857	<1E-9***	0.20
*Intent*	0.81	0.85	0.04	0.15	0.14	858	-10.41	857	<1E-9***	0.28

^1^After Bonferroni correction, p-values < 9.6 x 10^−6^, <9.6 x 10^−5^, and 4.8 x 10^−4^ are considered statistically significant at levels of 0.001 (^***^), 0.01 (^**^), and 0.05 (^*^), respectively.

^2^ES denotes Cohen’s *d* effect size.

Results also revealed ceiling effects: gains in the constructs were not statistically significant for participants with high pre-survey values, while those with low pre-survey values showed statistically significant gains across all constructs, with moderate (*Impacts*: ES = 0.71; *Hope*: ES = 0.61) to large effect sizes (*Urgency*: ES = 0.86; *Intent*: ES = 0.85) (Table 6). By definition, there can be only small gains for participants who were already highly knowledgeable, concerned or intending to take action on climate change. Nevertheless, the gains in the constructs were statistically significant overall, with the effect driven by the large gains among those who were less knowledgeable, less concerned, and expressed low intent to act before the workshop. These results suggest the simulation was effective for participants who began with relatively little knowledge of or engagement with climate change.

**Table 6:** Analysis of gains and effect sizes for participants who began the simulation with low (lower third) vs. high (upper third) pre-survey values of each construct.

*Construct *	*Pre-value*	*Pre-mean*	*Pre SD*	*Post mean*	*Post SD*	*t*	*df*	*p*^*1*^	*ES*^*2*^
*Impacts*	High	1	0	0.98	0.05	-3.49	109	0.001	-0.57
* *	Low	0.81	0.07	0.88	0.12	5.83	97	7.00E-08***	**0.71**
*Urgency*	High	0.88	0.05	0.87	0.08	-1.86	259	0.064	-0.15
* *	Low	0.59	0.1	0.69	0.13	14.88	300	<1E-9***	**0.86**
*Hope*	High	0.78	0.1	0.76	0.16	-1.65	349	0.101	-0.15
* *	Low	0.42	0.09	0.51	0.19	8.77	302	<1E-9***	**0.61**
*Intent*	High	0.97	0.03	0.95	0.08	-3.08	286	0.002	-0.33
* *	Low	0.61	0.09	0.72	0.16	12.44	227	<1E-9***	**0.85**

^1^After Bonferroni correction, p-values < 9.6 x 10^−6^, <9.6 x 10^−5^, and 4.8 x 10^−4^ are considered statistically significant at levels of 0.001 (***)), 0.01 (**), and 0.05 (*), respectively.

^2^ES refers to Cohen’s *d* effect size.

**Fig 5:**
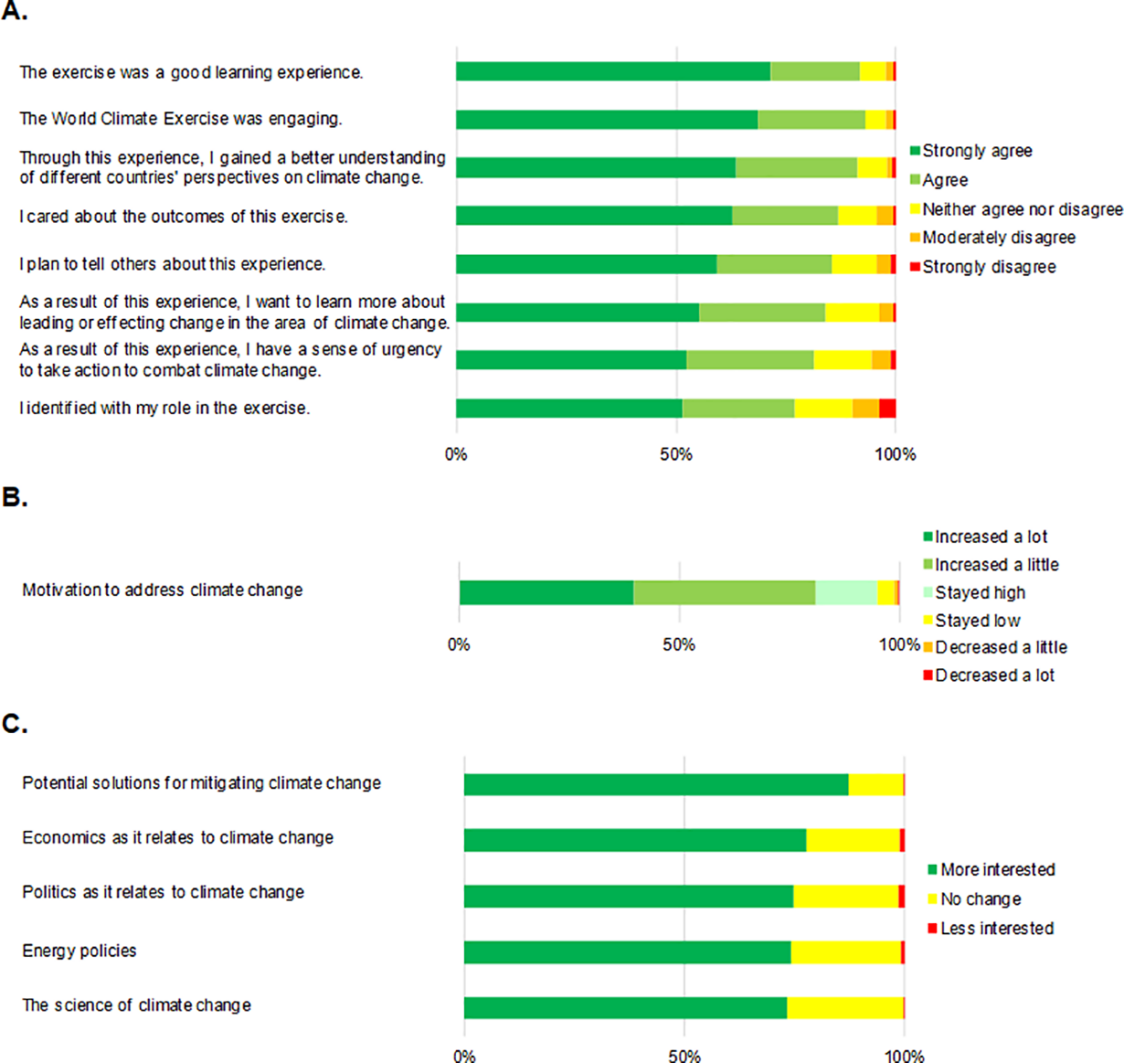
Post-survey responses to questions regarding (A) how engaging the *World Climate* simulation was as a learning experience, (B) the effects the simulation had on motivation to address climate change and (C) desire to learn more about climate change science, solutions, politics, economics, and policies; *N* ≧ 839.

Individual survey items support the results. Participants expressed increased motivation to address climate change as a result of the simulation, with 95% of post-survey respondents saying their motivation to address climate change increased a lot (40%), a little (41%), or stayed high (14%) (Fig 5). Large majorities reported that they were more interested in learning about climate change science (73%), solutions (87%), politics (76%), economics (78%), and energy policy (75%) as a result of participating (N ≧ 839).

The finding that *World Climate* is associated with substantial and statistically significant gains in understanding of accumulation (the “carbon bathtub” [21, 33]) is particularly interesting. Limiting expected warming to 2 °C requires rapid stabilization of atmospheric GHG concentrations, which, in turn, requires emissions to fall until they equal net GHG removal from the atmosphere. However, experiments show that people, including highly educated adults with substantial STEM training have difficulty understanding the dynamics of accumulation, i.e., stocks and flows [17] even in everyday contexts such as filling a bathtub or managing a bank account. In the climate context, the stock of CO_2_ in the atmosphere accumulates emissions less the net flux of CO_2_ removed from the atmosphere by terrestrial and marine sinks. Emissions are currently approximately twice as large as net removals [51], causing the concentration of CO_2_ in the atmosphere to rise. Stabilizing atmospheric CO_2_ requires emissions to fall until emissions and removal are equal. However, experiments show that many people erroneously believe that atmospheric CO_2_ can be stabilized by stabilizing emissions at or above current levels, even though emissions would then continually exceed the net removal of CO_2_ from the atmosphere—a belief that violates mass balance [33]. The first round of negotiations in many *World Climate* sessions yields global emissions that peak around 2030–2040, then fall slightly through 2100. Many participants, expecting the drop in emissions will cause a drop in atmospheric CO_2_, note that the simulation shows a continued rise (Fig 2B), motivating them to ask why and leading to discussion of the process of accumulation, often using the “carbon bathtub” analogy. The large and statistically significant gain in participant understanding of these stock-flow dynamics suggests *World Climate* is effective in building knowledge critical to understanding the conditions required to stabilize CO_2_ concentrations and global average temperatures.

## Regression analysis of associations among constructs

The information deficit model of science communication [9] suggests that gains in knowledge lead to behavior change, as represented in Fig 4 by the hypothesized links from *Knowledge* to *Intent to Act* and from *Knowledge* to *Desire to Learn*. Yet, research demonstrates the importance of affect in risk perception and action [38, 39]. Worry, interest, and hope were strongly associated with support for climate change policy in a nationally representative survey in the U.S. [39]. Similarly, Leiserowitz et al. [38] found affect to be a strong predictor of climate change risk perception. Under the information deficit model, gains in knowledge about climate change should be positively associated with gains in people’s desire to learn more about climate change and intent to take action. In contrast, under an affect-mediated model of learning, gains in the emotions people experience would be associated with gain in their desire to learn more and intent to act. Here we ask how the gains in each construct identified in the EFA are associated with gains in the others, and with a wide range of session- and participant-level attributes such as where the session was held and participant socio-demographic characteristics.

### Methods

We use multiple linear regression to assess associations among the constructs in the hypothesized learning model (Fig 4). The dependent variables are the gains, *G*, between the pre- and post-surveys for each of the constructs, *C*, identified through EFA, *G* = *C_Post_* − *C_Pre_*. For each focal construct, (e.g., the gain in *Intent*), the independent variables are the gains in the other constructs (e.g., gains in knowledge of climate change *Impacts*, *Urgency* and *Hope*). We include a variety of controls, including the pre-survey value of the constructs to test for ceiling effects that might arise in cases with high pre-survey values, and fixed effects to control for potential influence of participant or session characteristics. Controls for participants’ sociodemographic characteristics include gender, age, education, parents’ education, and perceived socioeconomic status. Session-level controls include whether the session was held in a developed or developing country, in a secondary or post-secondary educational setting, and was run by an experienced facilitator or someone with no or minimal formal training in *World Climate* (see Table 1, S1 and S2 Tables). Pearson’s correlation coefficients across session- and participant-level variables are provided in S3 Table. For all regressions, tests for collinearity and outliers included ensuring that all tolerance statistics were > 0.2, variance inflation factors (VIF) were < 10, and Cook’s distance values were <4/N.

### Results and discussion

**Fig 6:**
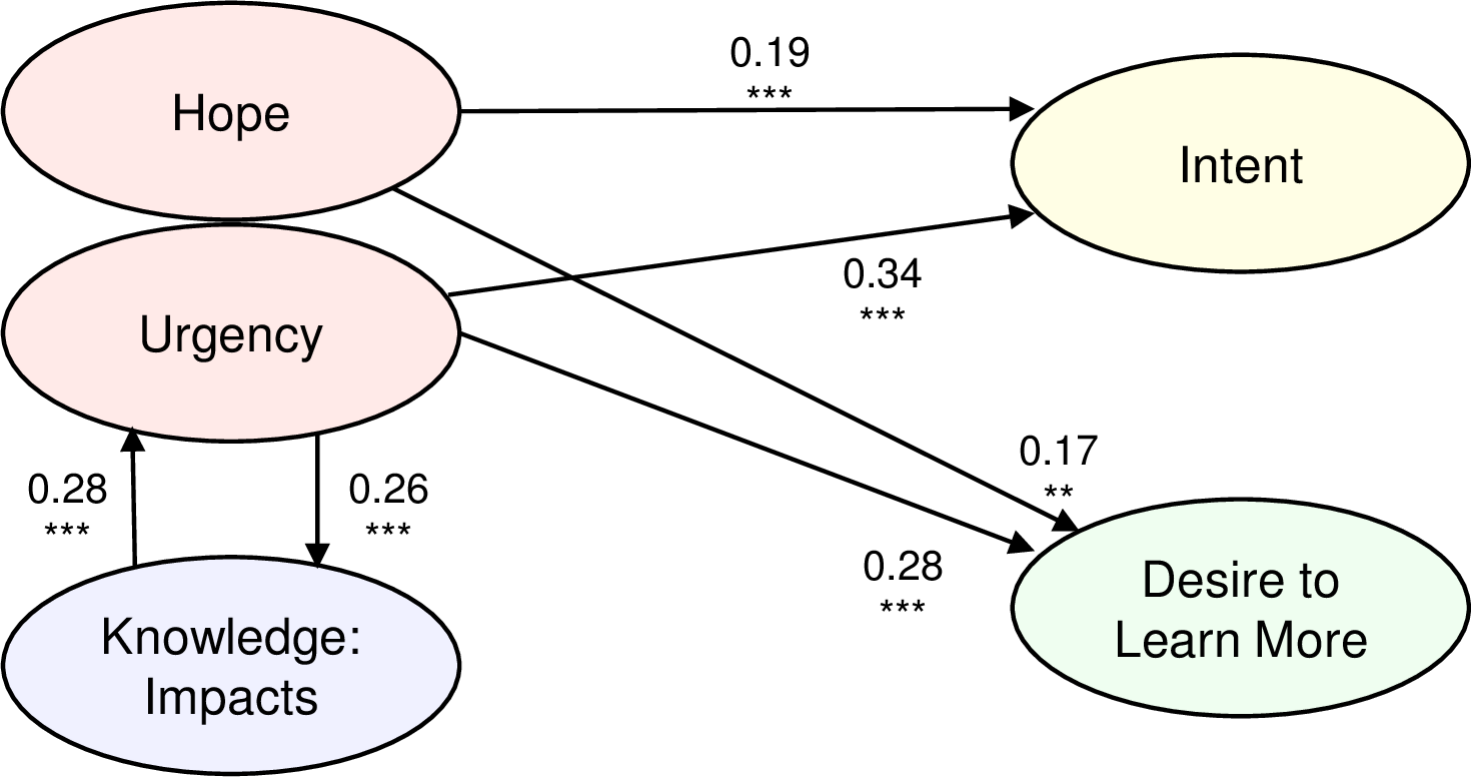
Summary of regression results, showing statistically significant relationships (arrows) among gains in constructs, including affect (*Urgency* and *Hope*), knowledge about *Impacts*, *Intent* to act and *Desire to Learn More*. Results are for Model 1 (no participant- or session-level fixed effects; values for Models 2–4 with different sets of controls are similar). Lines with arrows depict statistically significant relationships between independent and dependent variables, with standardized beta coefficients for each relationship shown. See S1 Table for detailed regression results. *** *p* < 1E-9 denotes statistical significance at α < 0.001 after Bonferroni correction. ** Beta coefficients were statistically significant at α < 0.01 after Bonferroni correction, with *p* < 1E-6.

Regression analysis revealed bidirectional associations between climate change knowledge and affect (Fig 6). A feedback is evident from gains in participants’ knowledge of climate change *Impacts* to their feeling of *Urgency* (*ß* = 0.28, *p* <1E-9) and from gains in *Urgency* back to gains in *Impacts* (*ß* = 0.26, *p* <1E-9). In contrast, learning more about the causes of climate change and the dynamics of CO_2_ accumulation has no statistically significant association with participant feelings about the urgency of addressing climate change. Further, gains in participants’ feelings of *Hope* have no association with gains in climate change knowledge and vice-versa. The regression results also show evidence for the impact of prior beliefs and affect. As expected, there are ceiling effects: participants with higher pre-survey values of each construct show smaller gains. More interesting, higher pre-survey levels of *Urgency* are associated with greater gains in knowledge of climate change after the simulation: the more worried people are before *World Climate*, the more they learn about the *Impacts* of climate change (*ß* = 0.26, *p* < 1E-9). The results support prior work on the synergies between analytic and affective processing of information about climate change [36, 37, 52]. When the two processing systems are aligned, affective engagement motivates sustained commitment to solving difficult problems [13].

In contrast to the information deficit model, our results support an affect-mediated model of learning: gains in the key constructs *Intent* and *Desire to Learn More* are not statistically significantly associated with climate change knowledge (*Cause*, *Impact*, *Stock-Flow Understanding*) (S1 and S2 Tables; Fig 6). Instead, gains in participant affect are linked to gains in *Intent* to act and *Desire to Learn More*. Specifically, gains in both *Urgency* and *Hope* are linked to gains in both *Desire to Learn More* (*ß* = 0.28, *p* <1E-9; and *ß* = 0.17, *p* = 1E-6 for *Urgency* and *Hope*, respectively) and *Intent* (*ß* = 0.34, *p* <1E-9; and *ß* = 0.19, *p* <1E-9 for *Urgency* and *Hope*, respectively) (Fig 6, S1 and S2 Tables). Similarly, higher pre-survey levels of *Urgency* are associated with larger gains in *Desire to Learn More* (*ß* = 0.36, *p* <1E-9) and *Intent* (*ß* = 0.30, *p* <1E-9). These results align with prior research: for example, anger is associated with high levels of arousal [53], while moral outrage has been identified as a ‘guardian of justice’ because it prompts social activism [54]. *Urgency* also includes participants’ degree of worry and feelings about the importance of climate change to them personally, both of which are linked to perceptions of risk, another driver of climate action [55].

In almost all cases, sociodemographic factors, session settings, and facilitator training are not associated with gains in knowledge, affect, or intent, suggesting the simulation’s versatility (S1 and S2 Tables). The exceptions were that gains in *Intent* were larger for participants who were older and gains in *Hope* were larger in sessions facilitated by the core team rather than self-taught facilitators. In other cases, the few fixed effects that attained statistical significance were inconsistent across models (e.g., the effect of age on gains in *Hope* was statistically significant only when it was considered alone, but not when multiple fixed effects were included, Table D in S1 Table).

## Effect of participant political attitudes

The effects of political ideology on climate change beliefs are well established and, in the US, free market ideology has been linked to climate change denial [12, 56, 57]. Is *World Climate* effective among those who oppose regulation and other collective action solutions for climate change?

### Methods

We test for potential effects of free-market beliefs on learning outcomes from *World Climate* in three ways. First, we compared the means for each construct (pre- and post-simulation) for participants who opposed government regulation to those who favored it, using independent samples t-tests. This analysis assesses initial and post-workshop differences in the constructs capturing climate change knowledge, affect, and intent to take action. Second, we tested whether gains in each construct were statistically significant for participants in the US who were somewhat or strongly opposed to government regulation of free markets, using paired t-tests. Third, we repeated the regression analyses of the gains in each construct described above but adding free market views as a participant-level fixed effect. Statistically significant effects of free market views, along with their sign and effect size, indicate whether free market views are associated with differences in the gains in each construct.

### Results and discussion

The results suggest *World Climate* is effective with people holding free market worldviews. Forty percent of the US participants in the sample opposed government regulation (N = 162 usable cases). Compared to participants who favored regulation, those who opposed free market regulation began the simulation with a lower belief that climate change is caused by human activities (*Cause*, *p =* 4E-6), a lower level of knowledge about CO_2_ accumulation dynamics (*Stock-flow*, *p =* 8E-5), and a lower sense of *Urgency* (*p* = 4E-6; Table 7). However, these participants experienced statistically significant gains in climate change knowledge (*Causes* [ES = 0.38, *p* = 6E-6], *Impacts* [ES = 0.34, *p* = 2E-5], *Stock-Flow Understanding* [ES = 0.41, *p* = 2E-6]), *Urgency* (ES = 0.62, *p* <1E-9), and *Intent* (ES = 0.41, *p* = 7E-7) (Table 8). These gains were large enough that those who oppose regulation showed no statistically significant differences in post-survey values of these constructs compared to those who favored government regulation (Table 7). Similarly, regression analyses indicated no association of free market views with gains in each construct (S1 and S2 Tables). Large majorities of participants who oppose regulation also reported gains in their motivation to combat climate change (81%) and found the simulation to be an engaging (92%) and effective (92%) learning experience.

**Table 7:** Comparison of construct means for US-based participants who were somewhat or strongly opposed to free market regulation compared to those somewhat or strongly in favor of regulation, before and after *World Climate*.

	*Favor Mean*	*Oppose Mean*	*Favor SD*	*Oppose SD*	*Favor N*	*Oppose N*	*T*	*df*	*p-value*
***Pre-survey values:***									
*Knowledge: Cause*	0.86	0.65	0.35	0.48	291	156	4.74	246	4E-06***
*Knowledge: Impacts*	0.91	0.88	0.13	0.12	291	157	2.72	328	0.007
*Knowledge: Stock-Flow*	0.50	0.30	0.50	0.46	269	151	4.00	332	8E-05**
*Urgency*	0.76	0.70	0.14	0.13	291	157	4.39	329	2E-05*
*Hope*	0.55	0.62	0.17	0.17	290	157	-4.18	328	4E-05**
*Intent*	0.83	0.79	0.14	0.15	290	157	2.67	292	0.008
***Post-survey values:***									
*Knowledge: Cause*	0.91	0.82	0.29	0.38	296	164	2.39	270	0.018
*Knowledge: Impacts*	0.94	0.92	0.10	0.12	295	163	2.37	289	0.019
*Knowledge: Stock-Flow*	0.64	0.52	0.48	0.50	275	157	2.51	314	0.013
*Urgency*	0.80	0.77	0.13	0.14	296	164	2.89	314	0.004
*Hope*	0.59	0.65	0.20	0.19	296	163	-3.37	349	0.001
*Intent*	0.87	0.84	0.13	0.16	295	162	1.37	274	0.171
*Desire to Learn More*	0.92	0.92	0.10	0.12	290	155	-0.04	261	0.965

**Table 8:** Comparison of pre- and post-survey results for constructs reflecting climate change affect (‘*Urgency*,’ and ‘*Hope*’), knowledge (‘*Impacts*,*’ ‘Causes*,*’ ‘Stock-Flow Understanding’*), and intent to take action (‘*Intent*’) for participants in the US who responded “somewhat opposed” or “strongly opposed” when asked, “To what extent are you in favor of the government placing regulations on the free market?”

* *	*Pre-mean*	*Post-mean*	*Post-Pre*	*SD (Pre)*	*SD (Post)*	*N*	*T*	*df*	*p-value*^*1*^	*ES*^*2*^
*Causes*	0.65	0.83	0.18	0.48	0.38	163	-4.7	162	6.00E-06***	0.42
*Impacts*	0.88	0.92	0.04	0.12	0.12	162	-4.37	161	2.00E-05**	0.33
*Stock-flow*	0.31	0.53	0.22	0.46	0.5	152	-4.97	151	2.00E-06***	0.46
*Urgency*	0.7	0.77	0.07	0.14	0.14	162	-7.86	161	<1E-9***	0.50
*Hope*	0.62	0.65	0.03	0.17	0.19	161	-2.25	160	0.03	0.17
*Intent*	0.78	0.84	0.06	0.16	0.17	160	-5.17	159	7.00E-07***	0.36

^1^ After Bonferroni correction, p-values < 9.6 x 10^−6^, <9.6 x 10^−5^, and 4.8 x 10^−4^ are considered statistically significant at levels of 0.001 (^***)^), 0.01 (^**^), and 0.05 (^*^), respectively.

^2^ES refers to Cohen’s *d* effect size.

## Threats to external validity

We now consider two potential sources of bias. First, survey completion was optional, raising the potential of bias from voluntary response sampling if participants with more extreme prior views about climate change or who had the strongest reactions to *World Climate*, positive or negative, were more likely to complete the surveys than those indifferent to the experience. Second, approximately half the participants were required to participate in *World Climate* as part of a course unrelated to climate change. However, the other half elected to participate, raising the possibility that these individuals were not representative of the populations in their nations.

### Methods

To test for voluntary response sampling bias, we (i) replicated the regression analysis, eliminating those sessions with low rates (<30%) of usable cases to assess whether inclusion of sessions with low response rates influenced results; (ii) included the session-level response rate as a regressor in the analysis to test whether variation in response rate had a statistically significant effect on the gains in each construct; (iii) compared pre-survey values of constructs and gains in constructs for sessions with high response rates to those with low response rates to test whether biases associated with response rates influenced observed gains; and (iv) compared pre-survey construct values and sociodemographic characteristics for those who completed the post-survey to those who did not to test whether there were differences among those who only provided pre-surveys and those who provided both pre- and post-surveys.

A second potential source of bias is self-selection of participants, as those choosing to participate may not be representative of broader populations. Forty-four percent of the sample participated in *World Climate* because it was a required component of a course or program unrelated to climate change, ruling out selection bias for these participants. The rest (56%) had chosen to participate in a *World Climate* session that was open to the public or had enrolled in a course in climate change or sustainability that included *World Climate* (Table 1). These participants might have been more motivated to learn about climate change or to favor climate action than the population at large. To test for self-selection bias we replicated our analysis for the participants for whom *World Climate* was a required component of their educational program.

### Results and discussion

Regression analyses excluding sessions with low survey response rates (<30%) yielded results similar to those from the full sample (S1 and S2 Tables). Comparison of results for sessions with high response rates (>50%) to those with low response rates (<50%) also showed no statistically significant differences (S4 Table). When session-level response rate was included as a regressor, it shows no statistically significant effect (S1 and S2 Tables). The results show no evidence that differences in survey response rates explain the gains in construct values after participation in *World Climate*.

With few exceptions, pre-survey responses for those who completed the post-survey reveal no statistically significant differences compared to those who chose not to complete the post-survey (S5 Table). Among sessions with high response rates (>30%), the only difference is that participants who completed both surveys were, on average, younger than those who only provided pre-surveys (S5 Table). For all sessions, those who completed both surveys show no significant differences in pre-survey values for knowledge about climate change causes and CO_2_ accumulation dynamics, *Urgency*, and *Hope*. However, they did have higher levels of education, higher pre-survey knowledge about climate change *Impacts* and *Intent* to act, and were more likely to have been in a session facilitated by our team and held at an institution of higher education (Table B in S5 Table).

Results for participants who were required to participate in *World Climate* as part of a curriculum unrelated to climate change or sustainability were similar to those who volunteered to participate, with statistically significant gains in all constructs (S6 Table). Overall, the gains in knowledge, affect, the desire to learn more and intent to take action after participating in *World Climate* are similar for those who were required to participate in the simulation compared to those who chose to participate.

## Discussion and conclusions

The *World Climate* role-play simulation offers an approach to climate change communication that enables people to learn for themselves through a scientifically grounded and socially engaging experience. Across a diverse set of participants, *World Climate* was associated with statistically significant gains in three areas: (i) knowledge of climate change causes, dynamics and impacts; (ii) affective engagement including greater feelings of urgency and hope; and (iii) a desire to learn more and intent to take action in the real world. The results are robust across diverse geographic, cultural, educational, and sociodemographic conditions, suggesting that *World Climate* is a versatile and effective tool for motivating action informed by science.

Results also suggest future work to extend the current study’s findings and address its limitations. First, although our sample includes people from eight nations and a wide range of socio-demographic backgrounds, extending the study to other nations and populations would further explore the robustness of the results. Second, all participants in the sample were asked to complete the pre-survey. We therefore cannot rule out priming effects from the pre-survey; an extension would randomly assign participants to a pre-survey or no pre-survey group. Third, although the risk communication and climate communication research shows that traditional lectures and presentations have little impact [9–14], a potential extension would be to compare *World Climate* to traditional modes of communication. Fourth, we present preliminary findings suggesting the potential for *World Climate* to reach across political divides, but this important question deserves more attention, including sampling a broader spectrum of ideological views and measuring ideological orientation more robustly. Lastly, our results measure stated intentions to learn more and to take action. Assessing the extent to which participants follow through is an important issue for future work.

The results show the importance of affective engagement in learning. In contrast with the information deficit model of communication [9], greater climate change knowledge was not directly associated with increases in people’s desire to learn more or intentions to act. Rather, increases in people’s feelings of urgency and hope—the belief that change is possible and that what individuals do can matter—were associated with gains in people’s desire to learn more about climate change science, economics and policy issues, and their intention to take action in the real world, including reducing their personal carbon footprint, talking about climate change with friends and family, and becoming more politically active. Importantly, stronger gains in feelings of urgency were also associated with larger gains in climate knowledge: Those with stronger affective engagement appear to have been motivated to learn more and show higher gains in knowledge of the causes and impacts of climate change, indicating a feedback between affect and knowledge.

Concerns that fearful messages about climate change may actually reduce risk perception and action have led to calls to avoid those messages and take a hopeful, solutions-oriented approach to climate change communication [58]. In contrast, more recent work has indicated that hopeful messages about climate change reduce risk perception, while pessimistic messages increase motivation to mitigate [59]. Similarly, we find that gains in *Urgency*, which includes participants’ degree of fear about climate change, are associated with gains in knowledge, *Intent*, and *Desire to Learn More* (Fig 4). We speculate that the positive association may arise because *World Climate* does not present participants with fearful messages that may then provoke resistance and denial, as found in prior work [20], but rather that participants feel more fearful as they experience for themselves the simulated consequences of their own decisions about global GHG emissions.

The social aspect of *World Climate* likely contributes to its impact on participants’ beliefs and emotional engagement around climate change. Social context strongly influences how information about climate change is perceived and used [20], and the social identity of the messenger influences the efficacy of climate communication, with the most effective messengers being trusted individuals who share social group membership with their audience [60]. The role of messenger in *World Climate* is fulfilled primarily by the participants themselves. Participants spend most of the simulation time engaged in discussion with one another (Fig 3), not passively receiving information presented by an authority, and 92% of the participants in our sample report that *World Climate* is an engaging experience (Fig 5). The results support calls to view climate change communication as an interactive dialogue, rather than information transmission from experts to the public [9]. The results also suggest the potential of the simulation to reach across political divides, at least among participants in the US. Participants in sessions run in the US who oppose government regulation of the free market showed gains in knowledge, affect, desire to learn and intent to take action at least as large as the gains among others, a finding that is particularly important given the polarization of US public opinion about climate change [6].

*World Climate* is designed to be easily and broadly adopted. All materials are freely available, including facilitation guides and videos, participant materials, facilitator slide decks, and the C-ROADS computer model. More than 42,000 people in 77 countries participated in *World Climate* between August 2015 and May 2018. *World Climate* has been externally reviewed by educators and scientists [61], found to support the US Next Generation Science Standards [62], and has been designated as an official resource for schools in Germany (Beule, personal communication), France [28] and South Korea [29]. We conclude that simulations like *World Climate* may offer a scalable means to catalyze climate action that is informed by science.

## Supporting information

S1 Table Estimates from linear regressions with all sessions included in the analysis. After Bonferroni correction, p-values < 9.6 x 10^−6^, <9.6 x 10^−5^, and 4.8 x 10^−4^ are considered significant at levels of 0.001 (***)), 0.01 (**), and 0.05 (*), respectively.
(DOCX)

S2 Table Regression results with data limited to sessions with >30% response rates. After Bonferroni correction, p-values < 9.6 x 10^−6^, <9.6 x 10^−5^, and 4.8 x 10^−4^ are considered significant at α levels of 0.001 (***)), 0.01 (**), and 0.05 (*) respectively.
(DOCX)

S3 Table **Correlation matrices for session-level (A) and participant-level control variables (B).** Pearson correlation coefficients are provided, with bold text reflecting correlations that are statistically significant at *p* < 0.05.
(DOCX)

S4 Table Comparison of gains in constructs from sessions with higher-than-median number of usable survey responses (Hi-part) to those with lower-than-median number of usable cases (Lo-part). (DOCX)

S5 Table Comparison of pre-survey responses for participants who completed the post-survey (i.e., >80% of items in both pre- and post-surveys provided) to those who did not. (DOCX)

S6 Table Comparison of pre- and post-survey means and paired t-tests for constructs reflecting climate change affect (‘Urgency,’ and ‘Hope’), knowledge (‘Impacts,’ ‘Causes’, ‘Stock-flow task’), and intent to take action (‘Intent’) among participants who were required to participate in *World Climate* as part of a program unrelated to climate change, environment or sustainability, ruling out self-selection bias. (DOCX)

S1 Appendix Briefing memos provided to participants during *World Climate*. (PDF)

S2 Appendix Pre- and post-surveys. (DOCX)
