# Supplementary material for: Combining role-play with interactive simulation to motivate informed climate action: Evidence from the World Climate simulation
Source: PLoS One. 2018 Aug 30;13(8):e0202877. doi: 10.1371/journal.pone.0202877 (PMC6117006; doi:10.1371/journal.pone.0202877)
Supplement: S1 Table — After Bonferroni correction, p-values < 9.6 x 10^−6^, <9.6 x 10^−5^, and 4.8 x 10^−4^ are considered significant at levels of 0.001 (***)), 0.01 (**), and 0.05 (*), respectively. (DOCX) [file pone.0202877.s001.docx]

1. Regression results for Urgency gains.

|  | **Model 1:**  **Base model** | | **Model 2:**  **Session-level**  **fixed effects** | | **Model 3:**  **Gender/**  **Age** | | **Model 4:**  **Other**  **demographic**  **info** | | **Model 5:**  **All fixed**  **effects** | |
| --- | --- | --- | --- | --- | --- | --- | --- | --- | --- | --- |
| **Parameter:** | *Beta* | *p* | *Beta* | *p* | *Beta* | *p* | *Beta* | *p* | *Beta* | *p* |
| Gain in Knowledge: Causes | 0.078 | 0.053 | 0.078 | 0.053 | 0.080 | 0.048 | 0.077 | 0.057 | 0.075 | 0.063 |
| Gain in Knowledge: Impacts | 0.279 | <1E-9^***^ | 0.280 | <1E-9^***^ | 0.272 | <1E-9^***^ | 0.269 | <1E-9^***^ | 0.271 | <1E-9^***^ |
| Gain in Knowledge: Stock-Flow | 0.030 | 0.397 | 0.028 | 0.445 | 0.012 | 0.742 | 0.021 | 0.571 | 0.014 | 0.705 |
| Pre-Knowledge: Causes | 0.027 | 0.527 | 0.024 | 0.573 | 0.036 | 0.398 | 0.020 | 0.645 | 0.025 | 0.558 |
| Pre-Knowledge: Impacts | 0.190 | 5E-06^***^ | 0.190 | 6E-06^***^ | 0.191 | 5E-06^***^ | 0.182 | 2E-05^**^ | 0.193 | 6E-06^***^ |
| Pre-Knowledge: Stock-Flow | 0.033 | 0.350 | 0.030 | 0.433 | 0.008 | 0.832 | 0.010 | 0.801 | 0.002 | 0.956 |
| Pre-Urgency | -0.489 | <1E-9^***^ | -0.484 | <1E-9^***^ | -0.500 | <1E-9^***^ | -0.490 | <1E-9^***^ | -0.496 | <1E-9^***^ |
| Percent usable cases |  |  | 0.011 | 0.773 |  |  |  |  | 0.036 | 0.354 |
| Facilitated by core team |  |  | -0.003 | 0.942 |  |  |  |  | -0.030 | 0.451 |
| Setting (Higher Ed or Secondary) |  |  | 0.002 | 0.951 |  |  |  |  | -0.087 | 0.092 |
| Country type (Developed or Developing) |  |  | 0.028 | 0.439 |  |  |  |  | 0.007 | 0.863 |
| Gender |  |  |  |  | -0.092 | 0.003 |  |  | -0.088 | 0.005 |
| Age |  |  |  |  | 0.080 | 0.013 |  |  | 0.096 | 0.081 |
| Education of Parents |  |  |  |  |  |  | -0.004 | 0.893 | 0.006 | 0.854 |
| Education of Self |  |  |  |  |  |  | 0.043 | 0.238 | 0.036 | 0.619 |
| Science Major |  |  |  |  |  |  | -0.012 | 0.713 | 0.011 | 0.761 |
| Perceived socioeconomic status |  |  |  |  |  |  | -0.052 | 0.131 | -0.046 | 0.199 |
| Favor regulation of free market |  |  |  |  |  |  | 0.007 | 0.825 | 0.011 | 0.749 |
|  |  |  |  |  |  |  |  |  |  |  |
| R^2^ | 0.261 |  | 0.262 |  | 0.275 |  | 0.266 |  | 0.281 |  |
| ANOVA F | 39.39 |  | 25.05 |  | 32.17 |  | 22.82 |  | 16.27 |  |
| p-value | <1E-9^***^ |  | <1E-9^***^ |  | <1E-9^***^ |  | <1E-9^***^ |  | <1E-9^***^ |  |
| df regression | 7 |  | 11 |  | 9 |  | 12 |  | 18 |  |
| df residual | 779 |  | 775 |  | 764 |  | 754 |  | 748 |  |
| df Total | 786 |  | 786 |  | 773 |  | 766 |  | 766 |  |
| N | 787 |  | 787 |  | 774 |  | 767 |  | 767 |  |

1. Regression results for Hope gains.

|  | ***Model 1:***  ***Base model*** | | ***Model 2:***  ***Session-level***  ***fixed effects*** | | ***Model 3:***  ***Gender/***  ***Age*** | | ***Model 4:***  ***Other***  ***demographic***  ***info*** | | ***Model 5:***  ***All fixed***  ***effects*** | |
| --- | --- | --- | --- | --- | --- | --- | --- | --- | --- | --- |
| ***Parameter:*** | Beta | *p* | Beta | *p* | Beta | *p* | Beta | *p* | Beta | *p* |
| *Gain in Knowledge: Causes* | 0.040 | 0.364 | 0.045 | 0.287 | 0.039 | 0.374 | 0.042 | 0.342 | 0.049 | 0.249 |
| *Gain in Knowledge: Impacts* | 0.057 | 0.160 | 0.031 | 0.430 | 0.041 | 0.318 | 0.041 | 0.320 | 0.038 | 0.348 |
| *Gain in Knowledge: Stock-Flow* | -0.058 | 0.133 | -0.077 | 0.043 | -0.079 | 0.043 | -0.075 | 0.058 | -0.073 | 0.058 |
| *Pre-Knowledge: Causes* | -0.067 | 0.145 | -0.050 | 0.260 | -0.064 | 0.165 | -0.049 | 0.286 | -0.034 | 0.456 |
| *Pre-Knowledge: Impacts* | 0.029 | 0.499 | -0.009 | 0.829 | 0.014 | 0.749 | 0.010 | 0.826 | 0.000 | 0.991 |
| *Pre-Knowledge: Stock-Flow* | -0.044 | 0.262 | -0.064 | 0.110 | -0.078 | 0.052 | -0.054 | 0.196 | -0.044 | 0.288 |
| *Pre-Hope* | -0.329 | <1E-9^***^ | -0.383 | <1E-9^***^ | -0.336 | <1E-9^***^ | -0.370 | <1E-9^***^ | -0.398 | <1E-9^***^ |
| *Percent usable cases* |  |  | -0.060 | 0.115 |  |  |  |  | -0.055 | 0.173 |
| *Facilitated by core team* |  |  | 0.188 | <1E-9^***^ |  |  |  |  | 0.180 | 2E-05^**^ |
| *Setting (Higher Ed or Secondary)* |  |  | 0.092 | 0.011 |  |  |  |  | 0.153 | 0.005 |
| *Country type (Developed or Developing)* |  |  | -0.116 | 0.003 |  |  |  |  | -0.117 | 0.006 |
| *Gender* |  |  |  |  | 0.005 | 0.892 |  |  | -0.005 | 0.886 |
| *Age* |  |  |  |  | 0.129 | 3E-04^**^ |  |  | 0.123 | 0.035 |
| *Education of Parents* |  |  |  |  |  |  | -0.050 | 0.166 | -0.020 | 0.580 |
| *Education of Self* |  |  |  |  |  |  | 0.115 | 0.004 | -0.161 | 0.035 |
| *Science Major* |  |  |  |  |  |  | 0.059 | 0.113 | -0.002 | 0.949 |
| *Perceived socioeconomic status* |  |  |  |  |  |  | 0.081 | 0.032 | 0.045 | 0.231 |
| *Favor regulation of free market* |  |  |  |  |  |  | -0.060 | 0.092 | -0.028 | 0.429 |
|  |  |  |  |  |  |  |  |  |  |  |
| *R^2^* | 0.115 |  | 0.184 |  | 0.131 |  | 0.143 |  | 0.195 |  |
| *ANOVA F* | 14.48 |  | 15.89 |  | 12.75 |  | 10.51 |  | 10.07 |  |
| *p-value* | <1E-9^***^ |  | <1E-9^***^ |  | <1E-9^***^ |  | <1E-9^***^ |  | <1E-9^***^ |  |
| *df Regression* | 7 |  | 11 |  | 9 |  | 12 |  | 18 |  |
| *df Residual* | 779 |  | 775 |  | 764 |  | 754 |  | 748 |  |
| *df Total* | 786 |  | 786 |  | 773 |  | 766 |  | 766 |  |
| *N* | 787 |  | 787 |  | 774 |  | 767 |  | 767 |  |

1. Regression results for gains in knowledge about impacts.

|  | **Model 1:**  **Base model** | | **Model 2:**  **Session-level**  **fixed effects** | | **Model 3:**  **Gender/**  **Age** | | **Model 4:**  **Other**  **demographic**  **info** | | **Model 5:**  **All fixed**  **effects** | |
| --- | --- | --- | --- | --- | --- | --- | --- | --- | --- | --- |
| **Parameter:** | Beta | *p* | Beta | *p* | Beta | *p* | Beta | *p* | Beta | *p* |
| Gain in Affect: Urgency | 0.258 | <1E-9^***^ | 0.251 | <1E-9^***^ | 0.250 | <1E-9^***^ | 0.240 | <1E-9^***^ | 0.245 | <1E-9^***^ |
| Gain in Affect: Hope | 0.033 | 0.257 | 0.019 | 0.536 | 0.022 | 0.454 | 0.025 | 0.405 | 0.027 | 0.398 |
| Pre-Knowledge: Impacts | -0.586 | <1E-9^***^ | -0.600 | <1E-9^***^ | -0.594 | <1E-9^***^ | -0.604 | <1E-9^***^ | -0.609 | <1E-9^***^ |
| Pre-Urgency | 0.250 | <1E-9^***^ | 0.245 | <1E-9^***^ | 0.242 | <1E-9^***^ | 0.235 | <1E-9^***^ | 0.245 | <1E-9^***^ |
| Pre-Hope | -0.003 | 0.917 | -0.009 | 0.773 | -0.012 | 0.680 | -0.005 | 0.874 | -0.002 | 0.949 |
| Percent usable cases |  |  | -0.010 | 0.756 |  |  |  |  | 0.000 | 0.992 |
| Facilitated by core team |  |  | 0.030 | 0.370 |  |  |  |  | 0.018 | 0.614 |
| Setting (Higher Ed or Secondary) |  |  | 0.127 | 1E-05^**^ |  |  |  |  | 0.057 | 0.228 |
| Country type (Developed or Developing) |  |  | 0.038 | 0.240 |  |  |  |  | 0.041 | 0.264 |
| Gender |  |  |  |  | 0.028 | 0.324 |  |  | 0.026 | 0.373 |
| Age |  |  |  |  | 0.083 | 0.004 |  |  | -0.033 | 0.508 |
| Education of Parents |  |  |  |  |  |  | 0.043 | 0.155 | 0.037 | 0.236 |
| Education of Self |  |  |  |  |  |  | 0.094 | 0.004 | 0.083 | 0.206 |
| Science Major |  |  |  |  |  |  | 0.039 | 0.211 | 0.040 | 0.214 |
| Perceived socioeconomic status |  |  |  |  |  |  | -0.031 | 0.320 | -0.028 | 0.381 |
| Favor regulation of free market |  |  |  |  |  |  | 0.000 | 0.991 | -0.008 | 0.801 |
|  |  |  |  |  |  |  |  |  |  |  |
| R^2^ | 0.35 |  | 0.36 |  | 0.35 |  | 0.36 |  | 0.37 |  |
| ANOVA F | 89.90 |  | 53.14 |  | 64.62 |  | 45.63 |  | 28.68 |  |
| p-value | <1E-9^***^ |  | <1E-9^***^ |  | <1E-9^***^ |  | <1E-9^***^ |  | <1E-9^***^ |  |
| df regression | 5 |  | 9 |  | 7 |  | 10 |  | 16 |  |
| df residual | 852 |  | 848 |  | 829 |  | 796 |  | 790 |  |
| df Total | 857 |  | 857 |  | 836 |  | 806 |  | 806 |  |
| N | 858 |  | 858 |  | 837 |  | 807 |  | 807 |  |

1. Regression results for intent to take action.

|  | ***Model 1:***  ***Base model*** | | ***Model 2:***  ***Session-level***  ***fixed effects*** | | ***Model 3:***  ***Gender***  ***Age*** | | ***Model 4:***  ***Other***  ***demographic***  ***info*** | | ***Model 5:***  ***All fixed***  ***effects*** | |
| --- | --- | --- | --- | --- | --- | --- | --- | --- | --- | --- |
| ***Parameter:*** | Beta | p | Beta | *p* | Beta | *p* | Beta | *p* | Beta | *p* |
| *Gain in Knowledge: Causes* | 0.006 | 0.867 | 0.011 | 0.766 | 0.018 | 0.601 | 0.028 | 0.448 | 0.039 | 0.284 |
| *Gain in Knowledge: Impacts* | 0.114 | 0.001 | 0.100 | 0.005 | 0.101 | 0.004 | 0.118 | 0.001 | 0.119 | 0.001 |
| *Gain in Knowledge: Stock-Flow* | 0.087 | 0.006 | 0.061 | 0.060 | 0.053 | 0.097 | 0.064 | 0.055 | 0.056 | 0.098 |
| *Gain in Urgency* | 0.344 | <1E-9^***^ | 0.343 | <1E-9^***^ | 0.324 | <1E-9^***^ | 0.341 | <1E-9^***^ | 0.322 | <1E-9^***^ |
| *Gain in Hope* | 0.186 | <1E-9 | 0.176 | 9E-09^***^ | 0.188 | <1E-9^***^ | 0.184 | 4E-09^***^ | 0.199 | <1E-9^***^ |
| *Pre-Knowledge: Causes* | -0.036 | 0.343 | -0.038 | 0.319 | -0.040 | 0.286 | -0.018 | 0.652 | -0.016 | 0.676 |
| *Pre-Knowledge: Impacts* | 0.084 | 0.029 | 0.075 | 0.048 | 0.086 | 0.022 | 0.106 | 0.009 | 0.112 | 0.005 |
| *Pre-Knowledge: Stock-Flow* | 0.034 | 0.292 | -0.005 | 0.873 | -0.011 | 0.747 | 0.012 | 0.741 | -0.003 | 0.938 |
| *Pre-Urgency* | 0.295 | <1E-9^***^ | 0.307 | <1E-9^***^ | 0.298 | <1E-9^***^ | 0.291 | <1E-9^***^ | 0.290 | <1E-9^***^ |
| *Pre-Hope* | 0.160 | 2E-07^***^ | 0.164 | 3E-07^***^ | 0.158 | 2E-07^***^ | 0.162 | 1E-06^***^ | 0.171 | 4E-07^***^ |
| *Pre-Intent* | -0.618 | <1E-9^***^ | -0.622 | <1E-9^***^ | -0.664 | <1E-9^***^ | -0.661 | <1E-9^***^ | -0.673 | <1E-9^***^ |
| *Percent usable cases* |  |  | 0.034 | 0.291 |  |  |  |  | 0.018 | 0.601 |
| *Facilitated by core team* |  |  | 0.031 | 0.362 |  |  |  |  | -0.001 | 0.978 |
| *Setting (Higher Ed or Secondary)* |  |  | 0.112 | 1E-05^**^ |  |  |  |  | 0.024 | 0.571 |
| *Country type (Developed or Developing)* |  |  | 0.068 | 0.039 |  |  |  |  | 0.030 | 0.387 |
| *Gender* |  |  |  |  | 0.004 | 0.891 |  |  | 0.001 | 0.962 |
| *Age* |  |  |  |  | 0.157 | <1E-9^***^ |  |  | 0.206 | 3E-05^**^ |
| *Education of Parents* |  |  |  |  |  |  | 0.029 | 0.337 | 0.054 | 0.077 |
| *Education of Self* |  |  |  |  |  |  | 0.100 | 0.003 | -0.078 | 0.174 |
| *Science Major* |  |  |  |  |  |  | 0.024 | 0.452 | 0.026 | 0.421 |
| *Perceived socioeconomic status* |  |  |  |  |  |  | 0.018 | 0.579 | 0.040 | 0.215 |
| *Favor regulation of free market* |  |  |  |  |  |  | -0.025 | 0.409 | -0.014 | 0.644 |
|  |  |  |  |  |  |  |  |  |  |  |
| *R^2^* | 0.41 |  | 0.42 |  | 0.44 |  | 0.43 |  | 0.46 |  |
| *ANOVA F* | 48.65 |  | 37.75 |  | 45.91 |  | 33.16 |  | 26.05 |  |
| *p-value* | <1E-9^***^ |  | <1E-9^***^ |  | <1E-9^***^ |  | <1E-9^***^ |  | <1E-9^***^ |  |
| *df Regression* | 11 |  | 15 |  | 10 |  | 16 |  | 22 |  |
| *df Residual* | 775 |  | 771 |  | 13 |  | 701 |  | 686 |  |
| *df Total* | 786 |  | 786 |  | 753 |  | 717 |  | 708 |  |
| *N* | 787 |  | 787 |  | 766 |  | 718 |  | 709 |  |

1. Regression results for desire to learn more.

|  | ***Model 1:***  ***Base model*** | | ***Model 2:***  ***Session-level***  ***fixed effects*** | | ***Model 3:***  ***Gender***  ***Age*** | | ***Model 4:***  ***Other***  ***demographic***  ***info*** | | ***Model 5:***  ***All fixed***  ***effects*** | |
| --- | --- | --- | --- | --- | --- | --- | --- | --- | --- | --- |
| ***Parameter:*** | Beta | p | Beta | *p* | Beta | *p* | Beta | *p* | Beta | *p* |
| *Gain in Urgency* | 0.275 | <1E-9^***^ | 0.277 | <1E-9^***^ | 0.263 | <1E-9^***^ | 0.265 | <1E-9 | 0.275 | <1E-9^***^ |
| *Gain in Hope* | 0.170 | 1E-06^***^ | 0.106 | 0.003 | 0.144 | 4E-05^**^ | 0.138 | 8E-05 | 0.100 | 0.005 |
| *Gain in Knowledge: Impacts* | 0.090 | 0.030 | 0.060 | 0.136 | 0.071 | 0.082 | 0.061 | 0.138 | 0.057 | 0.162 |
| *Gain in Knowledge: Causes* | -0.046 | 0.287 | -0.034 | 0.413 | -0.045 | 0.283 | -0.046 | 0.279 | -0.038 | 0.364 |
| *Gain in Knowledge: Stock-Flow* | 0.061 | 0.108 | 0.022 | 0.564 | 0.030 | 0.427 | 0.018 | 0.635 | 0.016 | 0.664 |
| *Pre-Urgency* | 0.355 | <1E-9^***^ | 0.334 | <1E-9^***^ | 0.341 | <1E-9^***^ | 0.331 | <1E-9 | 0.329 | <1E-9^***^ |
| *Pre-Hope* | 0.164 | 4E-06^***^ | 0.101 | 0.005 | 0.140 | 7E-05^**^ | 0.122 | 0.001 | 0.088 | 0.019 |
| *Pre-Knowledge: Impacts* | 0.081 | 0.070 | 0.050 | 0.252 | 0.061 | 0.168 | 0.052 | 0.249 | 0.047 | 0.290 |
| *Pre-Knowledge: Causes* | -0.046 | 0.309 | -0.033 | 0.450 | -0.044 | 0.320 | -0.043 | 0.344 | -0.033 | 0.466 |
| *Pre-Knowledge: Stock-Flow* | 0.027 | 0.473 | -0.024 | 0.543 | -0.023 | 0.550 | -0.037 | 0.350 | -0.032 | 0.432 |
| *Percent usable cases* |  |  | -0.021 | 0.574 |  |  |  |  | 0.011 | 0.778 |
| *Facilitated by core team* |  |  | 0.100 | 0.011 |  |  |  |  | 0.068 | 0.102 |
| *Setting (Higher Ed or Secondary)* |  |  | 0.193 | 5E-08^***^ |  |  |  |  | 0.159 | 0.003 |
| *Country type (Developed or Developing)* |  |  | -0.074 | 0.054 |  |  |  |  | -0.086 | 0.039 |
| *Gender* |  |  |  |  | 0.060 | 0.067 |  |  | 0.049 | 0.129 |
| *Age* |  |  |  |  | 0.191 | 3E-08^***^ |  |  | 0.152 | 0.008 |
| *Education of Parents* |  |  |  |  |  |  | 0.013 | 0.713 | 0.036 | 0.304 |
| *Education of Self* |  |  |  |  |  |  | 0.180 | 3E-06^***^ | -0.093 | 0.209 |
| *Science Major* |  |  |  |  |  |  | 0.097 | 0.006 | 0.060 | 0.104 |
| *Perceived socioeconomic status* |  |  |  |  |  |  | 0.046 | 0.207 | 0.029 | 0.422 |
| *Favor regulation of free market* |  |  |  |  |  |  | -0.005 | 0.885 | 0.017 | 0.618 |
|  |  |  |  |  |  |  |  |  |  |  |
| *R^2^* | 0.17 |  | 0.23 |  | 0.21 |  | 0.22 |  | 0.24 |  |
| *ANOVA F* | 16.01 |  | 16.34 |  | 16.72 |  | 13.74 |  | 11.39 |  |
| *p-value* | <1E-9^***^ |  | <1E-9^***^ |  | <1E-9^***^ |  | <1E-9^***^ |  | <1E-9^***^ |  |
| *df Regression* | 10 |  | 14 |  | 12 |  | 15 |  | 21 |  |
| *df Residual* | 776 |  | 772 |  | 761 |  | 751 |  | 745 |  |
| *df Total* | 786 |  | 786 |  | 773 |  | 766 |  | 766 |  |
| *N* | 787 |  | 787 |  | 774 |  | 767 |  | 767 |  |
