# Supplementary material for: Combining role-play with interactive simulation to motivate informed climate action: Evidence from the World Climate simulation
Source: PLoS One. 2018 Aug 30;13(8):e0202877. doi: 10.1371/journal.pone.0202877 (PMC6117006; doi:10.1371/journal.pone.0202877)
Supplement: S2 Table — After Bonferroni correction, p-values < 9.6 x 10^−6^, <9.6 x 10^−5^, and 4.8 x 10^−4^ are considered significant at α levels of 0.001 (***)), 0.01 (**), and 0.05 (*) respectively. (DOCX) [file pone.0202877.s002.docx]

1. Regression results for gains in Urgency:

|  | ***Model 1:***  ***Base model*** | | ***Model 2:***  ***Session-level***  ***fixed effects*** | | ***Model 3:***  ***Gender***  ***Age*** | | ***Model 4:***  ***Other***  ***demographic***  ***info*** | | ***Model 5:***  ***All fixed***  ***effects*** | |
| --- | --- | --- | --- | --- | --- | --- | --- | --- | --- | --- |
| ***Parameter:*** | Beta | *p* | Beta | *p* | Beta | *p* | Beta | *p* | Beta | p |
| *Gain in Knowledge: Causes* | 0.131 | 0.009 | 0.125 | 0.013 | 0.134 | 0.007 | 0.126 | 0.015 | 0.127 | 0.014 |
| *Gain in Knowledge: Impacts* | 0.230 | 2E-06^***^ | 0.231 | 2E-06^***^ | 0.220 | 8E-01 | 0.215 | 1E-05^**^ | 0.227 | 5E-6^***^ |
| *Gain in Knowledge: Stock-Flow* | 0.023 | 0.609 | 0.018 | 0.689 | 0.008 | 0.850 | 0.010 | 0.833 | 0.004 | 0.932 |
| *Pre-Knowledge: Causes* | 0.106 | 0.054 | 0.103 | 0.061 | 0.116 | 0.033 | 0.102 | 0.069 | 0.105 | 0.061 |
| *Pre-Knowledge: Impacts* | 0.182 | 0.001 | 0.185 | 0.001 | 0.176 | 0.001 | 0.172 | 0.002 | 0.193 | 0.001 |
| *Pre-Knowledge: Stock-Flow* | 0.041 | 0.367 | 0.033 | 0.493 | 0.019 | 0.683 | 0.011 | 0.827 | 0.007 | 0.883 |
| *Pre-Urgency* | -0.538 | <1E-9^***^ | -0.549 | <1E-9^***^ | -0.550 | <1E-9^***^ | -0.539 | <1E-9^***^ | -0.565 | <1E-9^***^ |
| *Percent usable cases* |  |  | -0.065 | 0.104 |  |  | 0.000 | 0.997 | -0.029 | 0.518 |
| *Facilitated by core team* |  |  | -0.042 | 0.293 |  |  | 0.031 | 0.495 | -0.068 | 0.102 |
| *Setting (Higher Ed or Secondary)* |  |  | 0.005 | 0.911 |  |  | 0.030 | 0.477 | -0.158 | 0.074 |
| *Country type (Developed or Developing)* |  |  | 0.015 | 0.719 |  |  | -0.06 | 0.140 | -0.004 | 0.936 |
| *Gender* |  |  |  |  | -0.100 | 0.010 | 0.00 | 0.960 | -0.102 | 0.010 |
| *Age* |  |  |  |  | 0.092 | 0.022 |  |  | 0.095 | 0.194 |
| *Education of Parents* |  |  |  |  |  |  | 0.00 | 0.997 | 0.017 | 0.697 |
| *Education of Self* |  |  |  |  |  |  | 0.03 | 0.495 | 0.087 | 0.389 |
| *Science Major* |  |  |  |  |  |  | 0.030 | 0.477 | 0.045 | 0.302 |
| *Perceived socioeconomic status* |  |  |  |  |  |  | -0.064 | 0.139 | -0.059 | 0.187 |
| *Favor regulation of free market* |  |  |  |  |  |  | 0.002 | 0.959 | 0.006 | 0.888 |
|  |  |  |  |  |  |  |  |  |  |  |
| *R^2^* | 0.27 |  | 0.28 |  | 0.29 |  | 0.28 |  | 0.30 |  |
| *ANOVA F* | 26.09 |  | 16.97 |  | 21.95 |  | 15.06 |  | 11.08 |  |
| *p-value* | <1E-9^***^ |  | <1E-9^***^ |  | <1E-9^***^ |  | <1E-9^***^ |  | <1E-9^***^ |  |
| *df regression* | 7 |  | 11 |  | 9 |  | 12 |  | 18 |  |
| *df residual* | 495 |  | 491 |  | 493 |  | 473 |  | 467 |  |
| *df Total* | 502 |  | 502 |  | 502 |  | 485 |  | 485 |  |
| *N* | 503 |  | 503 |  | 503 |  | 486 |  | 486 |  |

1. Regression results for gains in Hope:

|  | ***Model 1:***  ***Base model*** | | ***Model 2:***  ***Session-level***  ***fixed effects*** | | ***Model 3:***  ***Gender/***  ***Age*** | | ***Model 4:***  ***Other***  ***demographic***  ***info*** | | ***Model 5:***  ***All fixed***  ***effects*** | |
| --- | --- | --- | --- | --- | --- | --- | --- | --- | --- | --- |
| ***Parameter:*** | Beta | *p* | Beta | *p* | Beta | *p* | Beta | *p* | Beta | *p* |
| *Gain in Knowledge: Causes* | -0.026 | 0.629 | -0.038 | 0.465 | -0.026 | 0.623 | -0.024 | 0.653 | -0.031 | 0.558 |
| *Gain in Knowledge: Impacts* | 0.078 | 0.125 | 0.022 | 0.661 | 0.061 | 0.231 | 0.041 | 0.428 | 0.021 | 0.685 |
| *Gain in Knowledge: Stock-Flow* | -0.085 | 0.077 | -0.113 | 0.017 | -0.105 | 0.030 | -0.117 | 0.017 | -0.107 | 0.028 |
| *Pre-Knowledge: Causes* | -0.104 | 0.070 | -0.106 | 0.056 | -0.094 | 0.098 | -0.095 | 0.099 | -0.106 | 0.063 |
| *Pre-Knowledge: Impacts* | 0.098 | 0.071 | 0.033 | 0.532 | 0.080 | 0.141 | 0.065 | 0.232 | 0.039 | 0.470 |
| *Pre-Knowledge: Stock-Flow* | -0.008 | 0.873 | -0.054 | 0.272 | -0.043 | 0.387 | -0.072 | 0.165 | -0.053 | 0.314 |
| *Pre-Hope* | -0.376 | <1E-9^***^ | -0.410 | <1E-9^***^ | -0.379 | <1E-9^***^ | -0.413 | <1E-9^***^ | -0.421 | <1E-9^***^ |
| *Percent usable cases* |  |  | -0.095 | 0.022 |  |  |  |  | -0.106 | 0.026 |
| *Facilitated by core team* |  |  | 0.087 | 0.035 |  |  |  |  | 0.089 | 0.040 |
| *Setting (Higher Ed or Secondary)* |  |  | 0.172 | 1E-04 |  |  |  |  | 0.174 | 0.062 |
| *Country type (Developed or Developing)* |  |  | -0.123 | 0.004 |  |  |  |  | -0.107 | 0.024 |
| *Gender* |  |  |  |  | -0.039 | 0.346 |  |  | -0.058 | 0.166 |
| *Age* |  |  |  |  | 0.130 | 0.003 |  |  | -0.067 | 0.381 |
| *Education of Parents* |  |  |  |  |  |  | -0.061 | 0.174 | -0.079 | 0.084 |
| *Education of Self* |  |  |  |  |  |  | 0.192 | 1E-04 | 0.060 | 0.574 |
| *Science Major* |  |  |  |  |  |  | 0.081 | 0.071 | 0.036 | 0.439 |
| *Perceived socioeconomic status* |  |  |  |  |  |  | 0.00 | 0.955 | -0.017 | 0.710 |
| *Favor regulation of free market* |  |  |  |  |  |  | 0.00 | 0.981 | 0.003 | 0.938 |
|  |  |  |  |  |  |  |  |  |  |  |
| *R^2^* | 0.16 |  | 0.22 |  | 0.17 |  | 0.20 |  | 0.23 |  |
| *ANOVA F* | 12.95 |  | 12.44 |  | 11.31 |  | 9.72 |  | 7.64 |  |
| *p-value* | <1E-9^***^ |  | <1E-9^***^ |  | <1E-9^***^ |  | <1E-9^***^ |  | <1E-9^***^ |  |
| *df Regression* | 7 |  | 11 |  | 9 |  | 12 |  | 18 |  |
| *df Residual* | 495 |  | 491 |  | 493 |  | 473 |  | 467 |  |
| *df Total* | 502 |  | 502 |  | 502 |  | 485 |  | 485 |  |
| *N* | 503 |  | 503 |  | 503 |  | 486 |  | 486 |  |

1. Regression results for gains in knowledge about impacts:

|  | **Model 1:**  **Base model** | | **Model 2:**  **Session-level**  **fixed effects** | | **Model 3:**  **Gender/**  **Age** | | **Model 4:**  **Other**  **demographic**  **info** | | **Model 5:**  **All fixed**  **effects** | |
| --- | --- | --- | --- | --- | --- | --- | --- | --- | --- | --- |
| **Parameter:** | Beta | *p* | Beta | *p* | Beta | *p* | Beta | *p* | Beta | *p* |
| Gain in Affect: Urgency | 0.226 | 1E-08^***^ | 0.214 | 4E-08^***^ | 0.216 | 7E-08^***^ | 0.201 | 6E-07^***^ | 0.213 | 2E-07^***^ |
| Gain in Affect: Hope | 0.050 | 0.180 | 0.004 | 0.922 | 0.038 | 0.315 | 0.020 | 0.599 | 0.006 | 0.876 |
| Pre-Knowledge: Impacts | -0.611 | <1E-9^***^ | -0.637 | <1E-9^***^ | -0.619 | <1E-9^***^ | -0.627 | <1E-9^***^ | -0.637 | <1E-9^***^ |
| Pre-Urgency | 0.263 | 2E-09^***^ | 0.262 | 2E-09^***^ | 0.256 | 6E-09^***^ | 0.245 | 4E-08^***^ | 0.256 | 2E-08^***^ |
| Pre-Hope | 0.038 | 0.306 | 0.006 | 0.865 | 0.030 | 0.421 | 0.016 | 0.683 | 0.007 | 0.853 |
| Percent usable cases |  |  | -0.003 | 0.930 |  |  |  |  | 0.017 | 0.685 |
| Facilitated by core team |  |  | 0.082 | 0.019 |  |  |  |  | 0.083 | 0.026 |
| Setting (Higher Ed or Secondary) |  |  | 0.155 | 3E-05^**^ |  |  |  |  | 0.188 | 0.018 |
| Country type (Developed or Developing) |  |  | -0.036 | 0.320 |  |  |  |  | -0.039 | 0.326 |
| Gender |  |  |  |  | 0.034 | 0.334 |  |  | 0.018 | 0.623 |
| Age |  |  |  |  | 0.092 | 0.010 |  |  | 0.026 | 0.694 |
| Education of Parents |  |  |  |  |  |  | 0.033 | 0.383 | 0.027 | 0.485 |
| Education of Self |  |  |  |  |  |  | 0.091 | 0.027 | -0.095 | 0.288 |
| Science Major |  |  |  |  |  |  | 0.071 | 0.061 | 0.056 | 0.153 |
| Perceived socioeconomic status |  |  |  |  |  |  | -0.028 | 0.468 | -0.034 | 0.396 |
| Favor regulation of free market |  |  |  |  |  |  | 0.014 | 0.697 | 0.023 | 0.538 |
|  |  |  |  |  |  |  |  |  |  |  |
| R^2^ | 0.34 |  | 0.37 |  | 0.35 |  | 0.37 |  | 0.38 |  |
| ANOVA F | 57.09 |  | 35.71 |  | 42.21 |  | 29.90 |  | 19.52 |  |
| p-value | <1E-9^***^ |  | <1E-9^***^ |  | <1E-9^***^ |  | <1E-9^***^ |  | <1E-9^***^ |  |
| df regression | 5 |  | 9 |  | 7 |  | 10 |  | 16 |  |
| df residual | 548 |  | 544 |  | 544 |  | 521 |  | 515 |  |
| df Total | 553 |  | 553 |  | 551 |  | 531 |  | 531 |  |
| N | 554 |  | 554 |  | 552 |  | 532 |  | 532 |  |

1. Regression results for gains in intent to take action.

|  | ***Model 1:***  ***Base model*** | | ***Model 2:***  ***Session-level***  ***fixed effects*** | | ***Model 3:***  ***Gender***  ***Age*** | | ***Model 4:***  ***Other***  ***demographic***  ***info*** | | ***Model 5:***  ***All fixed***  ***effects*** | |
| --- | --- | --- | --- | --- | --- | --- | --- | --- | --- | --- |
| ***Parameter:*** | Beta | *p* | Beta | *p* | Beta | *p* | Beta | *p* | Beta | *p* |
| *Gain in Knowledge: Causes* | 0.062 | 0.189 | 0.055 | 0.243 | 0.057 | 0.218 | 0.088 | 0.065 | 0.083 | 0.078 |
| *Gain in Knowledge: Impacts* | 0.107 | 0.020 | 0.102 | 0.027 | 0.094 | 0.038 | 0.142 | 0.003 | 0.145 | 0.002 |
| *Gain in Knowledge: Stock-Flow* | 0.051 | 0.214 | 0.029 | 0.482 | 0.010 | 0.807 | 0.058 | 0.174 | 0.037 | 0.381 |
| *Gain in Urgency* | 0.313 | <1E-9^***^ | 0.314 | <1E-9^***^ | 0.304 | <1E-9^***^ | 0.327 | <1E-9^***^ | 0.321 | <1E-9^***^ |
| *Gain in Hope* | 0.155 | 6E-05^**^ | 0.149 | 2E-04^*^ | 0.143 | 2E-04^*^ | 0.161 | 5E-05^**^ | 0.177 | 1E-05^**^ |
| *Pre-Knowledge: Causes* | -0.021 | 0.683 | -0.021 | 0.672 | -0.029 | 0.552 | 0.016 | 0.758 | 0.019 | 0.706 |
| *Pre-Knowledge: Impacts* | 0.065 | 0.203 | 0.068 | 0.186 | 0.066 | 0.189 | 0.118 | 0.026 | 0.126 | 0.018 |
| *Pre-Knowledge: Stock-Flow* | 0.073 | 0.081 | 0.030 | 0.492 | 0.019 | 0.648 | 0.074 | 0.103 | 0.061 | 0.179 |
| *Pre-Urgency* | 0.276 | 1E-07 | 0.297 | 2E-08 | 0.303 | 4E-09 | 0.270 | 3E-07 | 0.282 | 1E-07 |
| *Pre-Hope* | 0.139 | 4E-04 | 0.137 | 0.001 | 0.147 | 1E-04 | 0.154 | 2E-04 | 0.176 | 2E-05 |
| *Pre-Intent* | -0.646 | <1E-9^***^ | -0.669 | <1E-9^***^ | -0.704 | <1E-9^***^ | -0.705 | <1E-9^***^ | -0.734 | <1E-9^***^ |
| *Percent usable cases* |  |  | -0.002 | 0.949 |  |  |  |  | -0.023 | 0.590 |
| *Facilitated by core team* |  |  | -0.029 | 0.439 |  |  |  |  | -0.023 | 0.549 |
| *Setting (Higher Ed or Secondary)* |  |  | 0.120 | 0.003 |  |  |  |  | 0.074 | 0.325 |
| *Country type (Developed or Developing)* |  |  | 0.082 | 0.030 |  |  |  |  | -0.016 | 0.693 |
| *Gender* |  |  |  |  | 0.022 | 0.539 |  |  | 0.020 | 0.584 |
| *Age* |  |  |  |  | 0.192 | 6E-07^***^ |  |  | 0.240 | 1E-04^*^ |
| *Education of Parents* |  |  |  |  |  |  | -0.015 | 0.701 | 0.005 | 0.894 |
| *Education of Self* |  |  |  |  |  |  | 0.067 | 0.123 | -0.181 | 0.036 |
| *Science Major* |  |  |  |  |  |  | 0.019 | 0.624 | 0.005 | 0.910 |
| *Perceived socioeconomic status* |  |  |  |  |  |  | 0.002 | 0.969 | 0.010 | 0.804 |
| *Favor regulation of free market* |  |  |  |  |  |  | -0.040 | 0.280 | -0.024 | 0.533 |
|  |  |  |  |  |  |  |  |  |  |  |
| *R^2^* | 0.40 |  | 0.41 |  | 0.44 |  | 0.46 |  | 0.49 |  |
| *ANOVA F* | 28.86 |  | 22.43 |  | 28.05 |  | 22.28 |  | 17.91 |  |
| *p-value* | <1E-9^***^ |  | <1E-9^***^ |  | <1E-9^***^ |  | <1E-9^***^ |  | <1E-9^***^ |  |
| *df Regression* | 11 |  | 15 |  | 13 |  | 16 |  | 22 |  |
| *df Residual* | 480 |  | 476 |  | 472 |  | 425 |  | 418 |  |
| *df Total* | 491 |  | 491 |  | 485 |  | 441 |  | 440 |  |
| *N* | 492 |  | 492 |  | 486 |  | 442 |  | 441 |  |

E. Regression results for desire to learn more.

|  | ***Model 1:***  ***Base model*** | | ***Model 2:***  ***Session-level***  ***fixed effects*** | | ***Model 3:***  ***Gender***  ***Age*** | | ***Model 4:***  ***Other***  ***demographic***  ***info*** | | ***Model 5:***  ***All fixed***  ***effects*** | |
| --- | --- | --- | --- | --- | --- | --- | --- | --- | --- | --- |
| ***Parameter:*** | Beta | p | Beta | *p* | Beta | *p* | Beta | *p* | Beta | *p* |
| *Gain in Urgency* | 0.228 | 3E-06^***^ | 0.216 | 5E-06^***^ | 0.210 | 1E-05^**^ | 0.202 | 3E-05^**^ | 0.208 | 2E-05^**^ |
| *Gain in Hope* | 0.168 | 2E-04^*^ | 0.092 | 0.041 | 0.138 | 0.002 | 0.103 | 0.024 | 0.093 | 0.044 |
| *Gain in Knowledge: Impacts* | 0.159 | 0.003 | 0.110 | 0.033 | 0.134 | 0.009 | 0.118 | 0.024 | 0.105 | 0.045 |
| *Gain in Knowledge: Causes* | 0.014 | 0.800 | 0.000 | 0.998 | 0.013 | 0.808 | 0.010 | 0.848 | 0.005 | 0.923 |
| *Gain in Knowledge: Stock-Flow* | 0.064 | 0.188 | 0.018 | 0.697 | 0.024 | 0.620 | 0.009 | 0.860 | 0.010 | 0.838 |
| *Pre-Urgency* | 0.263 | 3E-06^***^ | 0.248 | 5E-06^***^ | 0.245 | 7E-06^***^ | 0.245 | 9E-06^***^ | 0.237 | 3E-05^**^ |
| *Pre-Hope* | 0.152 | 0.001 | 0.085 | 0.059 | 0.128 | 0.004 | 0.090 | 0.053 | 0.081 | 0.085 |
| *Pre-Knowledge: Impacts* | 0.163 | 0.006 | 0.110 | 0.056 | 0.136 | 0.018 | 0.127 | 0.030 | 0.113 | 0.056 |
| *Pre-Knowledge: Causes* | -0.072 | 0.228 | -0.073 | 0.204 | -0.053 | 0.356 | -0.071 | 0.224 | -0.066 | 0.264 |
| *Pre-Knowledge: Stock-Flow* | 0.053 | 0.280 | -0.014 | 0.771 | -0.018 | 0.707 | -0.048 | 0.351 | -0.039 | 0.460 |
| *Percent usable cases* |  |  | -0.093 | 0.027 |  |  |  |  | -0.041 | 0.393 |
| *Facilitated by core team* |  |  | 0.018 | 0.658 |  |  |  |  | 0.006 | 0.892 |
| *Setting (Higher Ed or Secondary)* |  |  | 0.248 | 6E-08^***^ |  |  |  |  | 0.107 | 0.252 |
| *Country type (Developed or Developing)* |  |  | -0.064 | 0.134 |  |  |  |  | -0.070 | 0.138 |
| *Gender* |  |  |  |  | 0.067 | 0.103 |  |  | 0.049 | 0.241 |
| *Age* |  |  |  |  | 0.236 | 7E-08^***^ |  |  | 0.104 | 0.174 |
| *Education of Parents* |  |  |  |  |  |  | -0.022 | 0.618 | -0.008 | 0.860 |
| *Education of Self* |  |  |  |  |  |  | 0.242 | 1E-06^***^ | 0.055 | 0.608 |
| *Science Major* |  |  |  |  |  |  | 0.099 | 0.026 | 0.073 | 0.114 |
| *Perceived socioeconomic status* |  |  |  |  |  |  | -0.032 | 0.476 | -0.026 | 0.580 |
| *Favor regulation of free market* |  |  |  |  |  |  | 0.013 | 0.760 | 0.026 | 0.553 |
|  |  |  |  |  |  |  |  |  |  |  |
| *R^2^* | 0.15 |  | 0.23 |  | 0.21 |  | 0.22 |  | 0.24 |  |
| *ANOVA F* | 8.92 |  | 10.12 |  | 10.73 |  | 9.01 |  | 6.91 |  |
| *p-value* | <1E-9^***^ |  | <1E-9^***^ |  | <1E-9^***^ |  | <1E-9^***^ |  | <1E-9^***^ |  |
| *df Regression* | 10 |  | 14 |  | 12 |  | 15 |  | 21 |  |
| *df Residual* | 492 |  | 488 |  | 490 |  | 470 |  | 464 |  |
| *df Total* | 502 |  | 502 |  | 502 |  | 485 |  | 485 |  |
| *N* | 503 |  | 503 |  | 503 |  | 486 |  | 486 |  |
