# Supplementary material for: Combining role-play with interactive simulation to motivate informed climate action: Evidence from the World Climate simulation
Source: PLoS One. 2018 Aug 30;13(8):e0202877. doi: 10.1371/journal.pone.0202877 (PMC6117006; doi:10.1371/journal.pone.0202877)
Supplement: S3 Table — Correlation matrices for session-level (A) and participant-level control variables (B). Pearson correlation coefficients are provided, with bold text reflecting correlations that are statistically significant at p < 0.05. (DOCX) [file pone.0202877.s003.docx]

A.

|  |  | 1 | 2 | 3 | 4 | 5 | 6 | 7 | 8 | 9 | 10 | 11 | 12 | 13 |
| --- | --- | --- | --- | --- | --- | --- | --- | --- | --- | --- | --- | --- | --- | --- |
| 1 | *Gain in Affect: Urgency* |  |  |  |  |  |  |  |  |  |  |  |  |  |
| 2 | *Gain in Affect: Hope* | -.007 |  |  |  |  |  |  |  |  |  |  |  |  |
| 3 | *Gain in Knowledge: Impacts* | **.231** | .033 |  |  |  |  |  |  |  |  |  |  |  |
| 4 | *Gain in Knowledge: Stock-Flow* | .054 | -.031 | **.089** |  |  |  |  |  |  |  |  |  |  |
| 5 | *Gain in Intent to Act* | **.315** | **.082** | **.162** | **.105** |  |  |  |  |  |  |  |  |  |
| 6 | *Pre-Urgency* | **-.440** | .039 | **-.091** | -.018 | **-.120** |  |  |  |  |  |  |  |  |
| 7 | *Pre-Hope* | .010 | **-.318** | .037 | -.010 | .003 | **-.135** |  |  |  |  |  |  |  |
| 8 | *Pre-Knowledge: Impacts* | **-.141** | .017 | **-.524** | .017 | **-.093** | **.390** | **-.140** |  |  |  |  |  |  |
| 9 | *Pre-Knowledge: Stock-Flow* | .012 | -.004 | -.020 | **-.453** | -.056 | .062 | -.059 | **.151** |  |  |  |  |  |
| 10 | *Pre-Intent to Act* | **-.156** | .049 | -.058 | -..027 | **-.451** | **.512** | **.083** | **.332** | **.113** |  |  |  |  |
| 11 | *Educational setting* | .004 | **.102** | .060 | .060 | **.094** | **.082** | .038 | **.101** | **.202** | **.194** |  |  |  |
| 12 | *Facilitator training* | .008 | **.169** | .056 | .056 | .064 | -.008 | **.084** | .012 | .008 | .010 | **.083** |  |  |
| 13 | *Country type* | **.082** | **-.124** | .011 | .011 | .052 | **-.113** | **-.239** | .000 | **.101** | **-.178** | **-.266** | **-.294** |  |
| 14 | *% usable cases* | .054 | .036 | .044 | .044 | **.121** | **-.090** | -.047 | **-.078** | -.049 | **-.147** | .025 | **.448** | **.105** |

Note: correlations > + 0.07 are significant at *p* < 0.05 (two-tailed), correlations > + 0.09 are significant at *p* < 0.01 (two-tailed), and correlations > + 0.12 are significant at *p* < 0.001 (two-tailed). N = 858.

Educational setting was coded with secondary school settings = 0 and post-secondary settings = 1; facilitator training was coded as 0 = facilitator was not a member of the project team and used online training tools, and 1 = facilitator was a member of the project team; country type was coded with developing countries = 0 and developed countries = 1.

B.

|  |  | 1 | 2 | 3 | 4 | 5 | 6 | 7 | 8 | 9 | 10 | 11 | 12 | 13 | 14 | 15 | 16 |
| --- | --- | --- | --- | --- | --- | --- | --- | --- | --- | --- | --- | --- | --- | --- | --- | --- | --- |
| 1 | *Gain in Affect: Urgency* |  |  |  |  |  |  |  |  |  |  |  |  |  |  |  |  |
| 2 | *Gain in Affect: Hope* | -.007 |  |  |  |  |  |  |  |  |  |  |  |  |  |  |  |
| 3 | *Gain in Knowledge: Impacts* | **.231** | .033 |  |  |  |  |  |  |  |  |  |  |  |  |  |  |
| 4 | *Gain in Knowledge: Stock-Flow* | .054 | -.031 | **.089** |  |  |  |  |  |  |  |  |  |  |  |  |  |
| 5 | *Gain in Intent to Act* | **.315** | **.082** | **.162** | **.105** |  |  |  |  |  |  |  |  |  |  |  |  |
| 6 | *Pre-Urgency* | **-.440** | .039 | **-.091** | -.018 | **-.120** |  |  |  |  |  |  |  |  |  |  |  |
| 7 | *Pre-Hope* | .010 | **-.318** | .037 | -.010 | .003 | **-.135** |  |  |  |  |  |  |  |  |  |  |
| 8 | *Pre-Knowledge: Impacts* | **-.141** | .017 | **-.524** | .017 | **-.093** | **.390** | **-.140** |  |  |  |  |  |  |  |  |  |
| 9 | *Pre-Knowledge: Stock-Flow* | .012 | -.004 | -.020 | **-.453** | -.056 | .062 | -.059 | **.151** |  |  |  |  |  |  |  |  |
| 10 | *Pre-Intent to Act* | **-.156** | .049 | -.058 | -..027 | **-.451** | **.512** | **.083** | **.332** | **.113** |  |  |  |  |  |  |  |
| 11 | *Gender* | **-.073** | -.015 | -.027 | .025 | -.048 | -.021 | **.074** | .058 | -.005 | .029 |  |  |  |  |  |  |
| 12 | *Age* | **.075** | **.106** | **.078** | .055 | **.111** | **.081** | .021 | **.079** | **.202** | **.238** | .053 |  |  |  |  |  |
| 13 | *Education of Parents* | .061 | -.023 | .010 | .023 | .022 | -.035 | **-.138** | **.103** | **.152** | .035 | -.007 | .004 |  |  |  |  |
| 14 | *Education of Self* | .054 | **.084** | **.083** | .058 | **.076** | **.107** | .000 | **.135** | **.254** | **.242** | .022 | **.794** | **.147** |  |  |  |
| 15 | *Science Major* | -.016 | .041 | .068 | .024 | -.011 | **.101** | **.153** | .044 | **.111** | **.222** | **.072** | **.295** | .004 | **.357** |  |  |
| 16 | *Perceived socioeconomic status* | **-.073** | .018 | -.065 | .036 | -.028 | -.041 | **.157** | -.062 | **-.212** | **-.104** | -.019 | **-.264** | **-.290** | **-.317** | -.043 |  |
| 17 | *Favor regulation of free market* | -.038 | -.029 | -.032 | -.051 | -.051 | **.111** | **-.189** | **.088** | **.170** | .032 | .019 | -.040 | **.118** | .007 | **-.095** | **-.091** |

Note: correlations > + 0.07 are significant at *p* < 0.05 (two-tailed), correlations > + 0.09 are significant at *p* < 0.01 (two-tailed), and correlations > + 0.12 are significant at *p* < 0.001 (two-tailed). N = 858.

Gender is coded as 1 = female, 2 = male, and 3 = other or don’t want to specify; age is coded in categories, such that 1 = 11-13 years, 2 = 14-17 years, 3 = 18-24 years, 4 = 25-35 years, 5 = 36-50 years, 6 = 51-75 years, and 7 = 76+ years; education of parents is coded as 1 = no school, 2 = elementary school only, 3 = secondary school, 4 = some post-secondary education, 5 = Bachelor’s degree or beyond; education of self is coded as 1 = no high school degree, 2 = secondary degree, 3 = some post-secondary education, 4 = Bachelor’s degree or beyond; science major is coded as 0 = not a STEM major in college, 1 = STEM major in college; see survey for perceived socioeconomic status and favor regulation of free market values.
