# Supplementary material for: Combining role-play with interactive simulation to motivate informed climate action: Evidence from the World Climate simulation
Source: PLoS One. 2018 Aug 30;13(8):e0202877. doi: 10.1371/journal.pone.0202877 (PMC6117006; doi:10.1371/journal.pone.0202877)
Supplement: S4 Table — (DOCX) [file pone.0202877.s004.docx]

|  | **Hi-part^1^ mean** | **Low-part^2^ mean** | **Hi-part SD** | **Low-part SD** | **Hi-part N** | **Low-part N** | **T** | **df** | **p-value^1^** |
| --- | --- | --- | --- | --- | --- | --- | --- | --- | --- |
| Gain in Knowledge: Causes | 0.10 | 0.10 | 0.41 | 0.38 | 423 | 426 | 0.20 | 839 | 0.841 |
| Gain in Knowledge: Impacts | 0.04 | 0.03 | 0.12 | 0.48 | 384 | 410 | 0.80 | 820 | 0.423 |
| Gain in Knowledge: Stock-Flow | 0.18 | 0.16 | 0.49 | 0.10 | 429 | 429 | 0.47 | 786 | 0.637 |
| Gain in Affect: Urgency | 0.05 | 0.04 | 0.10 | 0.09 | 429 | 429 | 2.39 | 851 | 0.017 |
| Gain in Affect: Hope | 0.04 | 0.03 | 0.18 | 0.18 | 429 | 429 | 0.60 | 856 | 0.552 |
| Gain in Intent to Act | 0.05 | 0.03 | 0.13 | 0.11 | 429 | 429 | 2.51 | 842 | 0.012 |
| Desire to Learn More | 0.92 | 0.93 | 0.10 | 0.10 | 429 | 429 | -1.18 | 855 | 0.238 |
| Pre-Urgency | 0.73 | 0.76 | 0.45 | 0.43 | 424 | 427 | -0.92 | 847 | 0.357 |
| Pre-Hope | 0.33 | 0.33 | 0.47 | 0.47 | 395 | 413 | -1.98 | 815 | 0.048 |
| Pre-Knowledge: Impacts | 0.88 | 0.90 | 0.13 | 0.10 | 429 | 429 | -0.92 | 847 | 0.357 |
| Pre-Knowledge: Causes | 0.73 | 0.75 | 0.14 | 0.13 | 429 | 429 | -0.01 | 804 | 0.996 |
| Pre-Knowledge: Stock-flow | 0.60 | 0.62 | 0.16 | 0.19 | 429 | 429 | -2.52 | 845 | 0.012 |
| Pre-Intent to Act | 0.79 | 0.83 | 0.16 | 0.14 | 429 | 429 | -1.92 | 832 | 0.055 |

^1^After Bonferroni correction, p-values < 9.6 x 10^-6^, <9.6 x 10^-5^, and 4.8 x 10^-4^ are considered significant at α levels of 0.001 (**^***)^**), 0.01 (**^**^**), and 0.05 (**^*^**) respectively.
