# Supplementary material for: Combining role-play with interactive simulation to motivate informed climate action: Evidence from the World Climate simulation
Source: PLoS One. 2018 Aug 30;13(8):e0202877. doi: 10.1371/journal.pone.0202877 (PMC6117006; doi:10.1371/journal.pone.0202877)
Supplement: S5 Table — (DOCX) [file pone.0202877.s005.docx]

A. Analysis limited to sessions with >50% usable cases out of total participants.

|  | *Pre-only* | *Usable cases* | *Pre-only SD* | *Usable cases SD* | *Pre-only N* | *Usable cases N* | *T* | *df* | *p-value^1^* | *ES^2^* |
| --- | --- | --- | --- | --- | --- | --- | --- | --- | --- | --- |
| *Pre-Knowledge: Causes* | 0.70 | 0.73 | 0.46 | 0.44 | 217 | 528 | -1.01 | 388 | 0.313 | 0.07 |
| *Pre-Knowledge: Impacts* | 0.87 | 0.88 | 0.14 | 0.12 | 216 | 535 | -1.61 | 352 | 0.108 | 0.13 |
| *Pre-Knowledge: Stock-flow* | 0.40 | 0.34 | 0.49 | 0.47 | 199 | 498 | 1.51 | 353 | 0.131 | -0.12 |
| *Pre-Urgency* | 0.72 | 0.73 | 0.15 | 0.14 | 216 | 535 | -1.10 | 361 | 0.271 | 0.09 |
| *Pre-Hope* | 0.62 | 0.60 | 0.18 | 0.16 | 209 | 535 | 1.46 | 355 | 0.146 | -0.12 |
| *Pre-Intent to Act* | 0.80 | 0.80 | 0.17 | 0.16 | 213 | 535 | -0.11 | 361 | 0.916 | 0.01 |
| *Facilitated by core team* | 0.93 | 0.90 | 0.25 | 0.30 | 304 | 535 | 1.63 | 720 | 0.104 | -0.11 |
| *Setting (Higher Ed or Secondary)* | 0.87 | 0.82 | 0.34 | 0.39 | 304 | 535 | 1.87 | 695 | 0.061 | -0.14 |
| *Country type (Developed or Developing)* | 0.82 | 0.81 | 0.38 | 0.39 | 304 | 535 | 0.40 | 642 | 0.688 | -0.03 |
| *Gender* | 1.56 | 1.48 | 0.50 | 0.51 | 268 | 535 | 2.20 | 537 | 0.029 | -0.16 |
| *Age* | 4.24 | 3.85 | 1.22 | 1.22 | 268 | 535 | 4.29 | 533 | 2E-05****** | -0.32 |
| *Education of Parents* | 4.23 | 4.38 | 1.03 | 0.88 | 253 | 512 | -1.98 | 436 | 0.048 | 0.16 |
| *Education of Self* | 3.45 | 3.30 | 1.03 | 1.09 | 253 | 512 | 1.81 | 533 | 0.071 | -0.14 |
| *Science Major* | 0.51 | 0.48 | 0.50 | 0.50 | 260 | 524 | 0.86 | 516 | 0.392 | -0.06 |
| *Perceived socioeconomic status* | 4.28 | 4.35 | 2.17 | 2.08 | 261 | 524 | -0.45 | 501 | 0.651 | 0.03 |
| *Favor regulation of free market* | 3.19 | 3.14 | 1.28 | 1.21 | 212 | 525 | 0.43 | 371 | 0.668 | -0.04 |

B. Analysis including all sessions, including those with low rates of data collection.

|  | **Pre-only** | **Usable cases** | **Pre-only SD** | **Usable cases SD** | **Pre-only N** | **Usable cases N** | **T** | **df** | **p-value^1^** | **ES**^2^ |
| --- | --- | --- | --- | --- | --- | --- | --- | --- | --- | --- |
| Pre-Knowledge: Causes | 0.69 | 0.74 | 0.46 | 0.44 | 568 | 851 | -2.21 | 1166 | 0.027 | 0.11 |
| Pre-Knowledge: Impacts | 0.85 | 0.89 | 0.14 | 0.12 | 560 | 858 | -5.60 | 1054 | 3E-08*** | 0.31 |
| Pre-Knowledge: Stock-flow | 0.29 | 0.33 | 0.45 | 0.47 | 507 | 808 | -1.66 | 1106 | 0.097 | 0.09 |
| Pre-Urgency | 0.72 | 0.74 | 0.15 | 0.13 | 561 | 858 | -2.36 | 1074 | 0.019 | 0.13 |
| Pre-Hope | 0.63 | 0.61 | 0.19 | 0.18 | 526 | 858 | 2.42 | 1067 | 0.016 | -0.13 |
| Pre-Intent to Act | 0.78 | 0.81 | 0.17 | 0.15 | 546 | 858 | -3.58 | 1076 | 4E-04* | 0.20 |
| Facilitated by core team | 0.51 | 0.71 | 0.50 | 0.45 | 781 | 858 | -8.51 | 1579 | <1E-9*** | 0.42 |
| Setting (Higher Ed or Secondary) | 0.62 | 0.82 | 0.49 | 0.39 | 781 | 858 | -9.00 | 1487 | <1E-9*** | 0.46 |
| Country type (Developed or Developing) | 0.75 | 0.76 | 0.43 | 0.43 | 781 | 858 | -0.22 | 1621 | 0.825 | 0.02 |
| Gender | 1.53 | 1.46 | 0.54 | 0.51 | 634 | 855 | 2.41 | 1321 | 0.016 | -0.13 |
| Age | 3.82 | 3.71 | 1.34 | 1.24 | 543 | 837 | 1.44 | 1094 | 0.151 | -0.08 |
| Education of Parents | 4.26 | 4.40 | 1.08 | 0.90 | 610 | 830 | -2.68 | 1172 | 0.007 | 0.14 |
| Education of Self | 2.92 | 3.21 | 1.30 | 1.18 | 607 | 830 | -4.28 | 1227 | 2E-05** | 0.23 |
| Science Major | 0.43 | 0.48 | 0.50 | 0.50 | 598 | 833 | -1.97 | 1294 | 0.049 | 0.10 |
| Perceived socioeconomic status | 4.31 | 4.30 | 2.15 | 2.02 | 573 | 831 | 0.12 | 1180 | 0.907 | 0.00 |
| Favor regulation of free market | 3.23 | 3.17 | 1.15 | 1.19 | 540 | 838 | 0.94 | 1174 | 0.349 | -0.05 |

^1^ After Bonferroni correction, p-values < 9.6 x 10^-6^, <9.6 x 10^-5^, and 4.8 x 10^-4^ are considered significant at α levels of 0.001 (**^***)^**), 0.01 (**^**^**), and 0.05 (**^*^**) respectively.

^2^ES denotes Cohen’s *d* effect size.
