# Supplementary material for: Combining role-play with interactive simulation to motivate informed climate action: Evidence from the World Climate simulation
Source: PLoS One. 2018 Aug 30;13(8):e0202877. doi: 10.1371/journal.pone.0202877 (PMC6117006; doi:10.1371/journal.pone.0202877)
Supplement: S6 Table — (DOCX) [file pone.0202877.s006.docx]

|  | Pre-mean | Post-mean | Post-Pre | SD (Pre) | SD (Post) | N | T | df | p-value^1^ | ES^2^ |
| --- | --- | --- | --- | --- | --- | --- | --- | --- | --- | --- |
| Causes | 0.76 | 0.84 | 0.08 | 0.43 | 0.37 | 613 | -4.97 | 612 | 9E-07*** | 0.20 |
| Impacts | 0.88 | 0.91 | 0.03 | 0.12 | 0.12 | 613 | -6.09 | 612 | 2E-09*** | 0.25 |
| Stock-flow understanding | 0.37 | 0.51 | 0.14 | 0.48 | 0.50 | 568 | -6.98 | 567 | <1E-9*** | 0.29 |
| Urgency | 0.73 | 0.78 | 0.05 | 0.14 | 0.13 | 619 | -12.22 | 618 | <1E-9*** | 0.37 |
| Hope | 0.58 | 0.62 | 0.03 | 0.17 | 0.20 | 609 | -4.65 | 608 | 4E-06*** | 0.16 |
| Intent | 0.79 | 0.83 | 0.04 | 0.15 | 0.15 | 611 | -8.23 | 610 | <1E-9*** | 0.27 |

^1^ After Bonferroni correction, p-values < 9.6 x 10^-6^, <9.6 x 10^-5^, and 4.8 x 10^-4^ are considered significant at α levels of 0.001 (**^***)^**), 0.01 (**^**^**), and 0.05 (**^*^**) respectively.

^2^ES denotes Cohen’s *d* effect size.
