# Supplementary material for: Combining role-play with interactive simulation to motivate informed climate action: Evidence from the World Climate simulation
Source: PLoS One. 2018 Aug 30;13(8):e0202877. doi: 10.1371/journal.pone.0202877 (PMC6117006; doi:10.1371/journal.pone.0202877)
Supplement: S1 Appendix — (PDF) [file pone.0202877.s007.pdf]

China

To: Negotiators for China at United Nations World Climate Summit
Subject: Briefing on Negotiating Goals

Goals

China seeks to negotiate a global agreement to reduce greenhouse gas emissions that can limit climate risks but also seeks the best outcome for our economy and vital national interests. At the 2015 UN climate negotiations in Paris, nations agreed to a goal of limiting global warming to "well below 2˚C" compared to preindustrial levels. You must now decide on the following:

1. Actions to reduce China's emissions, if any. Without action, China's emissions are expected to grow significantly by 2100. You can decide when your emissions will stop growing, when they will begin declining, and at what annual rate emissions decline, if at all.
2. Whether to make a commitment to reduce deforestation or to increase reforestation or afforestation.
3. How much you will contribute to, or request from, the Green Climate Fund, which is intended to provide at least $100 billion/year by 2020 for developing countries to reduce their emissions and adapt to climate change.

Context

The scientific consensus on climate is clear: over 97% of climate scientists agree that climate change is happening, that it is caused primarily by use of fossil fuels, and that the impacts could be devastating. China is vulnerable to climate change, as the country's water, food, and energy systems are already stressed and expected to face increasing pressure due to rising sea levels, droughts, water scarcity, and heat waves.

Public Opinion

Our people, like everywhere else, want to live in a healthy environment, but they also want to increase their standard of living; this includes having decent jobs, housing, food, healthcare, mobility and national security. There is widespread acknowledgement in your country that climate change is real, though few support agreements to address the associated risks.

Opportunities

Besides slowing climate change, reaching an agreement to limit emissions would also reduce local air pollution, which is affecting the health of our people and, in some cases, creating political unrest. In addition, our economy is growing rapidly, and we are emerging as a superpower that can leverage business opportunities associated with an energy transition away from fossil fuels.

National Action

Above all, we must defend our right to develop. Nevertheless, we recognize that our reliance on fossil fuels, particularly coal, is causing negative health impacts. Ahead of the Paris climate talks in 2015, we pledged to peak our carbon dioxide emissions by no later than 2030. Meeting this goal requires increasing renewable and zero-emissions electricity generation capacity to a level equivalent to the entire coal-fired capacity in China today, or roughly all current US electricity generation capacity. The success of current efforts will determine whether we can meet our pledge and even improve on it.

Forests and land use

We have some opportunity to make reductions in emissions from deforestation and land degradation (REDD). Most of the world's remaining tropical forests are in developing countries, and, unfortunately, there is substantial deforestation occurring. Programs to protect forests can reduce emissions.

- The developed nations created the climate crisis and so should take responsibility for addressing it. These nations built their economies on fossil fuels, and now, while we are finally raising our own people out of poverty and manufacturing the bulk of their consumer goods, we cannot shoulder an unfair burden of reductions. Our emissions per person are still only about 40% of those of the US. Our average standard of living, though rising, is still far lower than that in the US, Europe, Japan, and other developed nations.
- China is a developing nation but may still face pressure to contribute to the Green Climate Fund since we are now the world's second largest economy. Any commitments we make should require significant commitments to action by the US, EU, and other developed nations.
- The developed nations fear the rapid economic development we are now, finally, enjoying, and may seek to use a global climate agreement to slow our growth, limit our markets, and constrain our diplomatic and military influence around the world. Meanwhile, the US has pledged to reduce their emissions by only 26- 28% by 2025 from 2005 levels. This pledge faces strong political opposition from members of the US Congress and business interests with a stake in continued fossil fuel consumption.

CO_2 Emissions from Fossil Fuels and Cement

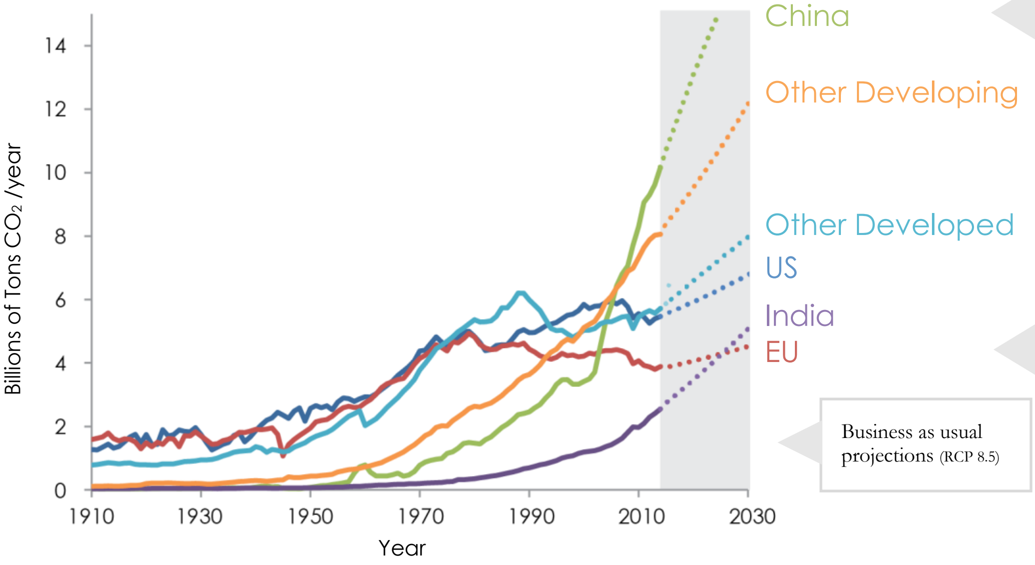

China is the world's largest emitter of CO_2. Without action, developing countries' emissions from fossil fuels are projected to more than triple by 2100.

Sweden sustained annual emissions reductions of 4.5% to reduce their dependence on oil (1976-1986). France and Belgium saw similar reductions around this time. Otherwise, most significant historical emission reductions have come from financial or political crises. According to UNEP, a 3.5% annual reduction rate is extremely ambitious.

GDP per person (2011 PPP US Dollars)

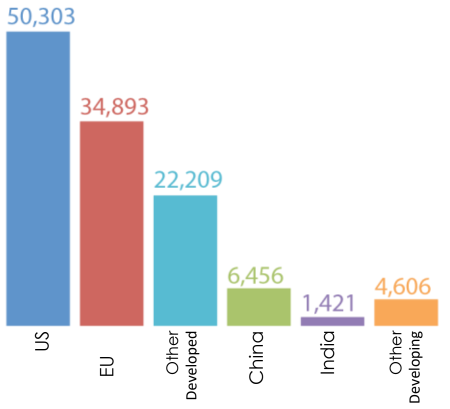

Population Wealth and Cumulative Emissions

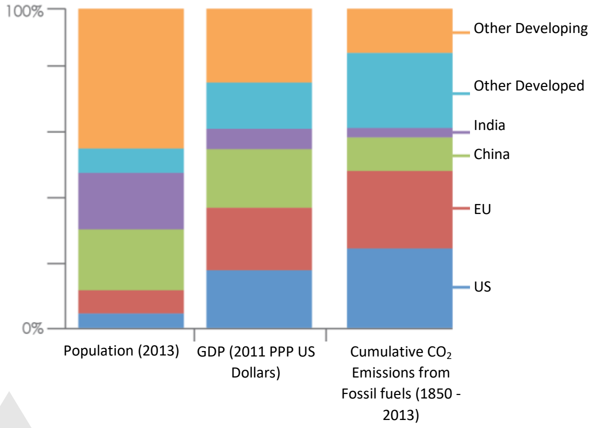

Emissions per person
2013 (tons CO_2 per year)

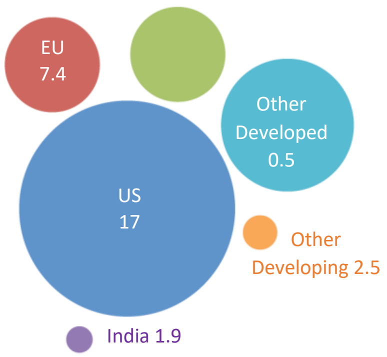

While cumulative emissions so far have been higher in the developed countries (i.e., the **US**, **EU**, and **other developed countries**), the growth of population, GDP per person, and emissions in the developing nations far outpaces growth in the developed countries. Under business-as-usual assumptions, cumulative emissions of all developed countries (**US**, **EU**, and **other developed**) are expected to fall to 37% of total by 2100.

Since 1980, emissions per person have risen dramatically in **China** and **India** (by 391% and 285%, respectively) but have fallen in the **US** and **Europe** (by 20% and 26%, respectively).

European Union

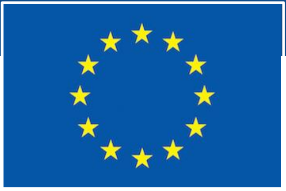

To: European Union negotiators at United Nations World Climate Summit
Subject: Briefing on Negotiating Goals

Goals

The nations of the EU seek to negotiate a global agreement to reduce greenhouse gas emissions that achieves the best outcome for our economies and national interests, as well as for the world. At the 2015 UN climate negotiations in Paris, nations agreed to a goal of limiting global warming to "well below 2°C" compared to preindustrial levels. You must now decide on the following:

1. Actions to reduce carbon emissions, if any. Despite modest declines in emissions since 1990, without continued action, EU emissions are expected to grow. You can decide when EU emissions will stop growing, when they will begin declining, and at what annual rate emissions decline, if at all.
2. Whether to make a commitment to reduce deforestation or to increase reforestation or afforestation.
3. How much the EU will contribute, if at all, to the Green Climate Fund, which is intended to provide at least $100 billion per year by 2020 for developing countries to reduce their emissions and adapt to climate change.

Context

The scientific consensus on climate is clear: over 97% of climate scientists agree that climate change is happening, that it is caused primarily by use of fossil fuels, and that the impacts could be devastating. EU research has shown that climate change is already affecting our nations and that without dramatic reductions in global emissions, the damage will become far more severe.

Public Opinion

The vast majority of the public in our countries believes climate change is real and that human activity contributes significantly to it. Most support international agreements to address climate change. However, most also oppose higher taxes on energy or other actions that will raise the cost of living. For most EU citizens, climate change is not a top priority, ranking below concerns about financial instability, unemployment, migration, and terrorism. Most importantly, the public is strongly opposed to any agreement that does not include meaningful commitments to reduce emissions by the US and rapidly developing economies, particularly China.

Opportunities

Reducing emissions has multiple benefits, beyond climate stability. For example, renewable energy development is creating jobs, reducing dependence on foreign oil and gas, and reducing air pollution.

National Action

The EU has been a leader in the fight against climate change. At the climate negotiations in Paris, the EU pledged to reduce emissions by at least 40% by 2030, compared to 1990 levels. Our pledge is among the most ambitious of the major emitters, but it requires major new regulations that have yet to be implemented and must be realized during a time when we are facing significant economic and security challenges. Despite these potential limitations, the EU has pioneered economic policy that puts a price on greenhouse gas emissions, and we are leaders in deploying renewable energy sources such as wind and solar. We will continue to lead but we cannot and will not move alone.

Forests and land use

Though we can pledge reductions in emissions from deforestation and land degradation (REDD) within our country, doing so would address only a small portion of EU emissions.

Global Landscape

• Emissions in China, India and other developing nations are growing rapidly. China alone is now responsible for 30% of global CO_2 emissions. Total emissions from the developing countries will soon overwhelm emissions from all developed nations.

• The US has more than double the per capita emissions of the EU and has pledged to reduce their emissions by only 26-28% by 2025 from 2005 levels. Additionally, this pledge faces strong political opposition from members of the US Congress and business interests with a stake in continued fossil fuel consumption. In spite of these challenges, US research, including the bipartisan "Risky Business" report (http://riskybusiness.org), endorsed by former US Treasury Secretaries of both parties, shows that the costs of delay are high while most states and regions in the US will benefit from policies that reduce emissions.

• The less developed nations continue to emphasize that reductions in their emissions would require extensive financial assistance from developed countries, but corruption pervades many of these countries and financial assistance often fails to reach its intended use. They may also emphasize forestry policy over cutting fossil fuel emissions, which, while important, is insufficient for meeting the climate challenge.

CO_2 Emissions from Fossil Fuels and Cement

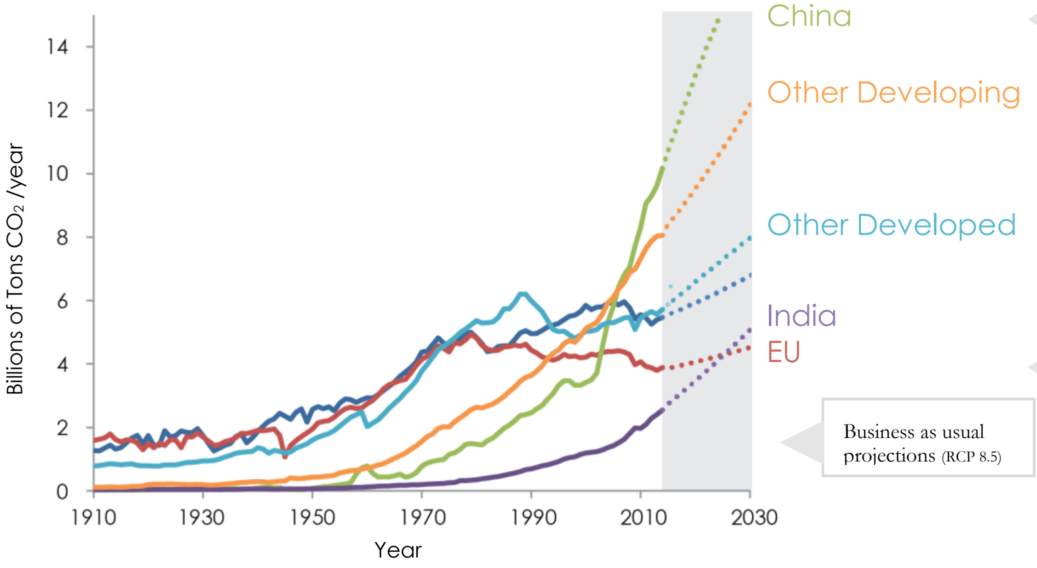

China is the world's largest emitter of CO_2. Without action, developing countries' emissions from fossil fuels are projected to more than triple by 2100.

Sweden sustained annual emissions reductions of 4.5% to reduce their dependence on oil (1976-1986). France and Belgium saw similar reductions around this time. Otherwise, most significant historical emission reductions have come from financial or political crises. According to UNEP, a 3.5% annual reduction rate is extremely ambitious.

GDP per person (2011 PPP US Dollars)

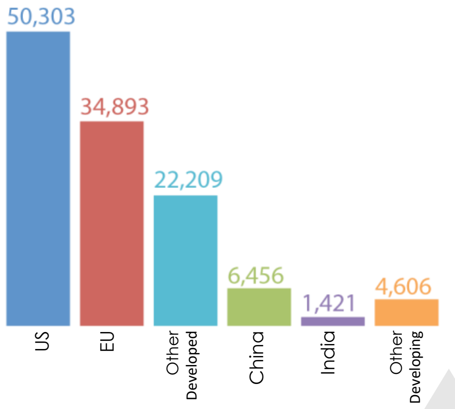

Population Wealth and Cumulative Emissions

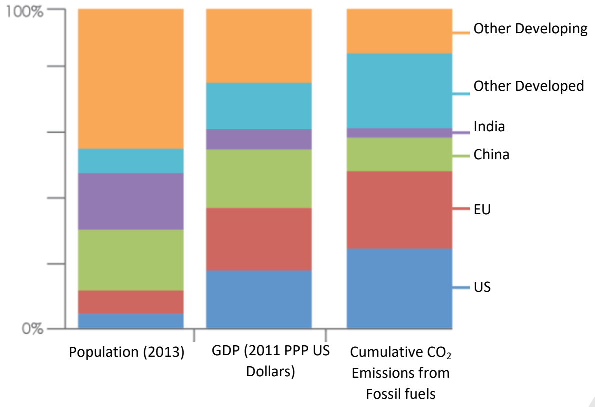

Emissions per person
2013 (tons CO_2 per year)

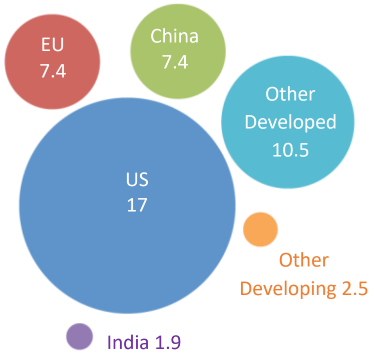

While cumulative emissions so far have been higher in the developed countries (i.e., the **US**, **EU**, and **other developed countries**), the growth of population, GDP per person, and emissions in the developing nations far outpaces growth in the developed countries. Under business-as-usual assumptions, cumulative emissions of all developed countries (**US**, **EU**, and **other developed**) are expected to fall to 37% of total by 2100.

Since 1980, emissions per person have risen dramatically in **China** and **India** (by 391% and 285%, respectively) but have fallen in the **US** and **Europe** (by 20% and 26%, respectively).

India

To: Negotiators for India at United Nations World Climate Summit
Subject: Briefing on Negotiating Goals

Goals

India seeks to negotiate a global agreement to reduce greenhouse gas emissions that can limit climate risks but also seeks the best outcome for our economy and development. At the 2015 UN climate negotiations in Paris, nations agreed to a goal of limiting global warming to "well below 2˚C" compared to preindustrial levels. You must now decide on the following:

1. Actions to reduce carbon emissions, if any. Without action, India's emissions are expected to grow significantly by 2100. You can decide when your emissions will stop growing, when they will begin declining, and at what annual rate emissions decline, if at all.
2. Whether to make a commitment to reduce deforestation or to increase reforestation or afforestation.
3. How much funding to demand from the developed nations, who have pledged to create the Green Climate Fund that will provide $100 billion/year by 2020 for developing countries to cut emissions and adapt to climate change.

Context

The scientific consensus on climate is clear: over 97% of climate scientists agree that climate change is happening, that it is caused primarily by use of fossil fuels, and that the impacts could be devastating. Our top priority is to raise India's standard of living, which is far less than that in the developed nations and even China.

Public Opinion

Our people, like people everywhere, want to live in a healthy environment, but they also want to increase their standard of living; this includes decent jobs, housing, food, healthcare, mobility and national security. Many people in our country believe climate change is real, though fewer support agreements to address the climate change issue. Overcoming poverty is our top priority.

Forests and land use

We have an opportunity to make reductions in emissions from deforestation and land degradation (REDD). Most of the world's remaining tropical forests are in developing countries, where, unfortunately, there is substantial deforestation occurring. Programs to protect forests can reduce global emissions and could be paid for by wealthy nations.

National Action

In 2015 we pledged to the UN to increase our carbon-free power capacity from 30% to 40% by 2030, assuming international help. We are yet to commit to meaningful reductions in emissions and are concerned about pressure to do so given our need to raise a significant portion of our population out of poverty. Clean energy development to reduce emissions would offer co-benefits like improving air quality and providing electricity in areas inaccessible to electric grids. Taking more leadership in addressing climate change could have some political benefits for India. However, we must defend our right to develop and lift our people out of poverty. We are prepared to do what we can, but the rich nations of the world must agree to significant action, commensurate with their past contributions the problem. We will not pay the price for their past emissions.

Global Landscape

• The developed nations will pressure us to reduce emissions because our population exceeds one billion, and because our economy and carbon emissions are growing faster than theirs. However, emissions per capita in developed nations are far higher than ours (US emissions per capita are an astounding 9 times higher than India's). Any agreement that puts an unfair burden of limiting climate change on us is not acceptable.

• The developed nations are threatened by the rapid economic development we are now, finally, beginning to enjoy, and may use a global climate agreement to slow our growth, limit markets for our products, and constrain our growing diplomatic and military influence around the world.

• The developed nations of the world created the climate crisis and must take responsibility for their past actions. These nations used fossil fuels to build their economies and enrich their populations, often by exploiting our natural resources. The developed nations will demand that we cut our emissions before our people have the chance to reach even the level of economic development now enjoyed in the West while many Indians continue to live in poverty.

CO_2 Emissions from Fossil Fuels and Cement

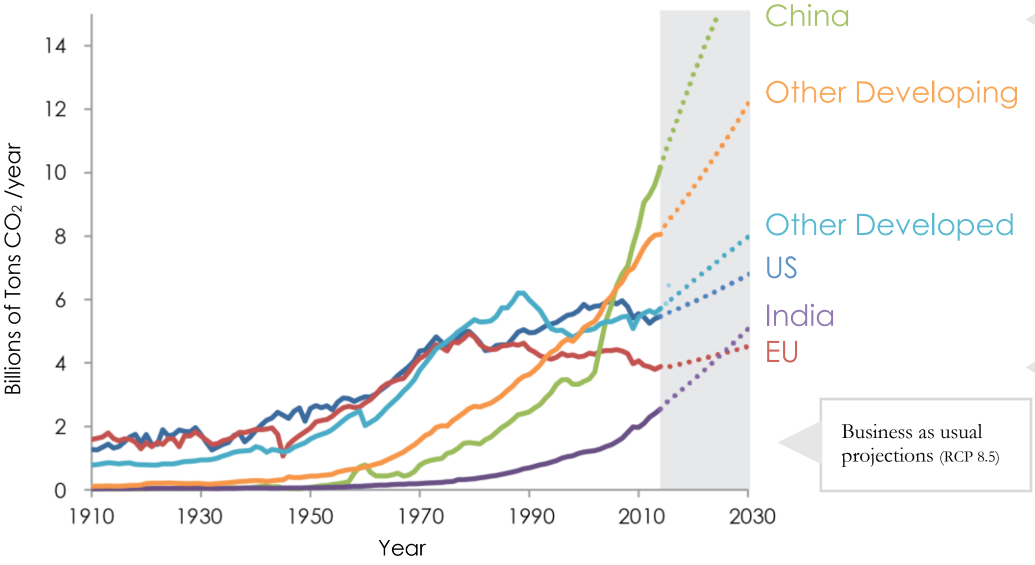

China is the world's largest emitter of CO_2. Without action, developing countries' emissions from fossil fuels are projected to more than triple by 2100.

Sweden sustained annual emissions reductions of 4.5% to reduce their dependence on oil (1976-1986). France and Belgium saw similar reductions around this time. Otherwise, most significant historical emission reductions have come from financial or political crises. According to UNEP, a 3.5% annual reduction rate is extremely ambitious.

GDP per person (2011 PPP US Dollars)

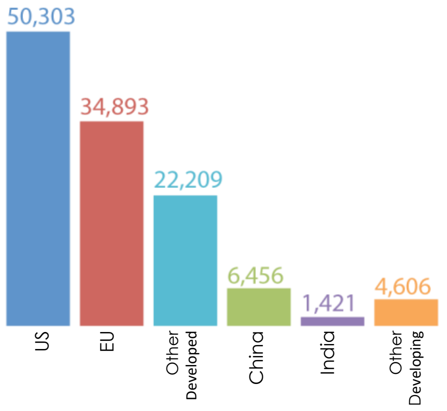

Population Wealth and Cumulative Emissions

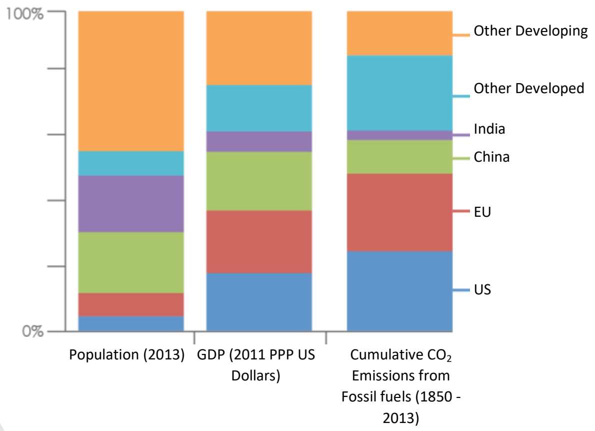

Emissions per person

2013 (tons CO_2 per year)

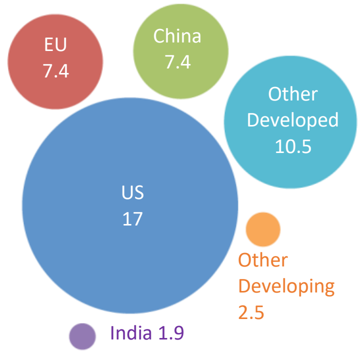

While cumulative emissions so far have been higher in the developed countries (i.e., the **US**, **EU**, and **other developed countries**), the growth of population, GDP per person, and emissions in the developing nations far outpaces growth in the developed countries. Under business-as-usual assumptions, cumulative emissions of all developed countries (**US**, **EU**, and **other developed**) are expected to fall to 37% of total by 2100.

Since 1980, emissions per person have risen dramatically in **China** and **India** (by 391% and 285%, respectively) but have fallen in the **US** and **Europe** (by 20% and 26%, respectively).

Other Developed Nations

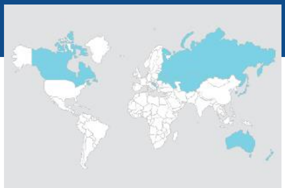

To: Negotiators for Other Developed Nations at UN World Climate Summit
Subject: Briefing on Negotiating Goals
Includes: Australia, Canada, Japan, New Zealand, Russia and other former Soviet Republics, South Korea, etc.

Goals

The Other Developed Nations seek to negotiate a global agreement to reduce greenhouse gas emissions that achieves the best outcome for our economies and national interests, as well as for the world. At the 2015 UN climate negotiations in Paris, nations agreed to a goal of limiting global warming to "well below 2°C" compared to preindustrial levels. You must now decide on the following:

1. Actions to reduce CO_2 emissions, if any. Without action, your emissions are expected to grow over time. You can decide when emissions will stop growing, when they will begin declining, and at what annual rate emissions decline, if at all.
2. Whether to make a commitment to reduce deforestation or to increase reforestation or afforestation.
3. How much you will contribute, if at all, to the Green Climate Fund, which is intended to provide at least $100 billion/year by 2020 for developing countries to reduce their emissions and adapt to climate change.

Context

The scientific consensus on climate is clear: over 97% of climate scientists agree that climate change is happening, that it is caused primarily by use of fossil fuels, and that the impacts could be devastating. Many developed countries are feeling the effects right now, from rising sea levels to prolonged droughts.

Public Opinion

A majority of the public in our countries believe climate change is real and that human activity contributes significantly to it. Most support policies that could be implemented to address climate change but oppose those actions that raise the cost of living. Climate change ranks near the bottom of most people's priorities, far below national security, the economy and jobs. The public is strongly opposed to any agreement that does not require commitments by the US and developing nations.

Opportunities

Fortunately, especially as renewable energy becomes more affordable, reducing emissions could improve public health, create jobs, and improve energy security.

Forests and land use

Though we can pledge reductions in emissions from deforestation and land degradation (REDD) within our bloc, doing so would only address a small portion of our emissions.

National Action

In Paris, our countries made commitments to address climate change that, when combined, would stop the growth in emissions and then reduce emissions slightly. From Canada's tar sands to Australia's coal and Russia's oil and gas, many of our economies depend on fossil fuels for revenue and jobs. Canada withdrew from the last major climate agreement, the Kyoto Protocol. Australia had a carbon tax policy, but repealed it. However, with awareness about climate change spreading and the costs of renewable energy declining, action on climate change is accelerating in many places. Any agreement that puts the greatest economic burden of limiting climate change on us is not politically acceptable. The rest of the world must agree to significant action if there is to be an effective international agreement.

Developed by Climate Interactive, MIT Sloan, and the UML Climate Change Initiative. Updated: Feb 2017

Global Landscape

• Emissions in China, India and other developing nations are growing rapidly. China alone is now responsible for 30% of global CO2 emissions. Total emissions from the developing countries will soon overwhelm emissions from all developed nations.

• The US has more than double the per capita emissions of the EU and has pledged to reduce their emissions by only 26-28% by 2025 from 2005 levels. Additionally, this pledge faces strong political opposition from members of the US Congress and business interests with a stake in continued fossil fuel consumption. In spite of these challenges, US research, including the bipartisan "Risky Business" report (http://riskybusiness.org), endorsed by former US Treasury Secretaries of both parties, shows that the costs of delay are high while most states and regions in the US will benefit from policies that reduce emissions.

• The less developed nations continue to emphasize that reductions in their emissions would require extensive financial assistance from developed countries, but corruption pervades many of these countries and financial assistance often fails to reach its intended use. They may also emphasize forestry policy over cutting fossil fuel emissions, which, while important, is insufficient for meeting the climate challenge.

CO_2 Emissions from Fossil Fuels and Cement

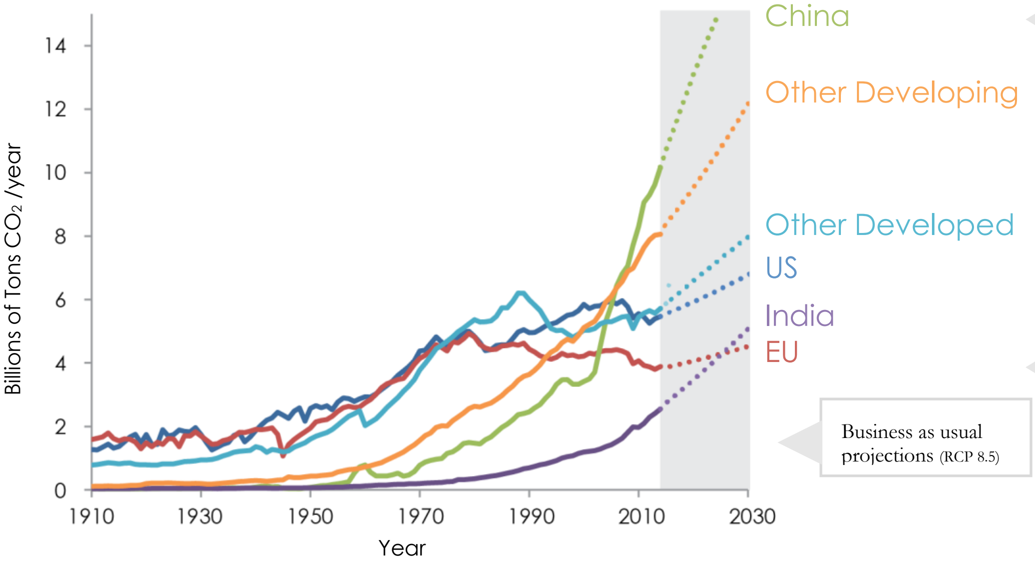

China is the world's largest emitter of CO_2. Without action, developing countries' emissions from fossil fuels are projected to more than triple by 2100.

Sweden sustained annual emissions reductions of 4.5% to reduce their dependence on oil (1976-1986). France and Belgium saw similar reductions around this time. Otherwise, most significant historical emission reductions have come from financial or political crises. According to UNEP, a 3.5% annual reduction rate is extremely ambitious.

GDP per person (2011 PPP US Dollars)

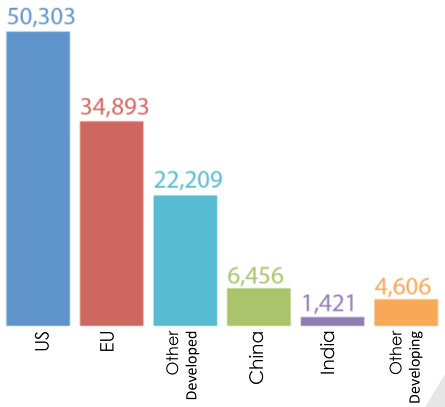

Population Wealth and Cumulative Emissions

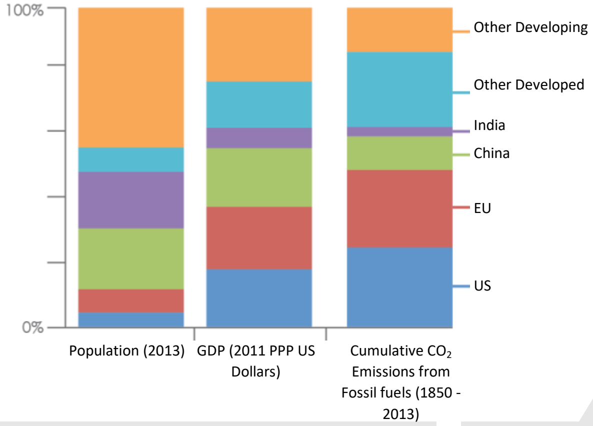

Emissions per person

2013 (tons CO_2 per year)

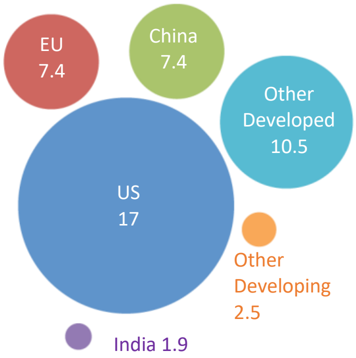

While cumulative emissions so far have been higher in the developed countries (i.e., the **US**, **EU**, and **other developed countries**), the growth of population, GDP per person, and emissions in the developing nations far outpaces growth in the developed countries. Under business-as-usual assumptions, cumulative emissions of all developed countries (**US**, **EU**, and **other developed**) are expected to fall to 37% of total by 2100.

Since 1980, emissions per person have risen dramatically in **China** and **India** (by 391% and 285%, respectively) but have fallen in the **US** and **Europe** (by 20% and 26%, respectively).

Other Developing Nations

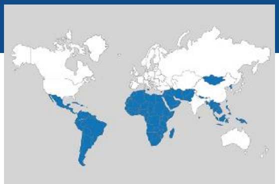

To: Negotiators for Other Developing Nations at UN World Climate Summit
Subject: Briefing on Negotiating Goals
Includes: Africa, Central and South America, South and Southeast Asia, most of the Middle East, and the island nations of the Pacific, Indian Ocean, and the Caribbean

Goals

Our governments seek to negotiate a global agreement to reduce greenhouse gas emissions that achieves the best outcome for our economies and vital national interests, as well as for the world. At the 2015 UN climate negotiations in Paris, nations agreed to a goal of limiting global warming to "well below 2°C above pre-industrial levels and pursuing efforts to limit the temperature increase to 1.5°C above pre- industrial levels." You must now decide on the following:

1. Actions to reduce carbon emissions, if any. Without action, our emissions are expected to continue growing dramatically. You can decide when our emissions will stop growing, when they will begin declining, and at what annual rate emissions decline, if at all.
2. Whether to make a commitment to reduce deforestation or to increase reforestation or afforestation.
3. How much funding to demand from the developed nations, who have pledged to create the Green Climate Fund to provide at least $100 billion/year by 2020 for developing countries to cut emissions and adapt.

Context

The scientific consensus on climate is clear: over 97% of climate scientists agree that climate change is happening, that it is caused primarily by use of fossil fuels, and that the impacts could be devastating. We are highly vulnerable to the drought, disease, flooding, declining agricultural productivity, and ecological disruption caused by climate change. Our survival is at stake and, though other nations may forget, the agreement in Paris sets out a goal of limiting warming to 1.5°C, which we worked hard to ensure was included. As some of the poorest nations of the world, we do not have the resources to fund the needed investments in clean energy technology to reduce our emissions or even to adapt to climate change right now. Therefore, taking bold action is hard to imagine.

Public Opinion

There is rising public concern about climate change as people witness its effects first hand. We know that without action we will be most adversely affected by climate change: sea level rise will displace hundreds of millions of our people, and some nations, such as low-lying islands, will be lost entirely. Our people want to live in a healthy environment where they are able to feed, clothe, and house their families, have a chance for a decent job, and have access to the healthcare the rich nations enjoy.

Forests and land use

We have an opportunity to make reductions in emissions from deforestation and land degradation (REDD). Most of the world's remaining tropical forests are in developing countries, where, unfortunately, there is substantial deforestation occurring. Programs to protect forests can reduce global emissions and could be paid for by wealthy nations. On the reverse of this page you will find some data that may be helpful in your negotiations.

National Action

Above all we must defend our ability to develop and lift our people out of poverty. We are prepared to do what we can to cut our greenhouse gas emissions and all but a few countries have made pledges to the UN to address climate change. Most of these pledges do not include a plan to peak emissions yet, but are offers to grow our greenhouse gas emissions at a slower rate until 2030. The rich nations of the world must agree to more significant action, commensurate with their past contributions to the problem. We will not continue to pay the price for their past emissions.

Global Landscape

• The developed nations will pressure us to reduce our emissions, which are growing faster than theirs. However, their emissions per person are far higher than ours. For example, US emissions per person are an astounding 400 times higher than those in Mali.

• The developed nations of the world created the climate crisis and must take responsibility for their past actions. They used fossil fuels to build their economies and enrich their populations, often by exploiting our people and natural resources. They will demand that we cut our emissions before we have the chance to reach anything close to the level of economic development they now enjoy.

CO_2 Emissions from Fossil Fuels and Cement

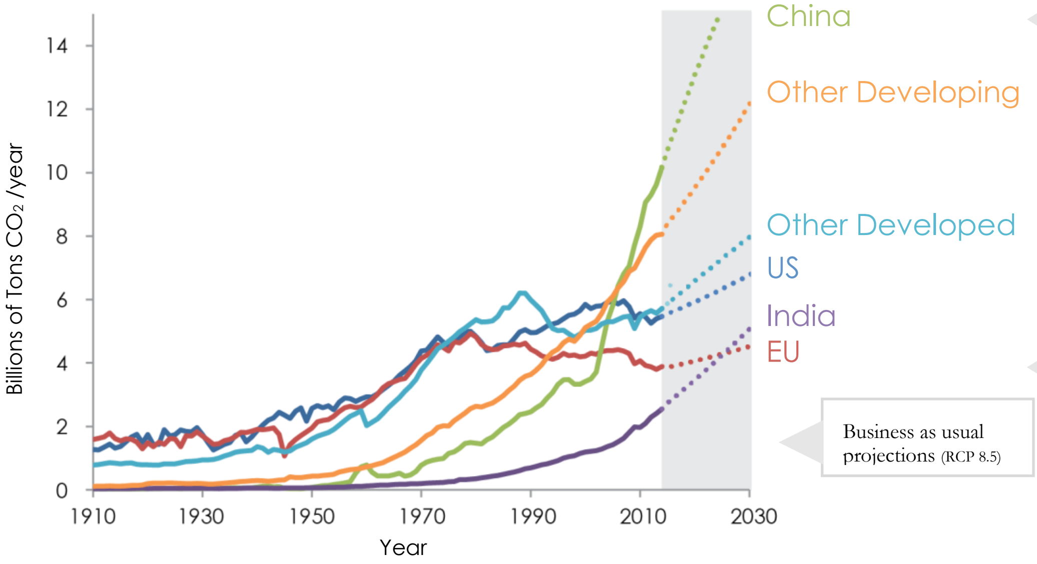

China is the world's largest emitter of CO_2. Without action, developing countries' emissions from fossil fuels are projected to more than triple by 2100.

Sweden sustained annual emissions reductions of 4.5% to reduce their dependence on oil (1976-1986). France and Belgium saw similar reductions around this time. Otherwise, most significant historical emission reductions have come from financial or political crises. According to UNEP, a 3.5% annual reduction rate is extremely ambitious.

GDP per person (2011 PPP US Dollars)

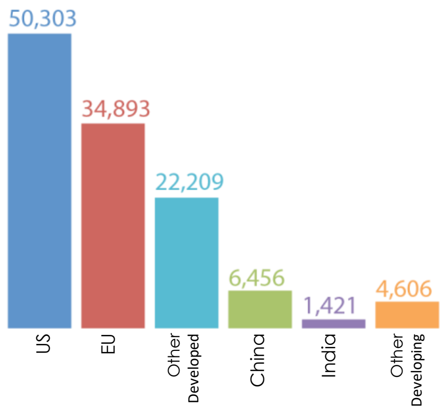

Population Wealth and Cumulative Emissions

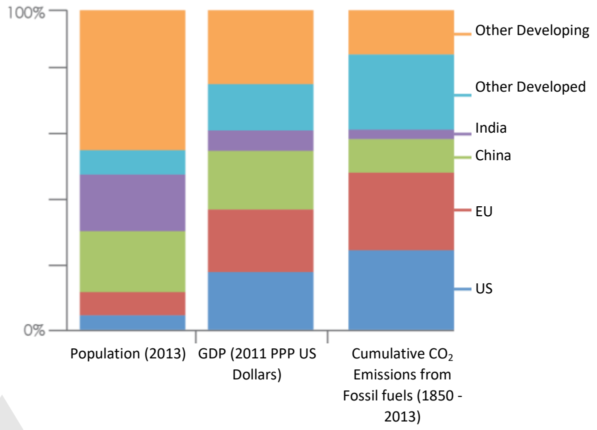

Emissions per person 2013 (tons CO_2 per year)

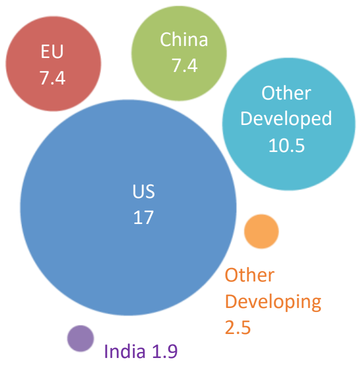

While cumulative emissions so far have been higher in the developed countries (i.e., the **US**, **EU**, and **other developed countries**), the growth of population, GDP per person, and emissions in the developing nations far outpaces growth in the developed countries. Under business-as-usual assumptions, cumulative emissions of all developed countries (**US**, **EU**, and **other developed**) are expected to fall to 37% of total by 2100.

Since 1980, emissions per person have risen dramatically in **China** and **India** (by 391% and 285%, respectively) but have fallen in the **US** and **Europe** (by 20% and 26%, respectively).

United States of America

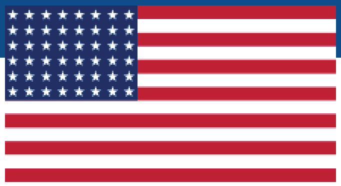

To: United States Negotiators at United Nations World Climate
Subject: Briefing on Negotiating Goals

Goals

In 2015, the US played a key role in negotiating the Paris Agreement, in which nations agreed to limit global warming to "well below 2°C" compared to preindustrial levels. However, in June 2017 the US announced it is withdrawing from the Paris Agreement and will cease implementation of its pledges to reduce carbon emissions. Under the terms of the Agreement, US withdrawal will take effect in November 2020. In the interim, the US may still participate in the negotiations. You must now decide on the following:

1. **US actions to reduce carbon emissions, if any.** In recent years US emissions have fallen, and are now ~14% below 2005 levels. However, US emissions are expected to grow modestly over time without further action and given current administration policies to promote production of oil, coal and natural gas. You can decide when US emissions will stop growing, when they will begin declining, and at what annual rate emissions decline, if at all.
2. **Whether to reduce deforestation or to increase reforestation or afforestation**.
3. **How much to contribute, if at all, to the Green Climate Fund.** The fund is intended to provide at least $100 billion/year by 2020 for developing countries to reduce their emissions and adapt to climate change. In June 2017, the US announced that it will no longer contribute to the Fund.

Context

The scientific consensus on climate is clear: over 97% of climate scientists agree that climate change is happening, that it is caused primarily by use of fossil fuels, and that the impacts could be devastating. US government research has shown that climate change is harming all 50 states today and that without dramatic reductions in global emissions, the damage will become far more severe.

Public Opinion

A majority of the public in the US believes climate change is real and that human activity contributes significantly to it. Most support policies that could be implemented to address climate change but oppose those actions that raise the cost of living. Climate change ranks near the bottom of most people's priorities, far below national security, the economy and jobs. Most people are opposed to any agreement that places undue burden on our own economy, while developing nations' emissions continue to grow. Meanwhile, entrenched fossil fuel interests continue to sow doubt about climate change causes and action.

Opportunities

Despite these challenges, the bipartisan "Risky Business" report (http://riskybusiness.org), endorsed by former US Treasury Secretaries of both parties, shows that the costs of delay are high while most states and regions in the US will benefit from emissions reductions policies.

National Action

In Paris, the US pledged a 26-28% reduction in US emissions from 2005 levels by 2025. Fulfilling this pledge depends on the successful implementation of federal policies to limit power plant emissions and improve fuel efficiency in cars, as well as action at the city, state and regional levels. However, the Administration is taking steps to roll back those federal policies while stating that fossil fuels will be needed for the foreseeable future. Its position is supported by some elected officials in the US Congress and business interests with a stake in the fossil fuel industry.

Forests and land use

Though we can pledge reductions in emissions from deforestation and land degradation (REDD) within our country, doing so would address only a small portion of US emissions.

Global Landscape

• China's economy is approximately equal in size to that of the US, is growing far faster than that of the US, and generates about 30% of global CO_2 emissions. In Paris, China pledged to peak its CO_2 emissions by 2030, when its emissions are projected to be >2.5 times those of the US, but made no commitment to reduce emissions. China must cut their emissions for the US to consider action.

• Meanwhile, emissions from rapidly developing countries, such as India, continue to grow. While the US remains committed to efforts to protect the environment, it cannot agree to targets that harm its economy. Without emissions cuts from other developing and less developed nations, by 2050, their combined emissions will rise to almost three times those of developed countries. Emissions from these nations must fall. It is not acceptable for these nations to demand that the US and other developed nations cut their emissions while they continue increase theirs.

• The less developed nations continue to emphasize that reductions in their emissions would require extensive financial assistance from developed countries, but corruption pervades many of these countries and financial assistance often fails to reach its intended use. They may also emphasize forestry policy over cutting fossil fuel emissions, which, while important, is insufficient for meeting the climate challenge.

CO_2 Emissions from Fossil Fuels and Cement

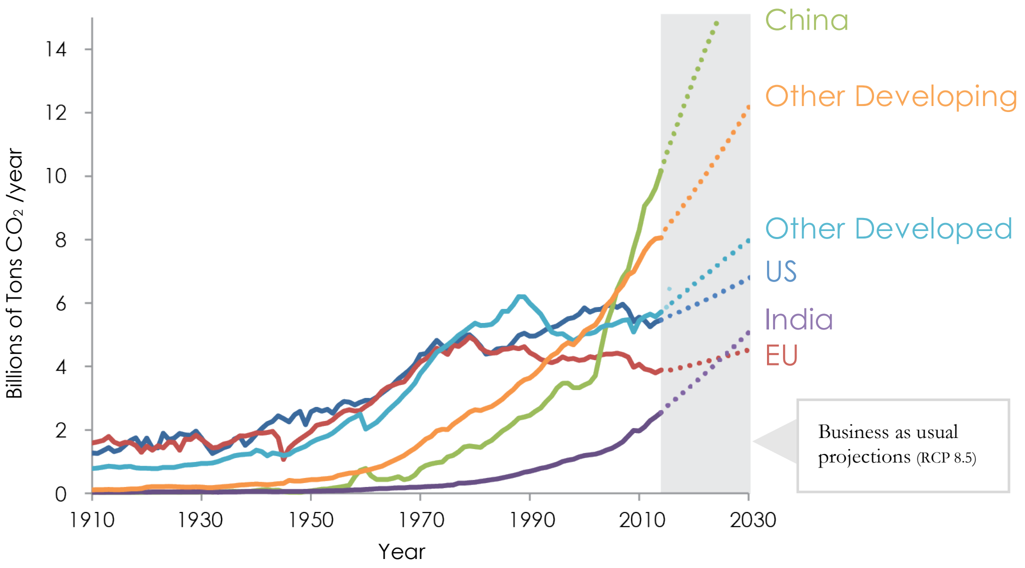

China is the world's largest emitter of CO_2. Without action, developing countries' emissions from fossil fuels are projected to more than triple by 2100.

Sweden sustained annual emissions reductions of 4.5% to reduce their dependence on oil (1976-1986). France and Belgium saw similar reductions around this time. Otherwise, most significant historical emission reductions have come from financial or political crises. According to UNEP, a 3.5% annual reduction rate is extremely ambitious.

GDP per person (2011 PPP US Dollars)

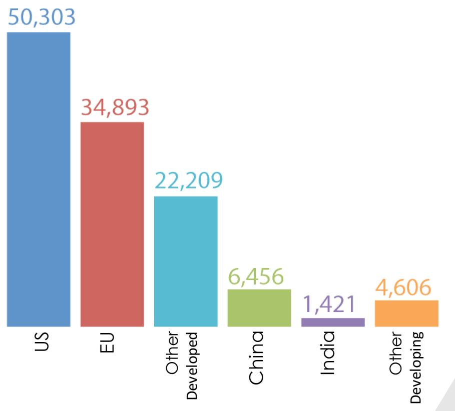

Population Wealth and Cumulative Emissions

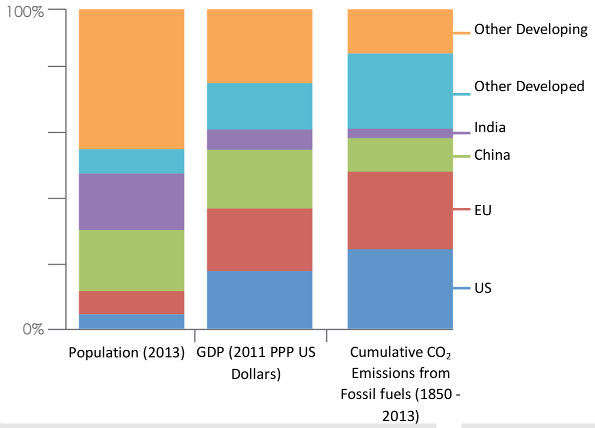

Emissions per person
2013 (tons CO_2 per year)

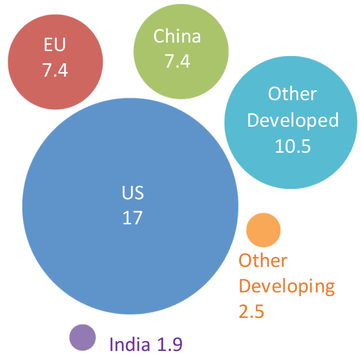

While cumulative emissions so far have been higher in the developed countries (i.e., the **US**, **EU**, and **other developed countries**), the growth of population, GDP per person, and emissions in the developing nations far outpaces growth in the developed countries. Under business-as-usual assumptions, cumulative emissions of all developed countries (**US**, **EU**, and **other developed**) are expected to fall to 37% of total by 2100.

Since 1980, emissions per person have risen dramatically in **China** and **India** (by 391% and 285%, respectively) but have fallen in the **US** and **Europe** (by 20% and 26%, respectively).
