# Supplementary material for: Combining role-play with interactive simulation to motivate informed climate action: Evidence from the World Climate simulation
Source: PLoS One. 2018 Aug 30;13(8):e0202877. doi: 10.1371/journal.pone.0202877 (PMC6117006; doi:10.1371/journal.pone.0202877)
Supplement: S2 Appendix — (DOCX) [file pone.0202877.s008.docx]

**Anonymous & Optional Assessment for the World Climate Exercise**

**Pre-Survey**

Soon you will participate in the World Climate or World Energy role-play simulation. Before you do, we ask you to complete this short survey. After the simulation we will ask you to complete another survey, in which you will also be asked to comment on what you thought about the exercise and how it affected you. Each survey will take about ten minutes to complete. The results will help us to improve the experience.

Your participation in the surveys is voluntary. Note that we ask for your name only to be able to match your pre- and post-simulation surveys to one another. Your name will then be deleted and all analysis will be done using the anonymous data. Surveys will be collected and processed by someone other than your instructor. Thank you for considering participating.

**Anonymous identifier** (we would like to use this code to pair pre- and post-surveys while maintaining participants’ anonymity):

What is the first letter of your mother’s/guardian’s first name? (e.g., “J” for Julie) _______

What is the first letter of the street where you live? (e.g., “P” for “Pleasant Street”): _______

What day of the month were you born on? (e.g., “20” for February 20): _______

**CLIMATE CHANGE - KNOWLEDGE**

1) Do you think that climate change is happening?

⭘ Yes ⭘ No ⭘ Don’t know

2) Assuming climate change is currently happening, do you think it is:

⭘ Caused mostly by human activities

⭘ Caused mostly by natural changes in the environment

⭘ Caused equally by both

⭘ Don’t know

3) How would you rate your knowledge about climate change?

⭘ I have no knowledge of climate change

⭘ Very limited; I have heard about it, but I am not

aware of the facts

⭘ I have gathered some information about the

subject

⭘ I have been studying the effects of climate change

⭘ I am an expert on climate change

4) Atmospheric carbon dioxide (CO_2_) concentrations are currently around 400 ppm (parts per million). Which of the following emissions pathways in Diagram 2 is most likely to meet the goal of stabilizing atmospheric CO_2_ concentrations around 450 ppm (Diagram 1)?

**Diagram 1 – Goal for CO_2_ in Atmosphere Diagram 2 – Potential Emission Pathways to Reach Goal**


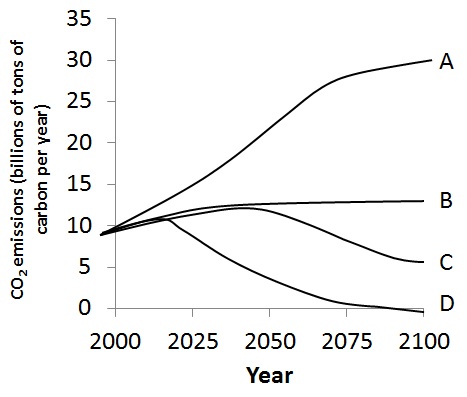

⭘ A

⭘ B

⭘ C

⭘ D

5)

Assuming climate change is happening, what are its expected impacts over the next few decades?

|  | Extremely likely | Somewhat likely | Somewhat unlikely | Not likely | I don’t know |
| --- | --- | --- | --- | --- | --- |
| 1. Increased temperatures globally. | ⭘ | ⭘ | ⭘ | ⭘ | ⭘ |
| 1. Decreased temperatures globally. | ⭘ | ⭘ | ⭘ | ⭘ | ⭘ |
| 1. An overall increase in clean, potable water globally. | ⭘ | ⭘ | ⭘ | ⭘ | ⭘ |
| 1. An overall decrease in clean, potable water globally. | ⭘ | ⭘ | ⭘ | ⭘ | ⭘ |
| 1. Increased incidence and intensity of heat waves. | ⭘ | ⭘ | ⭘ | ⭘ | ⭘ |
| 1. Increased rates of extinction of plant and animal species. | ⭘ | ⭘ | ⭘ | ⭘ | ⭘ |
| 1. An overall decrease in global food production. | ⭘ | ⭘ | ⭘ | ⭘ | ⭘ |
| 1. An overall increase in global food production. | ⭘ | ⭘ | ⭘ | ⭘ | ⭘ |
| 1. Increased global sea level. | ⭘ | ⭘ | ⭘ | ⭘ | ⭘ |
| 1. Increased intensity of storms across many regions. | ⭘ | ⭘ | ⭘ | ⭘ | ⭘ |
| 1. No changes beyond natural variability in weather. | ⭘ | ⭘ | ⭘ | ⭘ | ⭘ |

6) In order to meet the goal of limiting global warming to meet international goals of avoiding dangerous climate change, how much do you think it is necessary to annually reduce heat-trapping/greenhouse gas emissions if we start by 2020?

⭘ No reduction is necessary ⭘ 6-10% per year

⭘ Less than 1% per year ⭘ 11-30% per year

⭘ 1-2% per year ⭘ More than 30% per year

⭘ 2-5% per year

7) To what extent do you agree with the following statements?

|  | Strongly agree | Moderately agree | Neither agree nor disagree | Moderately disagree | Strongly disagree |
| --- | --- | --- | --- | --- | --- |
| 1. With the current rate of heat-trapping/greenhouse gas emissions, carbon dioxide levels will continue to increase because emission rates are higher than the rate at which carbon dioxide is being taken out of the atmosphere. | ⭘ | ⭘ | ⭘ | ⭘ | ⭘ |
| 1. It makes sense to wait and see what the climate impacts are before taking action. | ⭘ | ⭘ | ⭘ | ⭘ | ⭘ |
| 1. Changes in policies and energy systems are needed in order to effectively address climate change. | ⭘ | ⭘ | ⭘ | ⭘ | ⭘ |
| 1. New technologies will be developed that will solve the problems of climate change. | ⭘ | ⭘ | ⭘ | ⭘ | ⭘ |
| Using current technologies, it is possible to reduce emissions and address climate change. | ⭘ | ⭘ | ⭘ | ⭘ | ⭘ |
| 1. We could effectively address climate change if there were strong political and social will to do so. | ⭘ | ⭘ | ⭘ | ⭘ | ⭘ |
| 1. Successfully addressing climate change will also improve environmental health, beyond the effects of climate itself. | ⭘ | ⭘ | ⭘ | ⭘ | ⭘ |
| 1. Successfully addressing climate change will have a positive impact on social justice. | ⭘ | ⭘ | ⭘ | ⭘ | ⭘ |

8) When do you think it is necessary to begin reducing heat-trapping gas emissions in order to effectively address climate change?

⭘ It is not necessary to address climate change

⭘ More than 200 years

⭘ About 100 years

⭘ About 30 to 50 years

⭘ About 5 to 25 years

⭘ Right now

⭘ It is already too late

⭘ I don’t know

**CLIMATE CHANGE - AFFECT**

9) How worried are you about climate change?

⭘ Very worried ⭘ Somewhat worried ⭘ Not very worried ⭘ Not at all worried

10) Please rate how you feel about climate change. Fill in one bubble between each set of bipolar adjectives. Select the bubble that is in the direction of your feelings.

|  | | | | | | |
| --- | --- | --- | --- | --- | --- | --- |
| Hopeless | ⭘ | ⭘ | ⭘ | ⭘ | ⭘ | Hopeful |
| Discouraged | ⭘ | ⭘ | ⭘ | ⭘ | ⭘ | Empowered |
| Indifferent | ⭘ | ⭘ | ⭘ | ⭘ | ⭘ | Engaged |
| Not Guilty | ⭘ | ⭘ | ⭘ | ⭘ | ⭘ | Guilty |
| Calm | ⭘ | ⭘ | ⭘ | ⭘ | ⭘ | Outraged/Angry |
| Unconcerned | ⭘ | ⭘ | ⭘ | ⭘ | ⭘ | Alarmed |
| Not afraid at all | ⭘ | ⭘ | ⭘ | ⭘ | ⭘ | Very afraid |

11) How important is the issue of climate change to you personally?

⭘ Extremely important

⭘ Very important

⭘ Somewhat important

⭘ Not too important

⭘ Not at all important

**FUTURE ACTIONS**

12) How likely are you to do the following?

|  | Very likely | Somewhat likely | Unlikely | Will not do |
| --- | --- | --- | --- | --- |
| 1. Take action to reduce your personal carbon footprint (e.g., ride your bike more, turn off lights when they are not needed, purchase renewable energy sources if available from your utility, etc.). | ⭘ | ⭘ | ⭘ | ⭘ |
| 1. Discuss climate change with your family and friends. | ⭘ | ⭘ | ⭘ | ⭘ |
| 1. Discuss climate change with your peers. | ⭘ | ⭘ | ⭘ | ⭘ |
| 1. Take some form of political action (e.g., write to your government officials, sign a petition, participate in a town hall meeting or rally) in support of climate change policy. | ⭘ | ⭘ | ⭘ | ⭘ |

**BACKGROUND**

13) Have you participated in the World Climate exercise before?

⭘ Yes ⭘ No

14) What is your gender?

⭘ Female ⭘ Male ⭘ Other/don’t wish to specify

15) What is your age?

(for middle and high school): ⭘ Up to Grade 8 ⭘ Grade 9-12

(for adults): ⭘ 18-24 ⭘ 25-35 ⭘ 36-50 ⭘ 51-75 ⭘ 76+

16) What is your home country? _________________________________

17) What is the highest level of education achieved by one or both of your parents or guardians?

⭘ No school

- Elementary school only

⭘ Secondary (high) school

⭘ Some postsecondary (college) education

- Bachelor’s degree or beyond

18) What is the highest level of education that you have achieved?

⭘ No high school degree

- Secondary (high school) degree

⭘ Some postsecondary (college) education

- Bachelor’s degree or beyond

19) Were you in a science-related major in your postsecondary education?

⭘ Yes ⭘ No


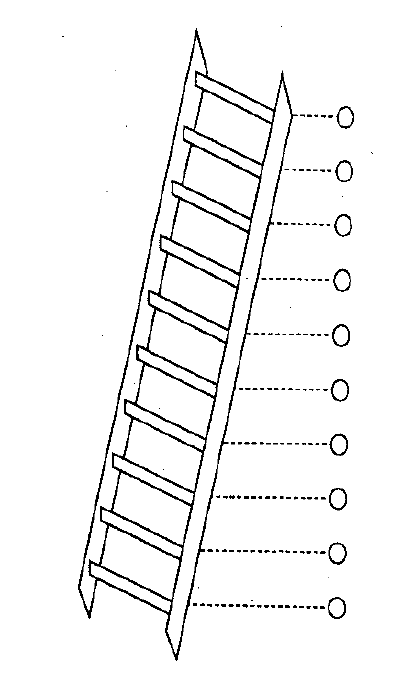


20) Think of this ladder as representing where people stand in your country. At the top of the ladder are people who are best off—those who have the most money, the most education, and the most respected jobs. At the bottom are the people who are worst off—who have the least money, the least education, and the least respected jobs or no job. The higher up you are on this ladder, the closer you are to the people at the very top; the lower you are, the closer you are to the people at the very bottom. When considering your overall life circumstances, where would you place yourself on this ladder? **Please fill in the circle that best represents where you stand at this time in your life compared to other people in your country.**

21) To what extent are you in favor of the government placing regulations on the free market?

⭘ Strongly in favor

- Somewhat in favor

⭘ Neutral

⭘ Somewhat opposed

⭘ Strongly opposed

**Anonymous & Optional Assessment for the World Climate Exercise**

**Post-Survey**

Thank you for participating in the World Climate or World Energy role-play simulation. We now ask you to complete this short survey about your understanding of climate change. This survey will take about 20 minutes to complete. The results will be compared to those from other individuals who have participated in the workshop to gain a better picture of its impact and will help us to improve the experience.

Your participation in the survey is voluntary. We ask for your name only to be able to match your pre- and post-surveys to one another. Your name will then be deleted and all analysis will be done using the anonymous data. Surveys will be collected and processed by someone other than your instructor. Thank you very much for participating.

**Anonymous identifier** (we would like to use this code to pair pre- and post-surveys while maintaining participants’ anonymity):

What is the first letter of your mother’s/guardian’s first name? (e.g., “J” for Julie) _______

What is the first letter of the street where you live? (e.g., “P” for “Pleasant Street”): _______

What day of the month were you born on? (e.g., “20” for February 20): _______

**CLIMATE CHANGE - KNOWLEDGE**

1) Do you think that climate change is happening?

⭘ Yes ⭘ No ⭘ Don’t know

2) Assuming climate change is currently happening, do you think it is:

⭘ Caused mostly by human activities

⭘ Caused mostly by natural changes in the environment

⭘ Caused equally by both

⭘ Don’t know

3) How would you rate your knowledge about climate change?

⭘ I have no knowledge of climate change

⭘ Very limited; I have heard about it, but I am not

aware of the facts

⭘ I have gathered some information about the

subject

⭘ I have been studying the effects of climate change

⭘ I am an expert on climate change

4) Atmospheric carbon dioxide (CO_2_) concentrations are currently around 400 ppm (parts per million). Which of the following emissions pathways in Diagram 2 is most likely to meet the goal of stabilizing atmospheric CO_2_ concentrations around 450 ppm (Diagram 1)?

**Diagram 1 – Goal for CO_2_ in Atmosphere Diagram 2 – Potential Emission Pathways to Reach Goal**


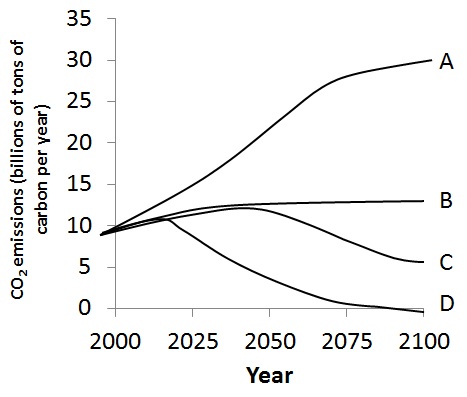

⭘ A

⭘ B

⭘ C

⭘ D

5)

Assuming climate change is happening, what are its expected impacts over the next few decades?

|  | Extremely likely | Somewhat likely | Somewhat unlikely | Not likely | I don’t know |
| --- | --- | --- | --- | --- | --- |
| 1. Increased temperatures globally. | ⭘ | ⭘ | ⭘ | ⭘ | ⭘ |
| 1. Decreased temperatures globally. | ⭘ | ⭘ | ⭘ | ⭘ | ⭘ |
| 1. An overall increase in clean, potable water globally. | ⭘ | ⭘ | ⭘ | ⭘ | ⭘ |
| 1. An overall decrease in clean, potable water globally. | ⭘ | ⭘ | ⭘ | ⭘ | ⭘ |
| 1. Increased incidence and intensity of heat waves. | ⭘ | ⭘ | ⭘ | ⭘ | ⭘ |
| 1. Increased rates of extinction of plant and animal species. | ⭘ | ⭘ | ⭘ | ⭘ | ⭘ |
| 1. An overall decrease in global food production. | ⭘ | ⭘ | ⭘ | ⭘ | ⭘ |
| 1. An overall increase in global food production. | ⭘ | ⭘ | ⭘ | ⭘ | ⭘ |
| 1. Increased global sea level. | ⭘ | ⭘ | ⭘ | ⭘ | ⭘ |
| 1. Increased intensity of storms across many regions. | ⭘ | ⭘ | ⭘ | ⭘ | ⭘ |
| 1. No changes beyond natural variability in weather. | ⭘ | ⭘ | ⭘ | ⭘ | ⭘ |

6) In order to meet the goal of limiting global warming to meet international goals of avoiding dangerous climate change, how much do you think it is necessary to annually reduce heat-trapping/greenhouse gas emissions if we start by 2020?

⭘ No reduction is necessary ⭘ 6-10% per year

⭘ Less than 1% per year ⭘ 11-30% per year

⭘ 1-2% per year ⭘ More than 30% per year

⭘ 2-5% per year

7) To what extent do you agree with the following statements?

|  | Strongly agree | Moderately agree | Neither agree nor disagree | Moderately disagree | Strongly disagree |
| --- | --- | --- | --- | --- | --- |
| 1. With the current rate of heat-trapping/greenhouse gas emissions, carbon dioxide levels will continue to increase because emission rates are higher than the rate at which carbon dioxide is being taken out of the atmosphere. | ⭘ | ⭘ | ⭘ | ⭘ | ⭘ |
| 1. It makes sense to wait and see what the climate impacts are before taking action. | ⭘ | ⭘ | ⭘ | ⭘ | ⭘ |
| 1. Changes in policies and energy systems are needed in order to effectively address climate change. | ⭘ | ⭘ | ⭘ | ⭘ | ⭘ |
| 1. New technologies will be developed that will solve the problems of climate change. | ⭘ | ⭘ | ⭘ | ⭘ | ⭘ |
| Using current technologies, it is possible to reduce emissions and address climate change. | ⭘ | ⭘ | ⭘ | ⭘ | ⭘ |
| 1. We could effectively address climate change if there were strong political and social will to do so. | ⭘ | ⭘ | ⭘ | ⭘ | ⭘ |
| 1. Successfully addressing climate change will also improve environmental health, beyond the effects of climate itself. | ⭘ | ⭘ | ⭘ | ⭘ | ⭘ |
| 1. Successfully addressing climate change will have a positive impact on social justice. | ⭘ | ⭘ | ⭘ | ⭘ | ⭘ |

8) When do you think it is necessary to begin reducing heat-trapping gas emissions in order to effectively address climate change?

⭘ It is not necessary to address climate change

⭘ More than 200 years

⭘ About 100 years

⭘ About 30 to 50 years

⭘ About 5 to 25 years

⭘ Right now

⭘ It is already too late

⭘ I don’t know

**CLIMATE CHANGE - AFFECT**

9) How worried are you about climate change?

⭘ Very worried ⭘ Somewhat worried ⭘ Not very worried ⭘ Not at all worried

10) Please rate how you feel about climate change. Fill in one bubble between each set of bipolar adjectives. Select the bubble that is in the direction of your feelings.

|  | | | | | | |
| --- | --- | --- | --- | --- | --- | --- |
| Hopeless | ⭘ | ⭘ | ⭘ | ⭘ | ⭘ | Hopeful |
| Discouraged | ⭘ | ⭘ | ⭘ | ⭘ | ⭘ | Empowered |
| Indifferent | ⭘ | ⭘ | ⭘ | ⭘ | ⭘ | Engaged |
| Not Guilty | ⭘ | ⭘ | ⭘ | ⭘ | ⭘ | Guilty |
| Calm | ⭘ | ⭘ | ⭘ | ⭘ | ⭘ | Outraged/Angry |
| Unconcerned | ⭘ | ⭘ | ⭘ | ⭘ | ⭘ | Alarmed |
| Not afraid at all | ⭘ | ⭘ | ⭘ | ⭘ | ⭘ | Very afraid |

11) How important is the issue of climate change to you personally?

⭘ Extremely important

⭘ Very important

⭘ Somewhat important

⭘ Not too important

⭘ Not at all important

**FUTURE ACTIONS**

12) How likely are you to do the following?

|  | Very likely | Somewhat likely | Unlikely | Will not do |
| --- | --- | --- | --- | --- |
| a) Take action to reduce your personal carbon footprint (e.g., ride your bike more, turn off lights when they are not needed, purchase renewable energy sources if available from your utility, etc.). | ⭘ | ⭘ | ⭘ | ⭘ |
| b) Discuss climate change with your family and friends. | ⭘ | ⭘ | ⭘ | ⭘ |
| c) Discuss climate change with your peers. | ⭘ | ⭘ | ⭘ | ⭘ |
| d) Take some form of political action (e.g., write to your government officials, sign a petition, participate in a town hall meeting or rally) in support of climate change policy. | ⭘ | ⭘ | ⭘ | ⭘ |

**REACTIONS TO THE WORLD CLIMATE EXERCISE**

13) To what extent do you agree with the following statements?

|  | Strongly agree | Moderately agree | Neither agree nor disagree | Moderately disagree | Strongly disagree |
| --- | --- | --- | --- | --- | --- |
| 1. The World Climate exercise was engaging. | ⭘ | ⭘ | ⭘ | ⭘ | ⭘ |
| 1. I plan to tell others about this experience. | ⭘ | ⭘ | ⭘ | ⭘ | ⭘ |
| 1. Through this experience, I gained a better understanding of different countries' perspectives on climate change. | ⭘ | ⭘ | ⭘ | ⭘ | ⭘ |
| 1. I cared about the outcomes of the exercise. | ⭘ | ⭘ | ⭘ | ⭘ | ⭘ |
| 1. I identified with my role in the exercise. | ⭘ | ⭘ | ⭘ | ⭘ | ⭘ |
| 1. This exercise was a good learning experience. | ⭘ | ⭘ | ⭘ | ⭘ | ⭘ |
| 1. As a result of this experience, I have a sense of urgency to take action to combat climate change. | ⭘ | ⭘ | ⭘ | ⭘ | ⭘ |
| 1. As a result of this experience, I want to learn more about leading or effecting change in the area of climate change. | ⭘ | ⭘ | ⭘ | ⭘ | ⭘ |
| 1. This experience offered opportunities for meeting new people or developing collaborations with other participants. | ⭘ | ⭘ | ⭘ | ⭘ | ⭘ |

14) How has participation in the exercise affected your desire to learn more about the following?

|  | More interested | No change | Less interested |
| --- | --- | --- | --- |
| The science of climate change | ⭘ | ⭘ | ⭘ |
| Potential solutions for mitigating the effects of climate change | ⭘ | ⭘ | ⭘ |
| Politics as it relates to climate change | ⭘ | ⭘ | ⭘ |
| Economics as it relates to climate change | ⭘ | ⭘ | ⭘ |
| Energy policies | ⭘ | ⭘ | ⭘ |

15) How has your motivation to take action to address climate change been affected by your participation in the exercise?

⭘ Increased a lot

⭘ Increased a little

⭘ Stayed high

⭘ Stayed low

⭘ Decreased a little

⭘ Decreased a lot

**BACKGROUND**

16) Have you participated in the World Climate exercise before?

⭘ Yes ⭘ No

17) What is your gender?

⭘ Female ⭘ Male ⭘ Other/don’t wish to specify

18) What is your age?

(for middle and high school): ⭘ Up to Grade 8 ⭘ Grade 9-12

(for adults): ⭘ 18-24 ⭘ 25-35 ⭘ 36-50 ⭘ 51-75 ⭘ 76+

19) What is your home country? _________________________________

20) What is the highest level of education achieved by one or both of your parents or guardians?

⭘ No school

- Elementary school only

⭘ Secondary (high) school

⭘ Some postsecondary (college) education

- Bachelor’s degree or beyond

21) What is the highest level of education that you have achieved?

⭘ No high school degree

- Secondary (high school) degree

⭘ Some postsecondary (college) education

- Bachelor’s degree or beyond

22) Were you in a science-related major in your postsecondary education?

⭘ Yes ⭘ No


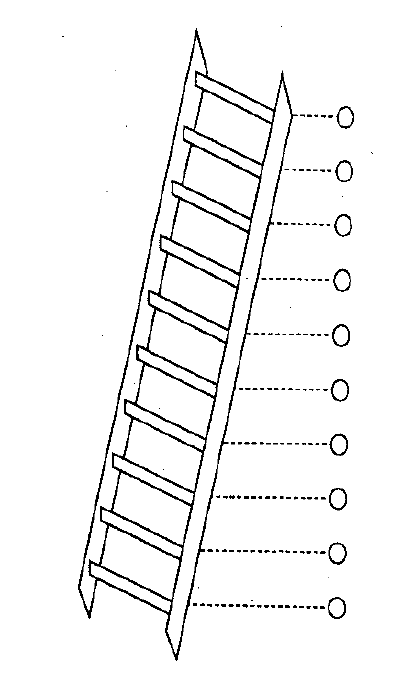


23) Think of this ladder as representing where people stand in your country. At the top of the ladder are people who are best off—those who have the most money, the most education, and the most respected jobs. At the bottom are the people who are worst off—who have the least money, the least education, and the least respected jobs or no job. The higher up you are on this ladder, the closer you are to the people at the very top; the lower you are, the closer you are to the people at the very bottom. When considering your overall life circumstances, where would you place yourself on this ladder? **Please fill in the circle that best represents where you stand at this time in your life compared to other people in your country.**

24) To what extent are you in favor of the government placing regulations on the free market?

⭘ Strongly in favor

- Somewhat in favor

⭘ Neutral

⭘ Somewhat opposed

⭘ Strongly opposed

**OPEN-ENDED QUESTIONS (OPTIONAL)**

25) How has participating in the World Climate Exercise affected your understanding of climate change, if at all?

26) Was there anything about World Climate that surprised you (such as what you took away from the experience or what happened during the exercise)?  Why or why not?

27) How did participating in the World Climate Exercise make you feel?  Why?

28) Has participating in World Climate affected how motivated you are to address climate change?  If so, what do you plan to do?

29) What was the best aspect of the World Climate Exercise?

30) How would you improve the World Climate Exercise?
